# Observation versus screening spinal MRI and pre-emptive treatment for spinal cord compression in patients with castration-resistant prostate cancer and spinal metastases in the UK (PROMPTS): an open-label, randomised, controlled, phase 3 trial

**DOI:** 10.1016/S1470-2045(22)00092-4

**Published:** 2022-04

**Authors:** David Dearnaley, Victoria Hinder, Adham Hijab, Gail Horan, Narayanan Srihari, Philip Rich, J Graeme Houston, Ann M Henry, Stephanie Gibbs, Ram Venkitaraman, Clare Cruickshank, Shama Hassan, Alec Miners, Malcolm Mason, Ian Pedley, Heather Payne, Susannah Brock, Robert Wade, Angus Robinson, Omar Din, Kathryn Lees, John Graham, Jane Worlding, Julia Murray, Chris Parker, Clare Griffin, Aslam Sohaib, Emma Hall, PROMPTS investigators

**Affiliations:** a Division of Radiotherapy and Imaging, The Institute of Cancer Research, London, UK; b Clinical Trials and Statistics Unit, The Institute of Cancer Research, London, UK; c Urology Unit, Royal Marsden NHS Foundation Trust, London, UK; d Clinical Oncology, The Queen Elizabeth Hospital King's Lynn NHS Foundation Trust, King's Lynn, UK; e Clinical Oncology, The Shrewsbury and Telford Hospital NHS Trust, Shrewsbury, UK; f Radiology, St George's University Hospitals NHS Foundation Trust, London, UK; g Imaging Science and Technology, University of Dundee, Dundee, UK; h Clinical Oncology, University of Leeds, Leeds, UK; i Clinical Oncology, Barking, Havering and Redbridge University Hospitals NHS Trust, London, UK; j Clinical Oncology, East Suffolk and North Essex NHS Foundation Trust, Ipswich, UK; k Department of Health Services Research and Policy, London School of Hygiene and Tropical Medicine, London, UK; l Clinical Oncology, Cardiff University, Cardiff, UK; m Clinical Oncology, The Newcastle upon Tyne Hospitals NHS Foundation Trust, Newcastle, UK; n Clinical Oncology, University College London Hospitals NHS Foundation Trust, London, UK; o Clinical Oncology, University Hospitals Dorset NHS Foundation Trust, Poole, UK; p Clinical Oncology, Norfolk and Norwich University Hospitals NHS Foundation Trust, Norwich, UK; q Clinical Oncology, Brighton and Sussex University Hospitals NHS Trust, Brighton, UK; r Clinical Oncology, Sheffield Teaching Hospitals NHS Foundation Trust, Sheffield, UK; s Clinical Oncology, Maidstone and Tunbridge Wells NHS Trust, Maidstone, UK; t Clinical Oncology, Somerset NHS Foundation Trust, Taunton, UK; u Oncology, University Hospitals Coventry and Warwickshire NHS Trust, Coventry, UK

## Abstract

**Background:**

Early diagnosis of malignant spinal cord compression (SCC) is crucial because pretreatment neurological status is the major determinant of outcome. In metastatic castration-resistant prostate cancer, SCC is a clinically significant cause of disease-related morbidity and mortality. We investigated whether screening for SCC with spinal MRI, and pre-emptive treatment if radiological SCC (rSCC) was detected, reduced the incidence of clinical SCC (cSCC) in asymptomatic patients with metastatic castration-resistant prostate cancer and spinal metastasis.

**Methods:**

We did a parallel-group, open-label, randomised, controlled, phase 3, superiority trial. Patients with metastatic castration-resistant prostate cancer were recruited from 45 National Health Service hospitals in the UK. Eligible patients were aged at least 18 years, with an Eastern Co-operative Oncology Group performance status of 0–2, asymptomatic spinal metastasis, no previous SCC, and no spinal MRI in the past 12 months. Participants were randomly assigned (1:1), using a minimisation algorithm with a random element (balancing factors were treatment centre, alkaline phosphatase [normal *vs* raised, with the upper limit of normal being defined at each participating laboratory], number of previous systemic treatments [first-line *vs* second-line or later], previous spinal treatment, and imaging of thorax and abdomen), to no MRI (control group) or screening spinal MRI (intervention group). Serious adverse events were monitored in the 24 h after screening MRI in the intervention group. Participants with screen-detected rSCC were offered pre-emptive treatment (radiotherapy or surgical decompression was recommended per treating physician's recommendation) and 6-monthly spinal MRI. All patients were followed up every 3 months, and then at month 30 and 36. The primary endpoint was time to and incidence of confirmed cSCC in the intention-to-treat population (defined as all patients randomly assigned), with the primary timepoint of interest being 1 year after randomisation. The study is registered with ISRCTN, ISRCTN74112318, and is now complete.

**Findings:**

Between Feb 26, 2013, and April 25, 2017, 420 patients were randomly assigned to the control (n=210) or screening MRI (n=210) groups. Median age was 74 years (IQR 68 to 79), 222 (53%) of 420 patients had normal alkaline phosphatase, and median prostate-specific antigen concentration was 48 ng/mL (IQR 17 to 162). Screening MRI detected rSCC in 61 (31%) of 200 patients with assessable scans in the intervention group. As of data cutoff (April 23, 2020), at a median follow-up of 22 months (IQR 13 to 31), time to cSCC was not significantly improved with screening (hazard ratio 0·64 [95% CI 0·37 to 1·11]; Gray's test p=0·12). 1-year cSCC rates were 6·7% (95% CI 3·8–10·6; 14 of 210 patients) for the control group and 4·3% (2·1–7·7; nine of 210 patients) for the intervention group (difference −2·4% [95% CI −4·2 to 0·1]). Median time to cSCC was not reached in either group. No serious adverse events were reported within 24 h of screening.

**Interpretation:**

Despite the substantial incidence of rSCC detected in the intervention group, the rate of cSCC in both groups was low at a median of 22 months of follow-up. Routine use of screening MRI and pre-emptive treatment to prevent cSCC is not warranted in patients with asymptomatic castration-resistant prostate cancer with spinal metastasis.

**Funding:**

Cancer Research UK.

**Research in context**

**Evidence before this study**

We searched PubMed for articles in English published between Jan 1, 1970, and Dec 31, 2012, before trial commencement, using the terms “spinal cord compression”, “cancer”, “prostate cancer”, “magnetic resonance imaging”, “radiotherapy”, “spinal surgery”, “systematic review”, AND “guideline” and then repeated the search for publications up to April 1, 2021. Systematic reviews and international guidelines have recognised the importance of early diagnosis and intervention of spinal cord compression (SCC). Spinal MRI is recommended with subsequent intervention with surgical decompression or radiotherapy. Institutional studies suggest that spinal MRI can detect asymptomatic early radiological SCC (rSCC) in patients with castration-resistant metastatic prostate cancer and early intervention with radiotherapy substantially reduces the development of clinical SCC (cSCC). National Institute for Health and Care Excellence (NICE) guidance does not recommend spinal MRI and treatment intervention for asymptomatic patients with spinal metastases, but randomised trials to assess early diagnostic and intervention strategies were encouraged.

**Added value of this study**

To our knowledge, this is the first multicentre randomised, controlled trial to study the role of screening spinal MRI to detect radiologically defined asymptomatic spinal cord compression (rSCC) in men with castration-resistant metastatic prostate cancer. We confirmed the reproducibility of an MRI epidural spinal cord compression (ESCC) scale. We showed that radiotherapy was efficacious in preventing progression of rSCC to symptomatic cSCC. However, although the intervention group had a lower risk of developing cSCC than the control group, the difference between the groups was not significant. Patients with rSCC had a high risk of progression to cSCC at other spinal sites. Although the resources needed for spinal MRI and radiotherapy were higher in the intervention group than in the control group, there was a decrease in the use of subsequent additional systemic treatments. We were unable to identify predictive factors for the development of rSCC or cSCC.

**Implications of all the available evidence**

Spinal MRI can reliably detect rSCC in castration-resistant metastatic prostate cancer, but early rSCC does not usually progress to cSCC in patient groups who have access to contemporary systemic treatment. We recommend that the ESCC scale be introduced into routine clinical practice because it can be used to determine the presence of early rSCC and identifies a high-risk group for subsequent development of cSCC. Close adherence to NICE guidelines for the early investigation of spinal symptoms is important in reducing neurological disability. We do not recommend screening spinal MRI in unselected patients with castration-resistant metastatic prostate cancer, but further research to identify high-risk groups is warranted.

## Introduction

Malignant spinal cord compression (SCC) and its complications have a profound effect on functional status and quality of life with a resulting increased burden on the health-care system.^1^ Early diagnosis is crucial because pre-treatment neurological status is the major determinant influencing outcome. Almost all patients with SCC who are ambulatory before treatment retain motor function.^2^, ^3^, ^4^, ^5^, ^6^, ^7^, ^8^

Metastatic castration-resistant prostate cancer predominantly involves the skeleton, and a substantial proportion of disease-related morbidity and mortality is attributable to skeletal-related events. SCC is the most clinically significant skeletal-related event^1^ of metastatic castration-resistant prostate cancer and prostate cancer accounts for about 20% of all cases of SCC.^4^, ^8^, ^9^ Population-based studies indicate that SCC occurs in about 7% of cases of lethal prostate cancer,^9^ although findings from a systematic review showed that up to 24% of patients with metastatic prostate cancer developed SCC.^7^ In prostate cancer, early radiological signs of impending SCC (radiological SCC [rSCC]) can be detected in 27–32% of asymptomatic patients using spinal MRI.^10^, ^11^ National Institute for Health and Care Excellence (NICE) guidance^8^ advises that serial MRI to detect SCC should only be done as part of a randomised controlled trial and that neither radiotherapy nor surgery should be used to treat asymptomatic spinal metastases to prevent SCC unless part of such a trial. To address these issues, we did a randomised, controlled, phase 3 study to determine the role of screening MRI to detect rSCC with subsequent pre-emptive treatment to sites of rSCC.

## Methods

### Study design and participants

PROMPTS is a multicentre, parallel-group, open-label, randomised, controlled, phase 3 trial undertaken at 45 National Health Service (NHS) hospitals in the UK (appendix p 5). The aims were to assess the value of screening spinal MRI in men with metastatic castration-resistant prostate cancer with bone involvement to detect and treat asymptomatic SCC.

Eligible patients were aged 18 years and older, had a confirmed pathological diagnosis of prostate adenocarcinoma or a clinical diagnosis of prostate cancer with osteoblastic bone metastases and a serum prostate-specific antigen (PSA) concentration of 100 ng/mL or higher at any time between diagnosis and randomisation. Other inclusion criteria were the presence of asymptomatic spinal metastasis, castration-resistant state (defined as PSA >5 ng/dL and more than 50% increase above the nadir during treatment with a luteinising hormone-releasing hormone analogue or after orchidectomy), PSA concentration of more than 5 ng/mL within 21 days before randomisation, life expectancy of 6 months or longer, and Eastern Co-operative Oncology Group (ECOG) performance status of 0–2. Key exclusion criteria were presence of any back pain or neurological symptoms from spinal metastases, previous spinal MRI within 12 months from trial entry, previous external beam radiotherapy to the vertebrae or spinal surgery to treat SCC, and any contraindication for MRI. A full list of exclusion criteria is in the protocol (appendix). Patients were recruited by their clinical care teams and provided written, informed consent before enrolment.

The trial was approved by the London Queen Square Multi-centre Research Ethics Committee (12/LO1109), sponsored by The Institute of Cancer Research (ICR), and conducted in accordance with the principles of good clinical practice. The ICR Clinical Trials and Statistics Unit (ICR-CTSU; London, UK) coordinated the study and carried out central statistical data monitoring and all analyses. The study protocol is available in the appendix.

### Randomisation and masking

Patients were randomly assigned (1:1) to either no MRI (control group) or to screening MRI (intervention group). Allocation was done centrally by the ICR-CTSU using a minimisation algorithm incorporating an 80% random element; balancing factors were treatment centre, alkaline phosphatase (ALP; normal *vs* raised, with the upper limit of normal defined by each participating laboratory), number of previous systemic treatments (first-line *vs* second-line or later), previous spinal surgery or radiotherapy for metastatic disease (yes *vs* no), and—following a protocol amendment on April 8, 2015—CT or PET-CT of the thorax and abdomen within the past 6 months (yes *vs* no). No one was masked to study group assignment because of the impracticality of performing sham MRI.

### Procedures

Baseline investigations included medical history, PSA measurement, full blood count including serum creatinine, ALP, and serum albumin. Neurological assessment was based on the Frankel scale,^12^ which is a five-point standardised neurological assessment tool that is used after spinal cord injury (appendix p 3). Pre-trial clinical signs and symptoms were also recorded and graded using the National Cancer Institute's Common Terminology Criteria for Adverse Event (CTCAE) version 4.0. Patient-reported outcomes were also collected at baseline with European Organisation for Research and Treatment of Cancer (EORTC) QLQ-C30,^13^ EQ-5D-5L,^14^ Brief Pain Inventory (BPI),^15^ and Hospital Anxiety and Depression score (HADS).^16^

In the intervention group, screening spinal MRI was done within 4 weeks of randomisation (baseline) using a minimum field strength of 1 T with a spinal coil. The whole spine was imaged from the base of skull to the coccyx with sagittal T1 and T2 weighted images. Sagittal images were supplemented with selected axial images through any suspicious areas at the discretion of the radiologist. Scans were assessed by the local specialist radiologist using a seven-point epidural spinal cord compression (ESCC) scoring system (appendix p 4), based on the Bilsky scoring system, to determine presence of rSCC.^3^, ^6^, ^17^ Each vertebra was individually assessed. Patients were assessed as having rSCC when no neurological symptoms were detectable in the presence of epidural disease, whereas patients with neurological symptoms in the presence of epidural disease were deemed to have clinical SCC (cSCC).

In the intervention group, if the baseline screening MRI was positive for rSCC, pre-emptive treatment was recommended with radiotherapy or surgical decompression. After treatment, patients had a follow-up MRI every 6 months.

Participants in both groups were followed up at 3-monthly intervals for the first 2 years and then at 30 and 36 months, as well as at the time of any cSCC episode. Assessments included neurological status using the Frankel score,^12^ patient-reported outcomes (HADS was repeated at 3 months only), and PSA, as well as recording of new treatments and all spinal MRIs, as requested by their treating physician. Patients were followed up beyond 36 months, until death or loss to follow-up.

Serious adverse events were collected for a 24 h period after the screening MRI scan in the intervention group using the CTCAE version 4.0. In the intervention group, adverse events, EQ-5D-5L, and BPI were assessed before and after (not BPI) any pre-emptive treatment. All patient-reported outcomes were completed on paper by the patient at their clinic visit. The main outcome of interest was EORTC QLQ-C30 physical functioning. Additional patient-reported outcome scores reported were: EORTC QLQ-C30: functional scales, global health, and pain; BPI: severity and interference; HADS: anxiety and depression; and EQ-5D-5L: heath state today. Adverse events during follow-up (graded using CTCAE) were only collected after treatment for rSCC or cSCC.

If new neurological symptoms suggestive of cSCC or new onset clinically significant back pain developed, spinal MRI was done, ideally within 24 h of onset, in accordance with NICE and local guidelines, regardless of randomisation group. All MRI scans leading to a diagnosis of rSCC or cSCC and a minimum 10% random sample of negative baseline scans were centrally reviewed (by AS, PR, JGH) and iterative feedback given to participating radiologists and oncologists.

For both study groups, the protocol recommended rSCC was treated (pre-emptively) with radiotherapy or surgery and that NICE guidelines^8^ were followed for cSCC. Short courses of high-dose corticosteroids (eg, dexamethasone 8–24 mg total dose per day) were permitted. Radiotherapy was to be delivered within 1 week of diagnosis of rSCC and within 48 h of diagnosis of cSCC. The recommended radiotherapy dose was 20 Gy given daily in five fractions, prescribed to at least the mid-point of the spinal cord or cauda equina. Radiotherapy was to be planned by conventional or CT-based virtual simulation using MRI information to determine the level and length of the radiotherapy field, which should extend to at least one or more vertebral level beyond the site or sites of rSCC or cSCC.

### Outcomes

The primary outcome was incidence of and time to development of confirmed cSCC, with the timepoint of primary interest being 1 year after randomisation, according to local assessment. Participants were considered to have developed cSCC if they had a compromised Frankel score (ie, grade A–D) with supportive radiological findings. If there was diagnostic uncertainty, cases were centrally reviewed (by AH or JM) without knowledge of randomisation group using available data from MRI, clinical and patient-reported outcomes, and radiotherapy or surgical records.

Secondary outcomes were rate of detection of rSCC (Bilsky score [ie, ESCC score] of 1a–3) on baseline screening MRI (intervention group only) according to local assessment; 1 year and 2 year incidences and time (from randomisation) to functional neurological deficit (FND; Frankel score grade A–D) and irreversible FND (defined as Frankel score not returning to normal [grade E] after 3 and 6 months); incidence of any SCC (rSCC and cSCC ESCC 1–3) at 1 year after randomisation according to local assessment; overall survival; cost effectiveness; pain (using the short-form BPI); and patient-reported outcomes at 1 year after randomisation. Cost-effectiveness analyses were not done in light of primary results.

### Statistical analysis

We estimated a 1-year cSCC incidence of 15·6% in the control group based on a baseline rSCC prevalence of 12·9% (calculated as the average rSCC rate reported in asymptomatic patients in retrospective studies^10^, ^11^), median overall survival of 19 months,^18^ and assuming all participants with rSCC and 3·2% of those without rSCC at screening would develop cSCC by 1 year if untreated.^10^ A hazard ratio (HR) of 0·48 would be equivalent to a reduction in 1 year cSCC rate to 7·8% in the intervention group. Sample size calculations were based on a log-rank test with 5% two-sided significance. With 90% power, the original target sample size was 541 patients. In November, 2016, the statistical power was reduced to 85% to allow for timely completion of recruitment. The revised sample size of 414 patients (71 events) was based on uniform accrual over a 4 year period and a minimum of 1 year of follow-up for all participants. No adjustment for non-compliance with screening MRI was done.

A formal pre-planned interim analysis was planned to take place after 54 patients in the intervention group had their baseline MRI. If the rSCC rate was 10% or higher, recruitment continued.

We did all analyses in the intention-to-treat population (defined as all patients who were randomly assigned to study groups). To account for death as a competing risk for outcomes relating to rSCC, we estimated cSCC and FND incidences using the cumulative incidence function, and we used Gray's test^19^ to compare study groups. We estimated the intervention effect using unadjusted and adjusted subdistribution models. Baseline covariates included in all adjusted models were balancing factors and time since development of castration-resistant prostate cancer, time since start of continuous hormone treatment, ECOG performance status (0, 1, and 2), and natural logrithm of PSA (ln PSA) concentration. HRs of less than 1 indicate a decreased risk of the event in the intervention group compared with in the control group. We also fitted the subdistribution model for death (in the presence of cSCC) to ensure results from the cSCC analysis were not due to differences seen in the numbers of deaths between the study groups. We also fitted cause-specific regression models for cSCC with death as a competing risk, and for death with cSCC as a competing risk to provide further comparisons. We censored patients without cSCC at the date of last follow-up. We classified patients who died before having cSCC as having a competing event at the date of death. We report the distribution (prespecified) of ESCC scores at the time of SCC outcome events (rSCC and cSCC) and Frankel scores at the time of cSCC outcome events.

We calculated the incidence of rSCC at screening in the intervention group using binomial proportions and 95% CIs. We used logistic regression to identify clinical predictors of rSCC on the screening MRI. To assess non-proportionality of covariates in time-to-event multivariable analysis, we considered the time dependency of all prespecified baseline covariates (ie, balancing factors and time since development of castration-resistant prostate cancer, time since start of continuous hormone treatment, ECOG performance status [0, 1, and 2], and PSA concentration). Incidence of any SCC was compared using Gray's test and 6 months (post hoc) and 12 months (prespecified). Comparisons were made between control patients and screen-negative patients in the intervention group, because screen-positive patients all had SCC at baseline. We did a post-hoc analysis of time to new additional systemic treatment, in which we compared randomised groups using Gray's test. For overall survival, we used Kaplan-Meier methods to estimate rates, and unadjusted and adjusted Cox regression models were fitted for intervention effect. Covariates used in the multivariate analysis were the same as for the cSCC models (prespecified analyses). For all non-patient-reported outcome analyses, p values of less than 0·05 were considered to be statistically significant.

We used the appropriate scoring manuals to calculate BPI pain, EORTC QLQ-C30, and HADS scores. We did cross-sectional analyses at each timepoint up to 24 months using the Mann-Whitney *U* test, with 12 months being the primary timepoint of interest. We assessed change from baseline to 12 months using ANCOVA, adjusting for the baseline scores. We used plots of residuals versus predicted values to assess the constant variance assumption. We did not consider data (including deaths) to be missing at random and therefore we explored patterns of missingness. In particular, completeness of data by visit period and baseline scores with and without paired 12-month scores were observed by randomised group for patient-reported outcomes to assess possible effects of missing data. To account for multiple testing of secondary patient-reported outcomes (including BPI), only p values of less than 0·01 were considered to be statistically significant.

We also present prespecified summary statistics relating to study conduct and procedures that could affect the study results, including number of MRIs done at 1 year and 2 years, use of spinal radiotherapy for rSCC, and subsequent systemic treatment.

Our analyses were based on a data cutoff of April 23, 2020, and were done using SAS (version 9.40), except for competing risk regression models, for which Stata (version 16) was used. The trial management group was overseen by an independent trial steering committee. Safety and efficacy data were reviewed regularly by an independent data monitoring committee. The trial was prospectively registered with ISRCTN, ISRCTN74112318.

### Role of the funding source

The funder provided peer-reviewed approval for the trial, but had no other role in study design, data collection, data analysis, data interpretation, or writing of the report.

## Results

**Figure 1:**
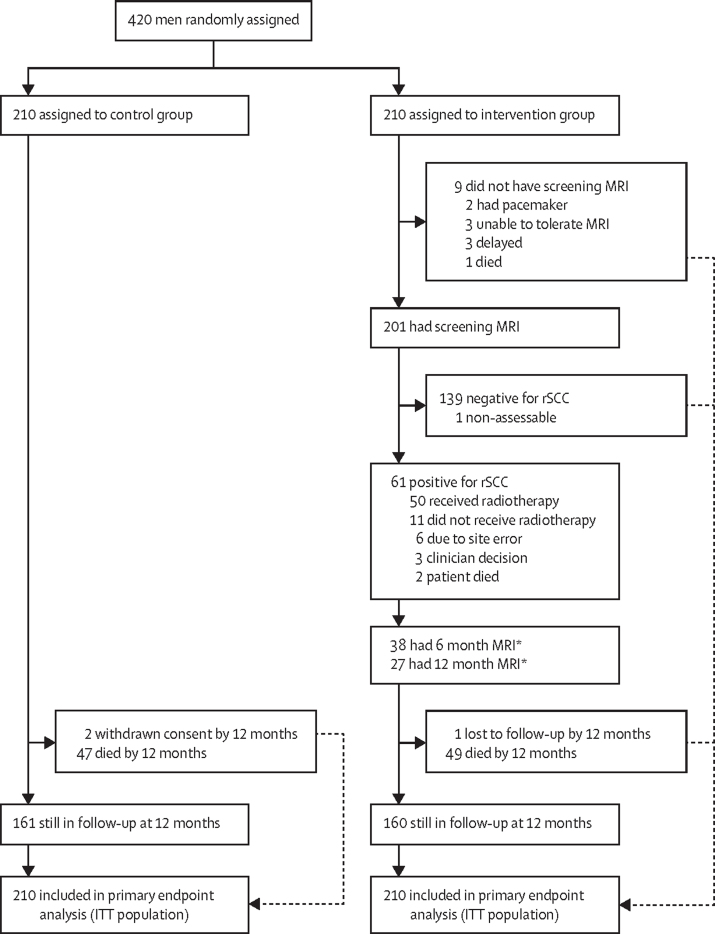
Trial profile Standard follow-up was at 3, 6, 9, 12, 15, 18, 21, 24, 30 and 36 months; status at 12 months is presented for illustrative purposes and was the primary timepoint of interest for assessments. ITT=intention-to-treat. rSCC=radiological spinal cord compression. *Some patients had both a 6 month and a 12 month MRI.

Between Feb 26, 2013, and April 25, 2017, 420 men were recruited and randomly assigned to the control (n=210) or intervention (n=210) group (figure 1; appendix p 5). Median age was 74 years (IQR 68–79), 222 (53%) of 420 patients had normal alkaline phosphatase concentrations, and median PSA concentration was 48 ng/mL (IQR 17–162; table 1; no data were collected on race or ethnicity). Clinical symptoms and signs recorded before randomisation were similar between assignment groups (appendix pp 6–7).

**Table 1:** Baseline characteristics

		**Control group (n=210)**	**Intervention group (n=210)**
Age at randomisation, years		74·3 (68·0–79·3)	74·2 (68·5–79·3)
Time from initial diagnosis to randomisation, years*		4·4 (2·4–8·0)	4·2 (2·4–7·5)
Time from CRPC diagnosis to randomisation, years		1·1 (0·3–2·7)	0·8 (0·3–1·7)
Primary tumour stage at diagnosis			
	T1–T2	45 (21%)	40 (19%)
	T3–T4	126 (60%)	134 (64%)
	TX	35 (17%)	30 (14%)
	Unknown	4 (2%)	6 (3%)
Metastatic disease at diagnosis		130 (62%)	124 (59%)
Biopsy at initial diagnosis		161 (77%)	170 (81%)
Gleason score at diagnosis†			
	≤6	15/161 (9%)	11/170 (6%)
	7	51/161 (32%)	50/170 (29%)
	≥8	86/161 (53%)	96/170 (56%)
	Unknown	9/161 (6%)	13/170 (8%)
Serum PSA, ng/mL‡		62 (20–187)	40 (15–120)
Alkaline phosphatase, U/L§		132 (93–248)	132 (88–226)
	Normal	111 (53%)	111 (53%)
	Raised	99 (47%)	99 (47%)
ECOG performance status			
	0	116 (55%)	116 (55%)
	1	85 (40%)	83 (40%)
	2	9 (4%)	11 (5%)
Sites of metastatic disease at randomisation			
	Bone	210 (100%)	209 (>99%)¶
	Lymph nodes	45 (21%)	39 (19%)
	Other	6 (3%)	6 (3%)
Treatments before randomisation			
	Prostatectomy	13 (6%)	17 (8%)
	Prostate radiotherapy	59 (28%)	55 (26%)
Initial first-line hormone treatment			
	LHRH analogues	174 (83%)	179 (85%)
	Anti-androgen monotherapy	18 (9%)	11 (5%)
	Maximal androgen blockade	14 (7%)	19 (9%)
	Orchidectomy	1 (<1%)	0
	Unknown	3 (1%)	1 (<1%)
Number of second-line systemic treatments§			
	0	7 (3%)	8 (4%)
	1	50 (24%)	38 (18%)
	2–3	83 (40%)	101 (48%)
	≥4	70 (33%)	63 (30%)
Treatments before randomisation			
	Second-generation endocrine therapy‖	93 (44%)	87 (41%)
	Chemotherapy	66 (31%)	57 (27%)
	Bone-protecting agent	13 (6%)	20 (10%)
	Radioisotope therapy	7 (3%)	6 (3%)
	Previous spinal radiotherapy or surgical procedure for metastatic disease§	16 (8%)	14 (7%)
Symptoms (CTCAE) at randomisation**			
	Back pain (all grade 1–2)	31 (15%)	42 (20%)
	Urinary incontinence (grade 1–3)	9 (4%)	16 (8%)
	Urinary retention (grade 1–3)	8 (4%)	11 (5%)
	Ataxia (all grade 1)	1 (<1%)	2 (1%)
	Paraesthesia (all grade 1–2)	10 (5%)	8 (4%)
	Degenerative spinal and neuromuscular disorders	20 (10%)	20 (10%)
	Previous spinal surgery for non-malignant disease	3 (1%)	1 (<1%)
	CT or PET-CT scan of trunk within 6 months of randomisation§††	64 (30%)	62 (30%)

Data are n (%) or median (IQR). CRPC=castration-resistant prostate cancer. PSA=prostate specific antigen. LHRH=luteinising hormone-releasing hormone analogue. ECOG=Eastern Co-operative Oncology Group. CTCAE=Common terminology criteria for adverse events.

* One control group patient had data missing.

† Denominator is number with biopsy at diagnosis.

‡ Within 3 weeks of randomisation.

§ Balancing factor at randomisation.

¶ One patient had no demonstrable bone metastases.

‖ Abiraterone or enzalutamide.

** Events by grade are in the appendix (pp 6–7).

†† Permitted by protocol amendment (approved on April 8, 2015)

On Feb 10, 2015, a formal pre-planned interim analysis, after 54 patients in the intervention group had their baseline MRI, confirmed the interim pre-stipulated rSCC rate was 10% or higher and recruitment continued.

At the data cutoff (April 23, 2020), median follow-up (according to reverse Kaplan-Meier) was 22 months (IQR 13–31).

201 (96%) of 210 patients in the intervention group had a screening spinal MRI (figure 1) with a median time from randomisation to scan of 30 days (IQR 15–35). No serious adverse events were reported within 24 h of screening MRI. 61 (31%) of 200 patients with assessable scans had rSCC and 140 individual metastases associated with rSCC were identified (median one lesion [IQR 1–3] per patient). Maximum ESCC scores were 1a: 26 (43%) of 61 patients; 1b: 17 (28%); 1c: 12 (20%); 2: two (3%), and 3: four (7%). 16 (11%) of 140 metastases were in the cervical spine, 41 (29%) in the upper thoracic spine (T1–T6), 50 (36%) in the lower thoracic spine (T7–T12), and 33 (24%) in the lumbar spine (table 2). Central review of MRIs was completed in September, 2018, and showed concordance of 92·4% with local radiology assessments (table 2). This estimate was based on agreement in ESCC score in 1904 sites out of 2060 sites reviewed.

**Table 2:** Local radiology assessment of sites of rSCC and comparison of local and centrally reviewed radiology assessments of ESCC scores from screening MRI scans in the intervention group

		**Score of 9**	**Score of 0**	**Score of 1a**	**Score of 1b**	**Score of 1c**	**Score of 2**	**Score of 3**	**Total**
**Local radiology assessment of ESCC scores for individual vertebra in screen-positive patients with rSCC**									
Any ESCC score 1a–3*									
	Cervical spine C1–C7	236	175	4 (3%)	9 (6%)	2 (1%)	1 (1%)	0	16 (11%)
	Thoracic spine upper T1–T6	133	192	24 (17%)	7 (5%)	5 (4%)	3 (2%)	2 (1%)	41 (29%)
	Thoracic spine lower T7–T12	120	196	30 (21%)	15 (11%)	3 (2%)	0	2 (1%)	50 (36%)
	Lumbar spine L1–L5	100	172	10 (7%)	16 (11%)	6 (4%)	0	1 (1%)	33 (24%)
	Total	589	735	68 (49%)	47 (34%)	16 (11%)	4 (3%)	5 (4%)	140 (100%)
Sites of maximum ESCC score†									
	Cervical spine C1–C7	0	0	0	4 (6%)	2 (3%)	1 (1%)	0	7 (11%)
	Thoracic spine upper T1–T6	0	0	11 (16%)	2 (3%)	4 (6%)	1 (1%)	2 (3%)	20 (30%)
	Thoracic Spine lower T7–T12	0	0	14 (21%)	7 (10%)	3 (4%)	0	1 (1%)	25 (37%)
	Lumbar spine L1–L5	0	0	6 (9%)	5 (7%)	3 (4%)	0	1 (1%)	15 (22%)
	Total	0	0	31 (46%)	18 (27%)	12 (18%)	2 (3%)	4 (6%)	67 (100%)
**Local assessment ESCC scores for patients included in central review**									
Central review ESCC score for individual vetebra‡									
	9	867 (42·1%)§	57 (2·8%)	1 (<0·1%)	0	0	0	0	925 (44·9%)
	0	44 (2·1%)	947 (46%)§	17 (0·8%)	5 (0·2%)	2 (0·1%)	0	0	1015 (49·3%)
	1a	0 (0%)	1 (0·1%)	48 (2·3%)§	5 (0·2%)	0	1 (<0·1%)	0	60 (2·9%)
	1b	2 (0·1%)	4 (0·2%)	1 (<0·1%)	32 (1·6%)§	4 (0·2%)	0	0	39 (1·9%)
	1c	1 (0·1%)	1 (0·1%)	0	4 (0·2%)	4 (0·2%)§	0	0	12 (0·6%)
	2	0	3 (0·2%)	0	0	1 (<0·1%)	3 (0·1%)§	2 (0·1%)	6 (0·3%)
	3	0	0	0	0	0	0	3 (0·1%)§	3 (0·1%)
	Total	914 (44·4%)	1013 (49·2%)	67 (3·3%)	46 (2·2%)	11 (0·5%)	4 (0·2%)	5 (0·2%)	2060 (100%)

Data are detected metastases. The ESCC scoring system (based on the Bilsky score system) is as follows: 9 indicates no bone metastasis (an additional score for PROMPTS trial); 0 indicates metastatic bone disease without epidural impingement; 1a indicates epidural impingement without deformation of the thecal sac; 1b indicates deformation of the thecal sac; 1c indicates deformation of the thecal sac with spinal cord abutment, but without cord compression; 2 indicates spinal cord compression but with CSF visible around the cord; 3 indicates spinal cord compression, with no CSF visible around the cord. ESCC=epidural spinal cord compression. rSCC=radiological spinal cord compression.

* Denominator for percentages is 140 vertebrae with rSCC, from 61 patients with rSCC at screening MRI.

† Denominator for percentages is 67 sites with maximum ESCC scores from 61 patients, five of whom had multiple sites of rSCC with their maximum ESCC score in more than one spinal region.

‡ Denominator for percentages is 2060 sites reviewed centrally from 58 rSCC screen-positive patients and 29 screen-negative patients (for local review, one patient had no ESCC score for 23 of 24 vertebrae; for central review, one patient had no ESCC score for five of 24 vertebra).

§ Agreement between interpretation of MRI scans comparing central review and local assessments.

Radiotherapy was given to sites of rSCC in 50 (82%) of 61 screen-positive patients (figure 1), with patients receiving a dose of 20 Gy in five fractions for 52 (91%) of 57 treatment sites (appendix p 7). Adverse events were uncommon after spinal radiotherapy for rSCC (appendix p 8). Grade 1–2 events occurring in at least 10% of patients were constipation (eight [16%] of 50 patients) and back pain (seven [14%]); one (2%) patient had a grade 3 adverse event of chest pain. Corticosteroids were given to 28 (46%) of 61 patients (median dose of dexamethasone was 8 mg [IQR 4–16], and the median duration of treatment was 11 days [IQR 7–19]).

**Figure 2:**
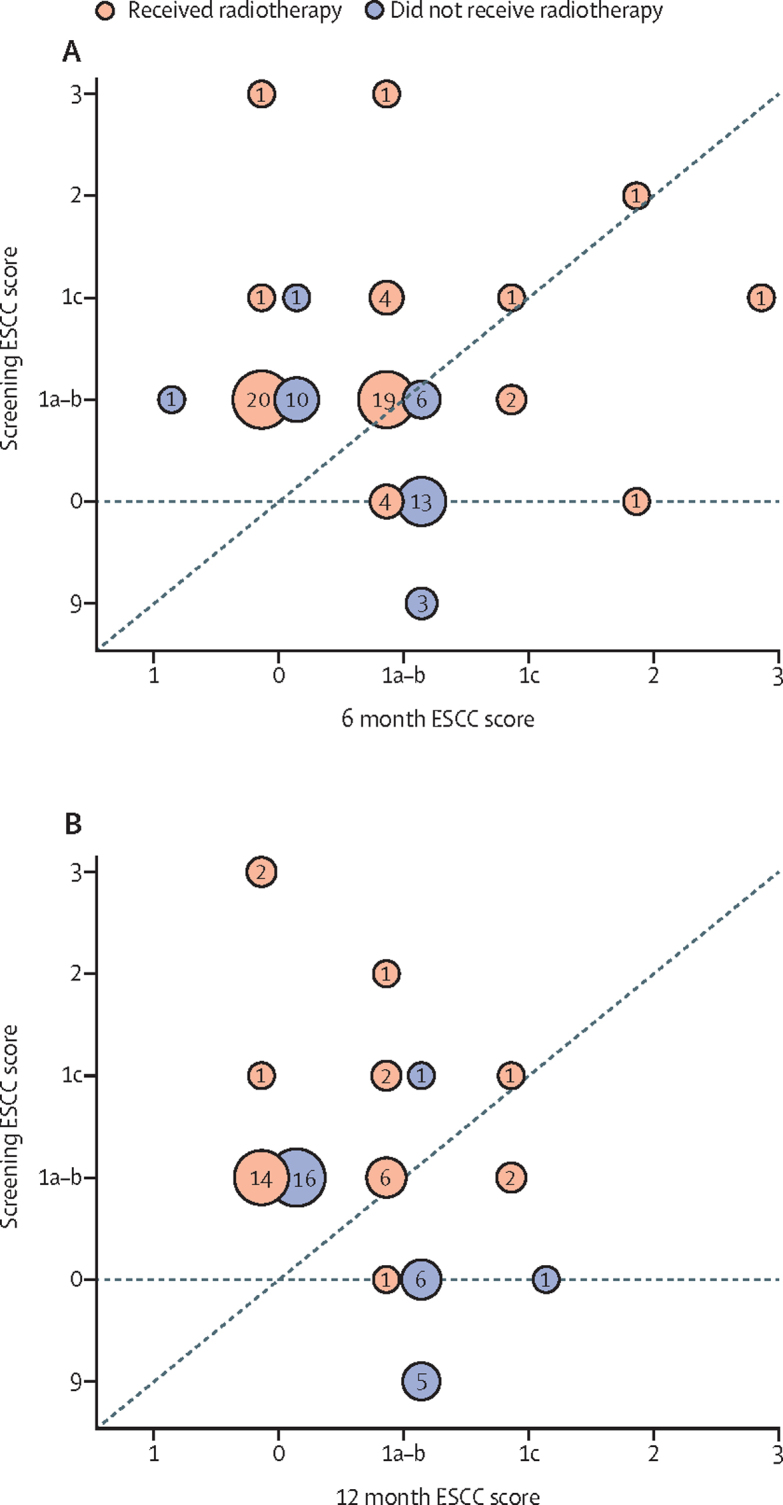
ESCC vertebra levels scores at screening and follow-up spinal MRI scans at 6 months (A) and 12 months (B) in patients in the intervention group with rSCC on screening MRI, managed with or without spinal radiotherapy Circles show the number of patients who did and did not receive radiotherapy. Circles on the diagonal dashed line from the origin had no change in ESCC score from baseline screening, circles above the line had an improvement in maximum ESCC score and those below the line had a deterioration in ESCC score. Radiotherapy was given to vertebra adjacent to sites of rSCC so that ESCC scores of 0 and 9 could increase to 1a or higher on follow-up. The ESCC scoring system (Bilsky score) is as follows: 9 indicates no bone metastasis (an additional score for the PROMPTS trial); 0 indicates metastatic bone disease without epidural impingement; 1a indicates epidural impingement without deformation of the thecal sac; 1b indicates deformation of the thecal sac; 1c indicates deformation of the thecal sac with spinal cord abutment, but without cord compression; 2 indicates spinal cord compression but with CSF visible around the cord; 3 indicates spinal cord compression, with no CSF visible around the cord. ESCC=epidural spinal cord compression.

Protocol-defined follow-up MRI was done in 32 (73%) of 44 screen-positive patients treated with radiotherapy and alive at 6 months. In these 32 evaluable patients, 39 (57%) of 69 assessable and treated metastases with rSCC had improved ESCC scores, 27 (39%) were stable, and three (4%) had progressed (two metastases from 1a–b to 1c and one metastasis from 1c to 3; figure 2; appendix p 9). However, in addition, 21 new sites of rSCC were seen in eight patients (figure 2 appendix p 9). At 12 months, in 21 evaluable patients (58% of 36 patients alive at 12 months), 37 (80%) of 46 assessable and treated sites with rSCC had improved ESCC scores, seven (15%) were stable, and two (4%) had progressed (two metastases from 1a–b to 1c; figure 2). A clinical decision was made not to treat 18 sites in four patients with rSCC (all ESCC score 1a–b or 1c); 12 (67%) of 18 sites had an improved ESCC score and six (33%) were stable, based on 6-month MRI assessment, and none had progressed by 12 months (figure 2).

On univariable analysis, covariates associated with rSCC were raised ALP (odds ratio [OR] 2·31 [95% CI 1·24–4·28]; p=0·0080) and ln PSA at randomisation (OR 1·50 [95% CI 1·19–1·89]; p=0·0006). Ln PSA at randomisation remained significantly associated with rSCC on multivariable analysis (OR 1·49 [1·15–1·92]; p=0·0023; appendix p 10). However, neither parameter could be used to separate clinically meaningful groups (appendix p 11).

**Figure 3:**
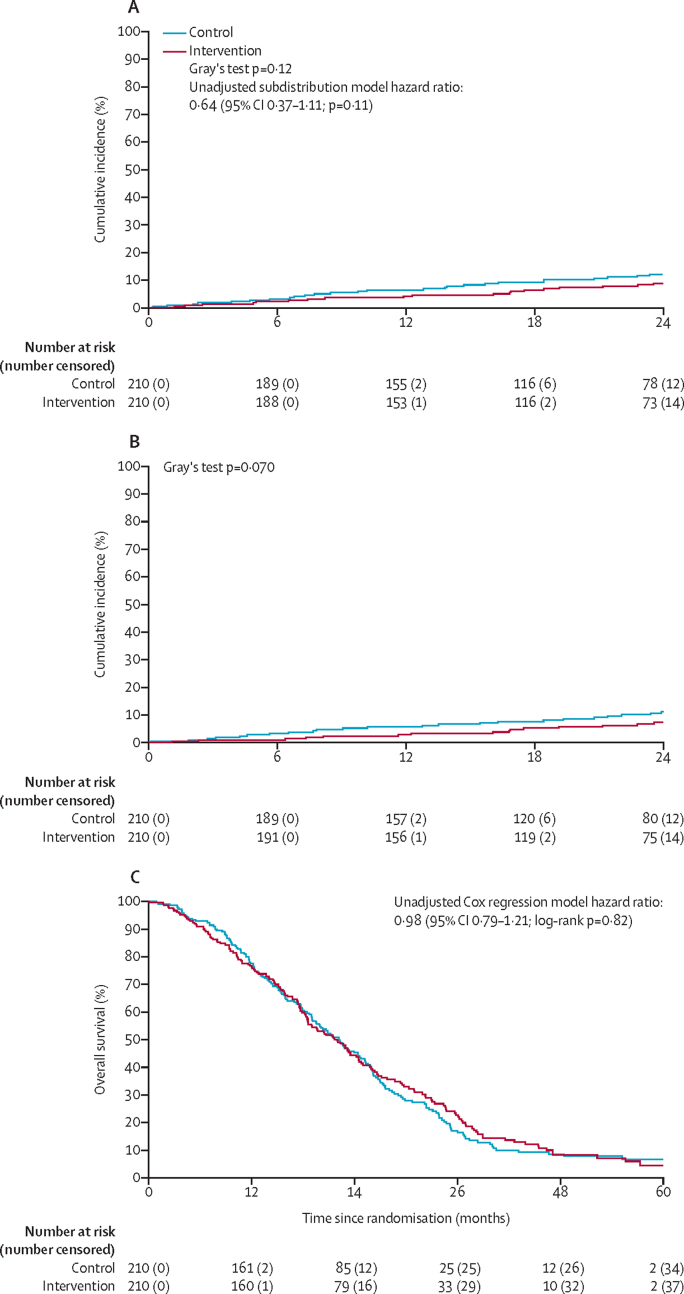
Cumulative incidence of cSCC (primary outcome; A) and persistent neurological functional deficit (B), and Kaplan-Meier plot of overall survival (C) cSCC=clinical spinal cord compression.

In the control group, the cumulative incidence of cSCC at 12 months was 6·7% (95% CI 3·8 to 10·6; 14 of 210) and at 24 months was 12·6% (8·5 to 17·5; 26 of 210) and for the intervention group at 12 months was 4·3% (2·1 to 7·7; nine of 210) and at 24 months was 9·2% (5·8 to 13·7; 19 of 210; Gray's test p=0·12; figure 3A). The difference at 12 months was −2·4% (95% CI −4·2 to 0·1). Median time to cSCC was not reached in either group. Unadjusted and adjusted subdistribution models showed no significant intervention effect (unadjusted HR 0·64 [95% CI 0·37 to 1·11; p=0·11]; and adjusted HR 0·62 [0·34 to 1·09; p=0·10]; appendix p 13). HRs for the development of cSCC calculated using a cause-specific model with death as a competing risk were similar (unadjusted HR 0·67 [0·38 to 1·16; p=0·15]; adjusted HR 0·61 [0·35 to 1·08; p=0·088]). Subdistribution and cause-specific models for death with cSCC as competing risk showed no intervention effect (appendix p 13). At 12 months, in the intervention group, those who were screen-positive for rSCC had a higher cumulative incidence of cSCC than those who were screen-negative for rSCC: seven (11·5% [95% CI 5·0 to 21·0]) of 61 patients versus two (1·3% [0·2 to 4·4]) of 139 patients. At 24 months, the cumulative incidence of cSCC increased to 13·2% (95% CI 6·1 to 23·1; eight of 61) in the screen-positive group and 7·6% (4·0 to 12·6; 11 of 139) in the screen-negative group (Gray's test p=0·13). The incidence of cSCC during the course of the study was lower in the rSCC screen-negative group than in the control group (Gray's test p=0·042; appendix p 14). In the control group, the cumulative incidence of SCC (rSCC or cSCC) at 6 months was 7·6% (95% CI 4·5 to 11·7; post hoc) and at 12 months 13·4% (9·2 to 18·4), and in the intervention screen-positive group, cumulative incidence at 6 months was 2·7% (0·9 to 6·3; post hoc) and at 12 months was 8·7% (4·9 to 14·0; appendix p 14). Associations of covariables with the development of cSCC are shown in the appendix (pp 12–13).

ESCC scores for the first cSCC event recorded over the duration of the trial up to data cutoff were observed in similar proportions in the two study groups with six (19%) of 32 patients in the control group having an ESCC score of 1a–b, two (6%) having a score of 1c, and 17 (53%) having a score of 2–3 and two (10%) of 21 in the intervention group having a score of 1a–b, four (19%) having a score of 1c, and 12 (57%) having a score of 2–3 (ten patients had unknown scores: seven in the control group and three in the intervention group). Frankel scores showed most patients remained ambulant (score of D) at the time of cSCC diagnosis in both groups, with scores of A–B in one (3%) of 32 patients with cSCC, a score of C in six (19%) patients, and a score of D in 19 (59%) patients in the control group, and a score of A–B in two (10%) of 21 patients, a score of C in two (10%) patients, and a score of D in nine (43%) patients in the intervention group (14 patients had unknown scores: six in the control group and eight in the intervention group; appendix p 15).

All patients treated for cSCC had initial radiotherapy, and one patient in the intervention group had subsequent salvage surgery. A dose of 20 Gy in five fractions was used for 17 (47%) of 36 sites treated and a further 15 sites received 8 Gy in single fraction (appendix p 7).

39 patients (26 in the control group and 13 in the intervention group) had assessable Frankel scores at least 6 months after the initial cSCC diagnosis, and four (15%) of 26 patients in the control group recovered to Frankel score E (no deficit) compared with three (23%) of 13 in the intervention group (appendix p 15). Time to irreversible FND (Frankel score A–D) is shown in figure 3B, with cumulative incidences of 5·7% (95% CI 3·1–9·5; 12 of 210) in the control group and 2·9% (1·2–5·8; six of 210) in the intervention group at 12 months and 11·2% (7·3–16·0; 23 of 210) in the control group and 7·3% (4·3–11·4; 15 of 210) in the intervention group at 24 months (Gray's test p=0·070). Patient-reported outcomes showed no significant differences between the study groups in any measure (appendix pp 16–23). Pain, assessed using the BPI, did not significantly differ between the two study groups (appendix p 16).

Overall survival was similar in both study groups with median overall survival of 22·2 months (IQR 12·4–32·7; 95% CI 19·1–14·7) in the control group and 22·0 months (IQR 12·4–34·6; 95% CI 18·6–24·4) in the intervention group (HR 0·98 [95% CI 0·79–1·21]; p=0·82; figure 3C). As of data cutoff, there were 174 deaths in the control group and 172 in the intervention group. Deaths due to prostate cancer were documented in 158 (91%) of 174 patients who died in the control group and 150 (87%) of 172 patients who died in the intervention group (appendix p 23). On multivariable analysis, covariates associated with overall survival were ALP (HR 1·9 [95% CI 1·5–2·4]; p<0·0001), ECOG performance status (1·6 [1·2–1·9]; p=0·0001), and ln PSA (1·3 [1·2–1·5]; p<0·0001; data for other covariates are in the appendix [p 24]).

**Figure 4:**
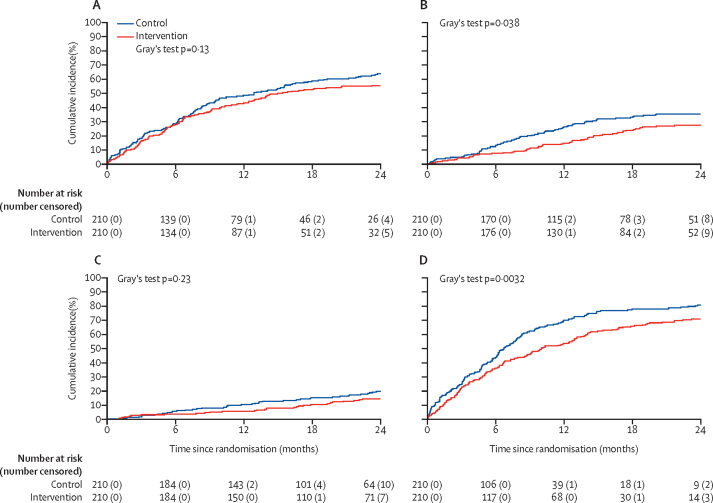
Post-hoc analysis of time to first additional post randomisation systemic anti-cancer treatment (A) New hormone therapy. (B) New chemotherapy. (C) New radioisotope therapy. (D) Any new systemic treatment. Death was treated as a competing risk.

New systemic treatments were started more commonly in the control group than in the intervention group, and significant differences were seen between the study groups for the cumulative incidence of starting chemotherapy and any new systemic treatment (post-hoc analysis; figure 4). At 12 months, the number of patients who had received chemotherapy was 55 (26%) of 210 in the control group and 31 (15%) of 210 in the intervention group and the number who started any new systemic treatment was 147 (70%) in the control group and 113 (54%) in the intervention group (appendix p 24). More spinal radiotherapy was used in the intervention than in the control group (appendix p 24).

Overall, 361 (201 screening, 85 protocol defined, and 74 additional) MRI scans for the intervention group and 98 spinal MRI scans for the control group were done in the 24 months after randomisation (appendix p 25).

## Discussion

To our knowledge, this is the first randomised controlled trial to assess the role of screening^20^ using spinal MRI to detect and treat rSCC in metastatic prostate cancer. We found no significant reduction in the proportion of patients with cSCC at 12 months, with a difference in the cumulative incidence between control and intervention groups of −2·4% (95% CI −4·2 to 0·1). We chose to use a validated ESCC scale, with minor modifications. This scale was developed by Bilsky and colleagues,^3^, ^6^, ^17^ for assessing rSCC on both screening and additional MRI scans. Although this scale is not routinely used in the UK, we found that specialist radiologists in the participating centres rapidly adapted to the scoring system with iterative feedback from the central review team.^21^ All vertebral levels were scored and levels of rSCC were reliably identified by local and central radiologists as suggested previously.^17^

We identified rSCC in 61 (31%) of 200 patients with assessable screening MRI scans. This proportion was similar to the 27–32% reported from previous single institution studies,^10^, ^11^ although higher than the incidence of 12·9% seen in asymptomatic patients. Any differences are probably due to patient selection factors varying between historical and contemporary cohorts and MRI reporting methods. 43 (70%) of 61 screened patients had ESCC grade 1a or b (ie, early rSCC), with a further 20% graded as 1c, and 10% graded 2 or greater. In the screened population, 82% of screen-positive patients went on to have pre-emptive treatment. Although consideration of surgical options^2^, ^3^, ^6^, ^22^ was encouraged in the protocol, treatment was uniformly done with radiotherapy. The protocol standard dose of 20 Gray in five fractions was used most commonly, which aligns with current practice for treatment of cSCC.^8^, ^23^ The effectiveness of radiotherapy is usually judged clinically according to ambulatory status. We had the additional opportunity to assess response radiologically, and repeat MRI after 6 months showed only three (4%) of 69 treated metastases had progressed in two patients*.* However, 21 new sites of rSCC had appeared in eight patients.

Despite the substantial incidence of rSCC, the development of cSCC in both study groups was lower than anticipated. Cumulative incidence of cSCC at 12 months, the primary endpoint of the trial, was 6·7% (95% CI 3·8–10·6) in the control group and 4·3% (2·1–7·7) in the intervention group. The rSCC screen-positive subgroup were at particular risk (11·5% [95% CI 5·0–21·0] cumulative incidence at 12 months) of the subsequent development of cSCC. In the MRI screen-negative group, the incidence of cSCC was very low at 1·3% (0·2–4·4) at 12 months, but then increased to 7·6% (4·0–12·6) at 24 months, which is in accordance with previous suggestions of a 12-month so-called protective window of a negative spinal MRI from single-centre studies.^10^, ^24^ A population-based study suggested a 7% prevalence of metastatic SCC in patients with metastatic castration-resistant prostate cancer,^9^ although single institution estimates have been as high as 24%.^7^, ^25^ Data from more recent trials using the new generation of life-prolonging therapies (eg, chemotherapies, new-generation hormonal treatments, and isotope therapy) have reported slightly lower incidences of cSCC (between 3% and 8%).^1^, ^26^, ^27^, ^28^, ^29^ Patients enrolled in the PROMPTS trial had biochemically progressing disease and additional systemic treatments were given as clinically appropriate. Effective systemic treatments reduce both the incidence of skeletal-related events, including cSCC,^1^, ^26^, ^29^ and are likely to have an effect on the progression of rSCC to cSCC. Assuming a similar but undetected rate of rSCC in the control group as in the intervention group, the rate of development of cSCC was considerably less than the rate of detection of rSCC. The reduction of use of systemic treatments in the intervention group in the 12 months after randomisation was unexpected, but a plausible mechanism might be through the effect of radiotherapy on progression in major sites of bone disease.^30^, ^31^ In particular, the use of radiotherapy to treat sites of oligoprogression, arising from resistant subclones, might allow the continuation rather than change of systemic treatments.^30^

Most patients with cSCC (28 [72%] of 39) in the trial remained ambulant after initial cSCC diagnosis, with Frankel scores of D or lower, with similar scores between the treatment groups. The incidence of FND seems to be lower than in past reports. Most patients with prostate cancer and SCC have previously been reported to be non-ambulatory;^4^, ^5^, ^32^ however, this disease-related morbidity has improved in more recent years, although most patients still have motor deficits.^3^, ^7^ We suspect the intended strict application of NICE guidelines for immediate assessment of new back pain^8^ and protocol-required 3-monthly follow-up for 2 years might have had a favourable effect in both treatment groups. Early detection of cSCC might encourage use of more contemporary treatment techniques to treat spinal metastasis that are more effective in achieving local control (eg, stereotactic body irradiation^3^, ^6^, ^31^, ^33^, ^34^). However, enthusiasm for early intervention after screening MRI should be tempered by the likelihood of overtreatment.

More imaging and radiotherapy resources were used in the intervention group than in the control group. This can be balanced against decreased use of new systemic treatments and possible reduction in FND in the intervention group. Refinement in the selection of patients for MRI screening would be helpful. In common with other investigators, we found that ECOG performance status, ALP, and PSA concentrations were related to survival.^7^ However, with the exception of ALP, we could not confirm previous observations that biochemical, clinical, or pathological parameters were risk factors for the development of cSCC.^3^, ^7^, ^33^ This finding might be because of the relatively small number of patients developing cSCC and the intervention for rSCC. We found that baseline PSA and ALP concentrations are related to the presence of rSCC, but neither covariate alone or in combination appeared to usefully stratify the patient population. We did not record data on extent of disease on technetium bone scan. Previous reports suggest that the number of spinal metastases or whole vertebral body involvement can be used to identify high-risk groups for the development of cSCC,^7^, ^10^, ^11^, ^25^ which might assist patient selection for screening MRI to detect clinically occult SCC.^10^, ^35^ It would be helpful to assess the association between extent of spinal disease on MRI and other imaging methods and rSCC and cSCC and also whether the extent of spinal disease at the time of first development of bone metastases or at the time of development of metastatic castration-resistant prostate cancer might assist in stratification of patients for screening MRI.

Limitations of this study include the non-blinded screening intervention allocation and the emphasis on patient and clinical staff appreciation of cSCC, which, although in line with NICE guidelines,^8^ might have led to earlier detection of cSCC than in usual clinical practice. With fewer events than expected, the study is likely to have been underpowered for the primary endpoint. Any assessment of the effect of radiotherapy treatment as an intervention is confounded by the use of additional systemic treatment options, but these are standard of care for metastatic castration-resistant prostate cancer and included life-prolonging options.^1^, ^14^, ^26^, ^28^, ^29^ A pragmatic decision was made to use the short Frankel instrument to assess FND in oncology clinics rather than more detailed neurological assessments. Data completeness decreased with duration of follow-up, as might be anticipated in an increasingly frail population, but was similar in the two study groups. A full cost-effectiveness analysis is outside the scope of this report. Such a report would need linkage to hospital episodes statistics data for robustness and ideally include a contemporary non-trial cohort with cSCC for comparison.

In summary, we found reproducibility of an ESCC scale and we recommend its widespread adoption in oncology practice.^6^ We found no significant differences in incidence of cSCC or irreversible FND between the MRI-screened intervention group and the control group. Severity of cSCC, judged by Frankel scores, were similar between the two groups, although scores were lower than in previous reports. MRI screen-detected early rSCC does not always progress to cSCC with contemporary systemic management of castration-resistant prostate cancer and observation might be sufficient for ESCC grade 1a–b rSCC. However, particular vigilance is recommended for these patients with a low threshold for recommending spinal MRI if any new back pain manifests because they are at substantial risk of developing new sites of cSCC. Further efforts to better identify patients at high risk for rSCC and cSCC are warranted to refine selection of groups for screening spinal MRI. The low rates of neurological impairment suggest that patients in both the intervention and control groups might have gained benefit from trial entry and emphasise the importance of the early detection and management of cSCC in line with NICE guidelines.^8^

## Data sharing

De-identified data will be made available to other researchers on request, subject to approval of a formal data access request in accordance with the ICR-CTSU data and sample access policy. Trial documentation, including the protocol, are available on request by contacting prompts-icrctsu@icr.ac.uk. The ICR-CTSU supports the wider dissemination of information from the research it does, and increased cooperation between investigators. Trial data are collected, managed, stored, shared, and archived according to ICR-CTSU Standard Operating Procedures to ensure the enduring quality, integrity, and utility of the data. Formal requests for data sharing are considered in line with the ICR-CTSU procedures with due regard given to funder and sponsor guidelines. Requests are via a standard proforma describing the nature of the proposed research and extent of data requirements. Data recipients are required to enter a formal data sharing agreement that describes the conditions for release and requirements for data transfer, storage, archiving, publication, and intellectual property. Requests are reviewed by the trial management group (TMG) in terms of scientific merit and ethical considerations including patient consent. Data sharing is allowed if proposed projects have a sound scientific or patient benefit rationale, as agreed by the TMG, and approved by the Trial Steering Committee as required. Restrictions relating to patient confidentiality and consent will be limited by aggregating and anonymising identifiable patient data. Additionally, all indirect identifiers that might lead to deductive disclosures will be removed in line with Cancer Research UK Data Sharing Guidelines. Additional documents might be shared if approved by the TMG and Trial Steering Committee (eg, statistical analysis plan and informed consent form).

**This online publication has been corrected. The corrected version first appeared at thelancet.com/oncology on March 28, 2022**

## Declaration of interests

EH reports grants from Cancer Research UK, during the conduct of the study and outside of the submitted work; grants and non-financial support from AstraZeneca and Bayer; and grants from Accuray, Varian Medical Systems, Janssen-Cilag, Roche Products, Merck Sharp & Dohme, and Pharma Limited (Sanofi). VH reports grants from Cancer Research UK, during the conduct of the study. GH reports speaker fees from Janssen outside the submitted work. CP reports speaker fees from Bayer and Janssen; advisory board fees from AAA, Clarity Pharmaceuticals, Myovant, ITM Radiopharma, and has an advisory board membership with Janssen and an education steering committee membership with Bayer, outside of the submitted work. DD reports grants from Cancer Research UK, during the conduct of the study; National Institute for Health Research (NIHR) Biomedical Research Centre (BRC) grants; and a patent (EP1933709B1, issued for a localisation and stabilisation device), outside of the submitted work. AS reports NIHR BRC funding to The Royal Marsden Hospital, speaker fees from Pfizer, and being past president and on the executive board for International Cancer Imaging Society (without payment), outside the submitted work. MM reports participation on Data Monitoring Board and Advisory Board for Endocyte and Clovis, outside the submitted work. All other authors declare no competing interests.
