# Correction to *Lancet Oncol* 2022; 23: 501–13

**DOI:** 10.1016/S1470-2045(22)00151-6

**Published:** 2022-04

*Dearnaley D, Hinder V, Hijab A, et al. Observation versus screening spinal MRI and pre-emptive treatment for spinal cord compression in patients with castration-resistant prostate cancer and spinal metastases in the UK (PROMPTS): an open-label, randomised, controlled, phase 3 trial.* Lancet Oncol *2022; **23:** 501–13*—In the Discussion of this Article, the second sentence of the third paragraph should have read “Cumulative incidence of cSCC at 12 months, the primary endpoint of the trial, was 6·7% (95% CI 3·8–10·6) in the control group and 4·3% (2·1–7·7) in the intervention group” and the second sentence of the fifth paragraph should have read “This can be balanced against decreased use of new systemic treatments…”. These corrections have been made to the online version as of March 28, 2022, and the printed version is correct.

