# Supplementary material for: Observation versus screening spinal MRI and pre-emptive treatment for spinal cord compression in patients with castration-resistant prostate cancer and spinal metastases in the UK (PROMPTS): an open-label, randomised, controlled, phase 3 trial
Source: Lancet Oncol. 2022 Apr;23(4):501–13. doi: 10.1016/S1470-2045(22)00092-4 (PMC8960282; doi:10.1016/S1470-2045(22)00092-4)
Supplement: Supplementary appendix [file mmc1.pdf]

THE LANCET
Oncology

Supplementary appendix

This appendix formed part of the original submission and has been peer reviewed. We post it as supplied by the authors.

Supplement to: Dearnaley D, Hinder V, Hijab A, et al. Observation versus screening spinal MRI and pre-emptive treatment for spinal cord compression in patients with castration-resistant prostate cancer and spinal metastases in the UK (PROMPTS): an open-label, randomised, controlled, phase 3 trial. *Lancet Oncol* 2022; published online March 10. https://doi.org/10.1016/S1470-2045(22)00092-4.

Results of a phase III randomised trial of observation versus screening spinal MRI and pre-emptive treatment for spinal cord compression in castrate resistant prostate cancer patients with spinal metastases: the PROMPTS trial.

Supplementary web-appendix

Contents

Appendix A Frankel Spinal Cord Injury Assessment Tool 3

Appendix B: ESCC score using modified Bilsky classification 4

Figure 1a. Prompts spinal MRI reporting atlas: Schematic representation of the 6-point ESCC grading scale. 4

Figure 1b. Prompts spinal MRI reporting atlas: Image atlas........ 4

Table 1. Final accrual by centre 5

Table 2. Signs and symptoms at baseline assessed by CTCAE v4.0 6

Table 3. Radiotherapy (RT) dose and fractionation used to treat rSCC and cSCC 7

Table 4. Assessment of adverse events using CTCAE v4.0 after adjuvant radiotherapy in the MRI screen +ve rSCC group 8

Table 5a. MRI re-assessment of patients with rSCC in the intervention group at 6 and 12 months after radiotherapy. Patient level data showing the number of patients with stable/improved MRI appearance or progression at original site of rSCC or development of new site(s) of r/cSCC. 9

Table 5b. MRI re-assessment of patients with rSCC in the intervention group at 6 and 12 months after radiotherapy. Assessment of individual spinal sites showing the number of spinal sites with stable/improved MRI appearance or progression at original site of rSCC or development of new site(s) of r/cSCC. 9

Table 6. Univariable and multivariable analysis of factors assessed for their relationship with the presence of rSCC on screening MRI in the intervention group 10

Figure 2a. Relationship of PSA and ALP to ESCC Scores on screening MRI scans........ 11

Figure 2b Relationship of ALP to development of cSSC in the control and intervention groups 12

Table 7. Assessment of MRI screening intervention: Estimated sub-distribution hazard and cause-specific hazard ratios for cSCC and competing risk event, death........ 13

Figure 3a. Cumulative incidence of first cSCC with MRI screen-negative patients and MRI screen-positive patients shown separately........ 14

Figure 3b. Cumulative incidence of first rSCC for in Control and MRI screen-negative intervention patients 14

Table 8a. Assessment of patients in control and intervention groups: ESCC scores at the time of first cSCC event 15

Table 8b. Assessment of patients in control and intervention groups: Frankel scores at the time of first cSCC event 15

Table 9. Assessment of patients in control and intervention groups groups: Relationship of initial Frankel score at the time of first cSCC with irreversible neurological deficit 15

Table 10. Patient reported outcomes summary statistics in control and intervention arm and 12 months analysis results........ 16

Figure 4. EORTC scales by treatment allocation and over visits........ 17

Figure 4. Continued EORTC........ 18

Figure 4. Continued EORTC........ 19

Figure 4. Continued EORTC........ 20

Figure 5. Brief Pain Index: Severity and interference by treatment allocation and over visits........ 21

Figure 6. HADS anxiety and depression scores by treatment allocation and over visits 22

Figure 7. EQ-5D-5L: Health State today by treatment allocation and over visits........ 23

Table 11. Causes of death in control and intervention groups ... 23
Table 12. Cox regression model for overall survival ... 24
Table 13. Types of additional systemic treatment received by patients in control and intervention group within 12 months of randomisation ... 24
Table 14. Spinal radiotherapy in control and intervention groups: Number of patients treated and radiotherapy courses delivered within 12 and 24 months of randomisation ... 24
Table 15. MRI in control and intervention groups: Number of protocol and clinically indicated scans performed within 24 months of randomisation ... 25
Protocol ... 26

Appendix A Frankel Spinal Cord Injury Assessment Tool

**Grade A**	Complete neurological injury - no motor or sensory function clinically detected below the level of the injury
**Grade B**	Preserved sensation only - no motor function clinically detected below the level of the injury; sensory function remains below the level of the injury but may include only partial function (sacral sparing qualifies as preserved sensation).
**Grade C**	Preserved motor non-functional - some motor function observed below the level of the injury, but is of no practical use to the patient.
**Grade D**	Preserved motor function - useful motor function below the level of the injury; patient can move lower limbs and walk with or without aid, but does not have a normal gait or strength in all motor groups.
**Grade E**	Normal motor - no clinically detected abnormality in motor or sensory function with normal sphincter function; abnormal reflexes and subjective sensory abnormalities may be present.

Appendix B: ESCC score using modified Bilsky classification

**0**	Metastatic bone disease without epidural impingement
**1a**	Epidural impingement without deformation of the thecal sac
**1b**	Deformation of the thecal sac
**1c**	Deformation of the thecal sac with spinal cord abutment, but without cord compression
**2**	Spinal cord compression but with cerebrospinal fluid (CSF) visible around the cord
**3**	Spinal cord compression, no CSF visible around the cord
**9**[1]	No bone metastasis (additional score for PROMPTS trial)

Figure 1a. Prompts spinal MRI reporting atlas: Schematic representation of the 6-point ESCC grading scale.

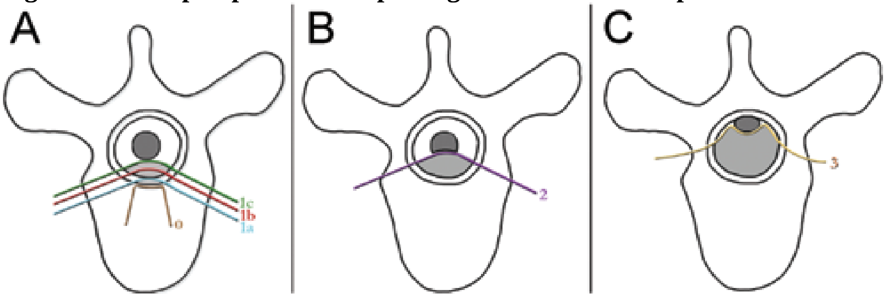

Diagrams are reproduced with the permission of the Journal of Neurosurgery Publishing Group.

1. An extra score of 9 indicates the absence of bone metastases in an individual vertebra and was added to the ESCC scoring system so that all vertebra could be assessed and scored.

Figure 1b. Prompts spinal MRI reporting atlas: Image atlas

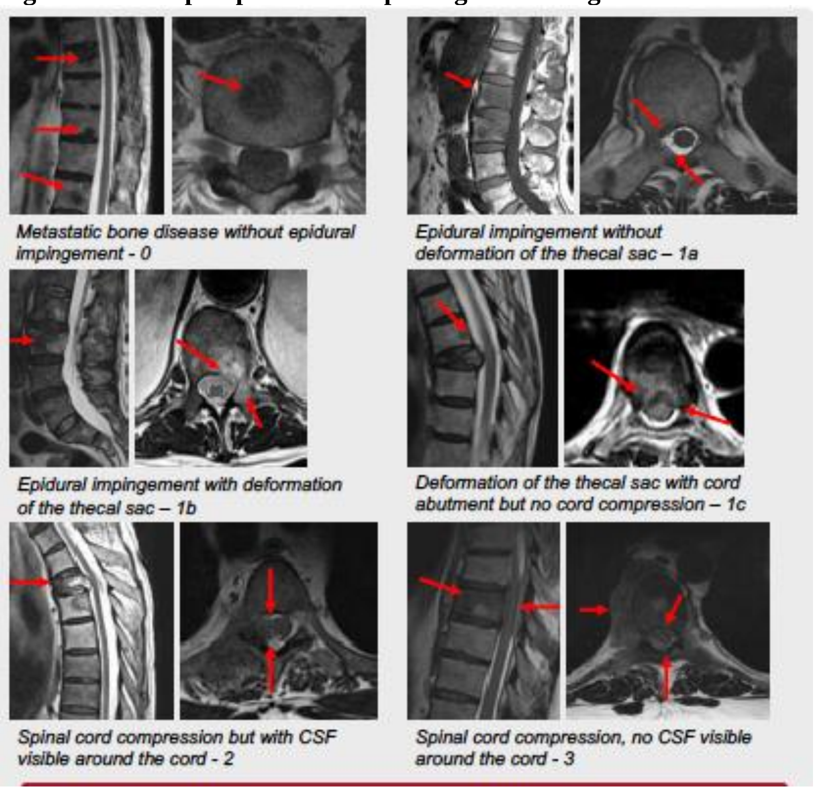

Table 1. Final accrual by centre

Centre	Principle investigator	Total	Centre	Principle investigator	Total
Queen Elizabeth Hospital, King's Lynn	Gail Horan	66	Mount Vernon Cancer Centre, Northwood	Peter Hoskin	6
Royal Shrewsbury Hospital	Narayanan Srihari	47	Royal Free Hospital, London	Maria Vilarino-Varela	6
St. James University Hospital, Leeds	Ann Henry	20	Mid Yorkshire Hospitals	Kanwarpal Gill	5
Royal Marsden Hospital, Sutton	David Dearnaley	19	Royal Surrey Hospital (Guildford)	Julian Money-Kyrle	5
Queen's Hospital, Romford	Stephanie Gibbs	18	Royal Devon & Exeter	Denise Sheehan	5
UCLH, London	Heather Payne	17	Medway Maritime Hospital	Henry Taylor	5
Freeman Hospital, Newcastle	Ian Pedley	17	Velindre Cancer Centre, Cardiff	Nachi Palniappan	5
Royal Bournemouth Hospital	Susannah Brock	14	Bradford Royal Infirmary	Ann Henry	4
Norfolk and Norwich University Hospital	Robert Wade	13	Western General Hospital, Edinburgh	Duncan McLaren	3
Royal Sussex County Hospital, Brighton	Angus Robinson	12	Sunderland Royal Infirmary	Ian Pedley	3
Ipswich Hospital, Ipswich	Ram Venkitaraman	11	West Suffolk Hospital	Yvonne Rimmer	2
Chesterfield and North Derbyshire Royal Hospital	Omar Din	11	Kingston Hospital	Nick Van As	2
James Paget University Hospital	Robert Wade	11	Weston General Hospital, Weston Super Mare	Serena Hilman	2
Maidstone General Hospital	Sharon Beesley	11	George Eliot Hospital, Nuneaton	Andrew Chan	2
Musgrove Park Hospital, Taunton	John Graham	10	Lister Hospital, Stevenage	Rob Hughes	2
University Hospital Coventry & Warwickshire	Jane Wordling	10	Royal Marsden Hospital, London	Vincent Khoo	1
Glangwili General Hospital	Mau-Don Phan	9	Clatterbridge Cancer Centre, Wirral	Isabel Syndikus	1
Huddersfield Royal Infirmary	Uschi Hoffman	8	Weston Park Hospital, Sheffield	Omar Din	1
Airedale General Hospital	Simon Brown	7	Croydon University Hospital	Robert Huddart	1
Belfast City Hospital	Suneil Jain	7	Darent Valley Hospital	Amanda Clarke	1
Harrogate District Hospital	Joji Joseph	6	Worthing Hospital	Ashok Nikapota	1
Ninewells Hospital, Dundee	Graeme Houston	6	Doncaster Royal Infirmary	Maymoona Alzouebi	1
Poole General Hospital	Joseph Davies	6			

Table 2. Signs and symptoms at baseline assessed by CTCAE v4.0

Signs and symptoms pre-specified terms	Treatment allocation				Total	
	Control		Intervention			
	N=210	%	N=210	%	N=420	%
**Back pain**						
**Grade 0**	176	84	166	79	342	81
**Grade 1**	30	14	39	19	69	16
**Grade 2**	1	1	3	1	4	1
**Grade 3**	0	0	0	0	0	0
**Unknown**	3	1	2	1	5	1
**Abdominal pain**						
**Grade 0**	200	95	205	98	405	96
**Grade 1**	6	3	1	1	7	2
**Grade 2**	0	0	0	0	0	0
**Unknown**	4	2	4	2	8	2
**Constipation**						
**Grade 0**	182	87	169	81	351	84
**Grade 1**	20	10	30	14	50	12
**Grade 2**	4	2	7	3	11	3
**Grade 3**	0	0	0	0	0	0
**Unknown**	4	2	4	2	8	2
**Diarrhoea**						
**Grade 0**	196	93	191	91	387	92
**Grade 1**	8	4	16	8	24	6
**Grade 2**	1	1	0	0	1	<1
**Grade 3**	0	0	0	0	0	0
**Unknown**	5	2	3	1	8	2
**Urinary incontinence**						
**Grade 0**	197	94	186	89	383	91
**Grade 1**	6	3	8	4	14	3
**Grade 2**	2	1	5	2	7	2
**Grade 3**	1	1	3	1	4	1
**Unknown**	4	2	8	4	12	3
**Urinary retention**						
**Grade 0**	196	93	191	91	387	93
**Grade 1**	5	2	7	3	12	5
**Grade 2**	2	1	3	1	5	<1
**Grade 3**	2	1	1	1	3	0
**Unknown**	5	2	8	4	13	2
**Ataxia**						
**Grade 0**	203	97	206	98	409	97
**Grade 1**	1	1	2	1	3	1
**Grade 2**	0	0	0	0	0	0
**Unknown**	6	3	2	1	8	2
**Parasthesia**						
**Grade 0**	193	92	198	94	391	93
**Grade 1**	10	5	10	5	20	5
**Grade 2**	1	1	0	0	1	<1
**Grade 3**	0	0	0	0	0	0
**Unknown**	6	3	2	1	8	2

Table 2 Continued. Signs and symptoms at baseline assessed by CTCAE v4.0

Signs and symptoms Non pre-specified terms	Treatment allocation				Total	
	Control		Intervention			
	N=210	%	N=210	%	N=420	%
**Fatigue**						
**Grade 0**	200	95	201	96	401	96
**Grade 1**	3	1	2	1	5	1
**Grade 2**	2	1	2	1	4	1
**Unknown**	5	2	5	2	10	2
**Other bone pain**						
**Grade 0**	203	97	197	94	400	95
**Grade 1**	1	1	4	2	5	1
**Grade 2**	1	1	4	2	5	1
**Unknown**	5	2	5	2	10	2
**Hypertension**						
**Grade 0**	204	97	205	98	409	97
**Grade 1**	0	0	0	0	0	0
**Grade 2**	0	0	0	0	0	0
**Grade 3**	1	1	0	0	1	<1
**Unknown**	5	2	5	2	10	2

Table 3. Radiotherapy (RT) dose and fractionation used to treat rSCC and cSCC

cSCC status	RT in first 24 months	Treatment allocation			Total	RT for initial rSCC diagnosis
		Control	Intervention			
			rSCC +ve at screening			
			Yes	No		
		N	N	N		
**Yes**	**8Gy/1fr**	7	1	7	15	
	**8Gy/2fr**	0	1	1	2	
	**20Gy/5fr**	14	1	2	17	
	**Other**	2	0	0	2	
**No**	**8Gy/1fr**	7	3	1	11	3
	**20Gy/5fr**	16	14	11	41	52
	**20Gy/4fr**	1	0	0	1	1
	**Other**	1	2	2	5	1
Total		48[a]	22[b]	24[c]	94	57[d]

Note: Includes multiple episodes of RT per patients.
Number of episodes of RT for rSCC diagnosis: a: 6 patients received 2 episodes, b:1 patient received 2 episodes, c: 3 patients received 2 episodes and 1 patient received 3 episodes, d: 7 patients received 2 episodes.

Table 4. Assessment of adverse events using CTCAE v4.0 after adjuvant radiotherapy in the MRI screen +ve rSCC group

Adverse Event	Grade 1-2 (n=50)[1]	Grade 3 (n=50)[1]
Constipation	8 (16%)	0 (0%)
Back pain	7 (14%)	0 (0%)
Fatigue	4 (8%)	0 (0%)
Paraesthesia	4 (8%)	0 (0%)
Joint/muscle Pain	2 (4%)	0 (0%)
Chest Pain	0 (0%)	1 (2%)
Sore throat	1 (2%)	0 (0%)
Nausea and vomiting	1 (2%)	0 (0%)
Urinary retention	1 (2%)	0 (0%)
Reduced Appetite	1 (2%)	0 (0%)
Abdominal pain	2 (4%)	0 (0%)
Diarrhoea	1 (2%)	0 (0%)
Peripheral neuropathy	1 (2%)	0 (0%)

1. Number of patients that were rSCC screen +ve who received radiotherapy following screening MRI.

Table 5a. MRI re-assessment of patients with rSCC in the intervention group at 6 and 12 months after radiotherapy. Patient level data showing the number of patients with stable/improved MRI appearance or progression at original site of rSCC or development of new site(s) of r/cSCC.

		6 months			12 months		
		New lesion			New lesion		
		Yes	No	Total	Yes	No	Total
Progression	Yes	2	1	3	1	0	1
	No	8	21	29	4	16	20
	Total	10	22	32	5	16	21

Table 5b. MRI re-assessment of patients with rSCC in the intervention group at 6 and 12 months after radiotherapy. Assessment of individual spinal sites showing the number of spinal sites with stable/improved MRI appearance or progression at original site of rSCC or development of new site(s) of r/cSCC.

Screening ESCC Score	6 month ESCC score							12 month ESCC score						
	9	0	1a/1b	1c	2	3	Total	9	0	1a/1b	1c	2	3	Total
**9**	240	93	3	0	0	0	336	152	62	5	0	0	0	219
**0**	30	315	17	0	1	0	363	21	210	7	1	0	0	239
**1a/1b**	1	30	25	2	0	0	58	0	30	6	2	0	0	38
**1c**	0	2	4	1	0	1	8	0	1	3	1	0	0	5
**2**	0	0	0	0	1	0	1	0	0	1	0	0	0	1
**3**	0	1	1	0	0	0	2	0	2	0	0	0	0	2
**Total**	271	441	50	3	2	1	768	173	305	22	4	0	0	504

No disease (dark green)	
Improvement in disease (green)	New lesion (yellow)
No worsening of disease (light green)	Worsening of disease (orange)

Table 6. Univariable and multivariable analysis of factors assessed for their relationship with the presence of rSCC on screening MRI in the intervention group

Model - adjusted covariates	Level	rSCC screen-ve (n=139)		rSCC screen +ve (n=61)		Univariate analysis			Multivariate analysis All covariates included in model N=210		
		n or median	% or Q1-Q3	n or median	% or Q1-Q3	odds ratio	95% CI	P- value	odds ratio	95% CI	P- value
**ALP**	**Normal**	81	58.30	23	37.7						
	**Raised**	58	41.70	38	62.3	2.31	1.24-4.28	0.008	1.62	0.82-3.19	0.17
**No. Previous treatments**	**First line**	45	32.4	25	41						
	**2nd line or later**	94	67.6	36	59	0.70	0.37-1.28	0.24	0.63	0.32-1.26	0.19
**Use of previous spinal RT and/or surgery for metastatic disease**	**No**	96	69.1	44	72.1						
	**Yes**	43	30.9	17	27.9	0.86	0.44-1.68	0.66	0.94	0.45-1.96	0.87
**Previous CT scan to thorax and abdomen in last 6 months**	**No**	95	68.3	45	73.8						
	**Yes**	44	31.7	16	26.2	0.77	0.39-1.51	0.44	0.69	0.33-1.45	0.33
**Time since development of CRPC1**	**1 year increments**	0.78	0.31-1.79	0.91	0.31-1.94	1.00	0.86-1.16	0.98	1.04	0.89-1.21	0.65
**Performance Status**	**ECOG 0**	77	55.4	31	50.8						
	**ECOG 1&2**	62	44.6	30	49.2	1.20	0.66-2.20	0.55	1.50	0.76-2.94	0.24
**lnPSA at randomisation1**	**Unit rise lnPSA**	3.4	2.6-4.5	4.26	3.5-5.4	1.50	1.19-2.89	0.0006	1.49	1.15-1.92	0.0023
**(PSA at randomisation)**		(30.5)	(14.0-88.0)	(70.6)	(33.4-216.5)						

1. Continuous variables

Figure 2a. Relationship of PSA and ALP to ESCC Scores on screening MRI scans

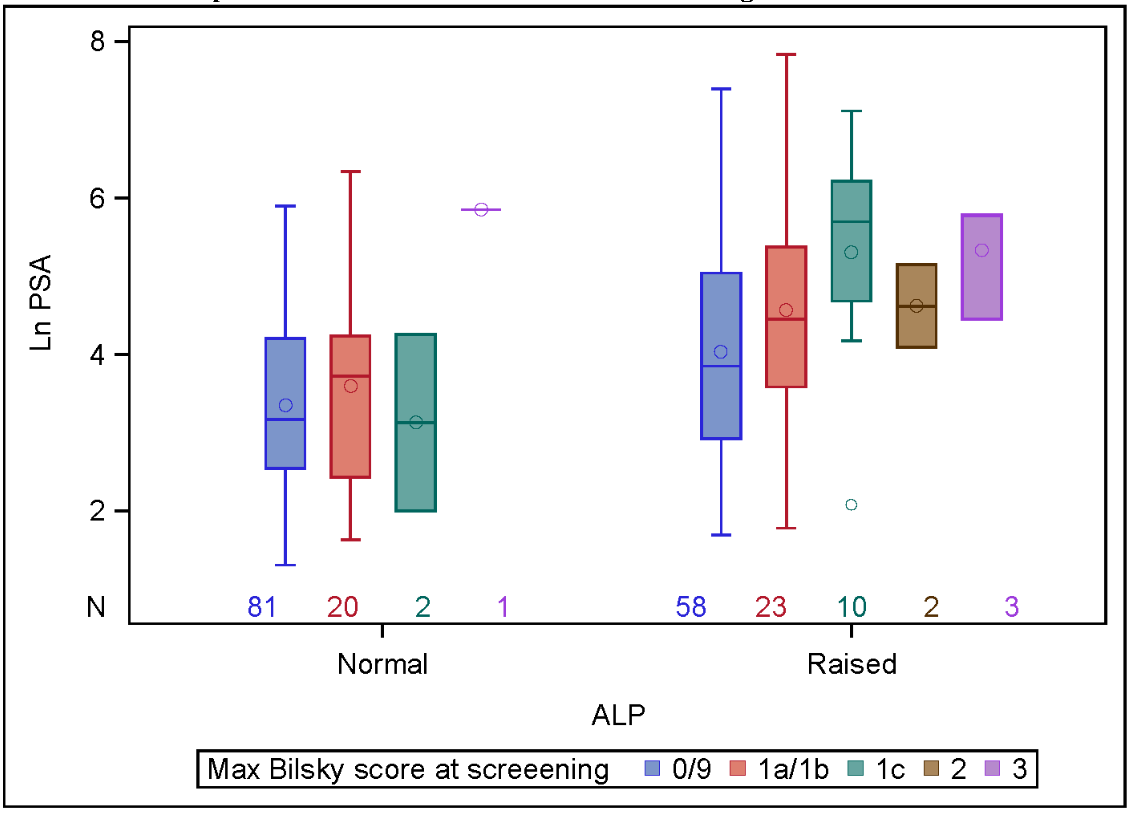

In the intervention group, for patients assessed for rSCC (n=200) using MRI, 96 patients in total had a raised ALP and 57 had a raised ALP and PSA above the median (47.8ng/ml). Of the 61 patients with any grade of rSCC at screening, 38 had raised ALP and 28 had a raised ALP and PSA above the median. Of the 18 rSCC positive patients at screening with ESCC scores of 1c, 2 or 3, 15 had raised ALP and 14 had a raised ALP and PSA above the median.

Figure 2b Relationship of ALP to development of cSSC in the control and intervention groups

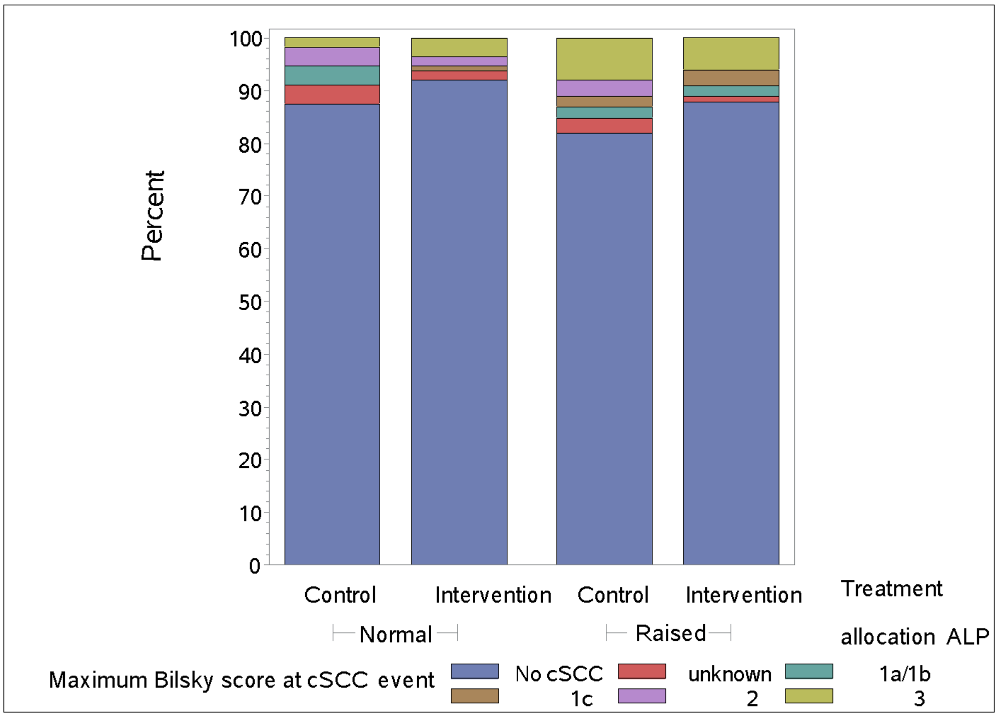

cSCC status and ESCC score a first cSCC event	Treatment allocation								Total	
	Control				Intervention					
	ALP				ALP					
	Normal		Raised		Normal		Raised			
	N	%	N	%	N	%	N	%	N	%
**No cSCC**	97	87	81	82	102	92	87	88	367	87
**Unknown ESCC score**	4	4	3	3	2	2	1	1	10	2
**1a/1b**	4	4	2	2	0	0	2	2	8	2
**1c**	0	0	2	2	1	1	3	3	6	1
**2**	4	4	3	3	2	2	0	0	9	2
**3**	2	2	8	8	4	4	6	6	20	5
**Total**	111	100	99	100	111	100	99	100	420	100

ESCC score at first cSCC event by 12 months follow up	Treatment allocation																			
	Control										Intervention									
	ALP								Total		ALP								Total	
	Normal				Raised						Normal				Raised					
	PSA low[1]		PSA high[1]		PSA low[1]		PSA high[1]				PSA low[1]		PSA high[1]		PSA low[1]		PSA high[1]			
	N	%	N	%	N	%	N	%	N	%	N	%	N	%	N	%	N	%	N	%
**1a/1b**	1	7	1	7	0	0	2	14	4	29	0	0	0	0	2	22	0	0	2	22
**1c**	0	0	0	0	0	0	1	7	1	7	1	11	0	0	2	22	1	11	4	44
**2**	0	0	1	7	1	7	0	0	2	14	0	0	1	11	0	0	0	0	1	11
**3**	1	7	0	0	2	14	4	29	7	50	0	0	1	11	0	0	1	11	2	22
**Total**	2	14	2	14	3	21	7	50	14	##	1	11	2	22	4	44	2	22	9	100

1. PSA below and above the median (47.8 ng/ml)

.

Raised ALP was the only variable found to have a significant association with the development of cSCC, but the number of events is small (appendix p 13)) and ALP groups did not adequately stratify patients for screening

Table 7. Assessment of MRI screening intervention: Estimated sub-distribution hazard and cause-specific hazard ratios for cSCC and competing risk event, death

			Sub-distribution model			Cause-specific Hazard		
			Hazard-ratio			Hazard-ratio		
**Event type**	**Model**	**Covariates (Comparison)**	**Estimate[1]**	**95% Confidence Intervals**	**p-Values**	**Estimate[1]**	**95% Confidence Intervals**	**p-Values**
**cSCC**	**Randomised group**	**Intervention vs Control**	0.64	0.37-1.11	0.110	0.67	0.38-1.16	0.149
	**Randomised group+**	**Intervention vs control**	0.62	0.34-1.09	0.10	0.61	0.35-1.08	0.088
	**ALP +**	**Raised vs Normal**			0.0040	2.14	1.22-3.80	0.0088
		**12 months[2]**	*2.07*	*0.34-12.52*	*0.0038[3]*			
		**24 months[2]**	*0.80*	*0.06-9.21*				
	**Previous systemic treatment+**	**2nd or later vs 1st line**	1.25	0.68-2.30	0.46	1.26	0.69-2.32	0.46
	**Previous spinal RT**	**Yes vs No**	0.64	0.34-1.20	0.17	0.64	0.34-1.21	0.17
	**CT within 6mths+**	**Yes vs No**	1.50	0.85-2.62	0.15	1.58	0.88-2.82	0.16
	**Time from CRPC[4]+**	**1 year increments**	0.99	0.86-1.12	0.83	0.98	0.876-1.11	0.75
	**ECOG+**	**EGOG 1&2 vs 0**	1.21	0.71-2.04	0.48	1.59	0.90-2.79	0.11
	**Ln PSA**	**Ln PSA (+1 unit)**	0.93	0.76-1.13	0.49	1.07	0.86-1.32	0.55
**Death**		**Intervention vs Control**	1.15	0.92-1.43	0.23	1.05	0.84-1.32	0.66
	**Treatment+**	**Intervention vs control**	1.25	0.99-1.57	0.055	1.07	0.84-1.35	0.60
	**ALP raised+**	**Raised vs Normal**			0.00010	1.93	1.52-2.46	<0.0001
		**12 months**	*1.91*	*0.89-4.08*	*0.0074[3]*			
		**24 months**	*1.35*	*0.49-3.72*				
	**Previous treatment+**	**2nd or later vs 1st line**	1.06	0.82-1.37	0.63	1.07	0.83-1.38	0.58
	**Previous spinal RT**	**Yes vs No**	0.96	0.74-1.25	0.80	0.92	0.71-1.20	0.55
	**CT within 6mths+**	**Yes vs No**	0.82	0.62-1.07	0.15	0.96	0.74-1.25	0.77
	**Time from CRPC[4]+**	**year rise=1**	1.01	0.96-1.05	0.73	1.00	0.95-1.05	1.00
	**ECOG+**	**EGOG 1&2 vs 0**			0.0078	1.57	1.24-1.99	0.00022
		**12 months[2]**	*1.48*	*0.72-3.04*	*0.041[3]*			
		**24 months[2]**	*1.14*	*0.30-1.34*				
	**Ln PSA**	**Ln PSA (+1 unit)**			<0.0001	1.28	1.17-1.40	<0.0001
		**12 months[2]**	*1.33*	*1.02-1.73*	*0.0065[3]*			
		**24 months[2]**	*1.13*	*0.45-1.09*				

1. HR <1 favours intervention
2. Time dependant variables estimates of HR at given time point
3. P-value for time dependant variable
4. Date of CRPC diagnosis is missing for one patient. The median time to CRPC was used for time since development of CRPC for this patient in order that this patient's data could be include in the model.

Figure 3a. Cumulative incidence of first cSCC with MRI screen-negative patients and MRI screen-positive patients shown separately

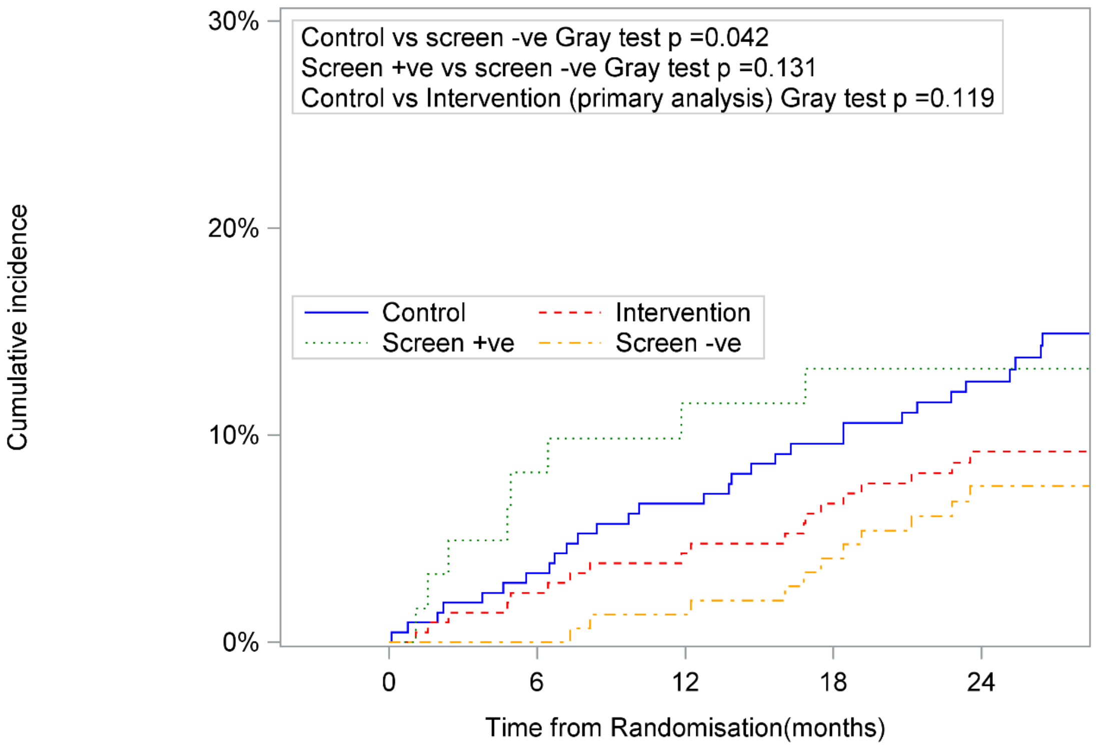

No. at risk(censored):

Control	210 (0)	189 (0)	155 (2)	116 (6)	78 (12)
Intervention	210 (0)	188 (0)	153 (1)	116 (2)	73 (14)
Screen +ve	61 (0)	48 (0)	31 (1)	22 (1)	12 (3)
Screen -ve	149 (0)	140 (0)	122 (0)	94 (1)	61 (11)

Figure 3b. Cumulative incidence of first SCC for in Control and MRI screen-negative intervention patients

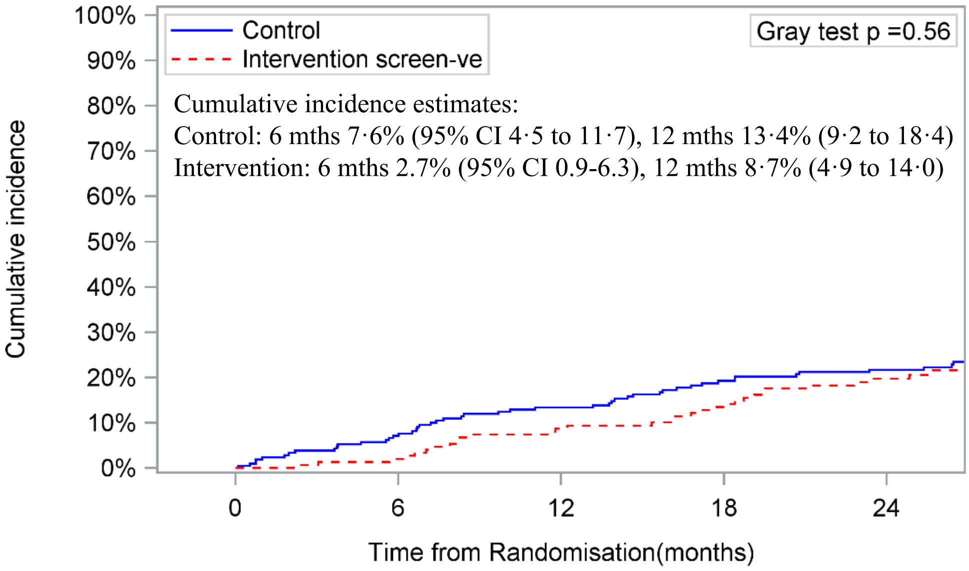

Number at risk (censored)

Control	210 (0)	182 (0)	144 (2)	102 (5)	70 (11)
Intervention screen-ve	149 (0)	138 (0)	115 (0)	83 (1)	53 (11)

Table 8a. Assessment of patients in control and intervention groups: ESCC scores at the time of first cSCC event

Maximum ESCC Score	Control		Intervention		Total	
	N	%	N	%	N	%
**1a**	1	3	0	0	1	2
**1b**	5	16	2	10	7	13
**1c**	2	6	4	19	6	11
**2**	7	22	2	10	9	17
**3**	10	31	10	48	20	38
**Unknown**	7	22	3	14	10	19
**Total**	32		21		53	

Table 8b. Assessment of patients in control and intervention groups: Frankel scores at the time of first cSCC event

Frankel Score	Control		Intervention		Total	
	N	%	N	%	N	%
**A**	1	3	1	5	2	4
**B**	0	0	1	5	1	2
**C**	6	19	2	10	8	15
**D**	19	60	9	43	28	53
**Not done**	6	19	8	38	14	26
**Total**	32		21		53	

Table 9. Assessment of patients in control and intervention groups groups: Relationship of initial Frankel score at the time of first cSCC with irreversible neurological deficit

Frankel Score	Control		Intervention		Total	
	Irreversible functional neurological deficit					
	Yes	No	Yes	No	Yes	No
**A**	1	0	1	0	2	0
**B**	0	0	1	0	1	0
**C**	5	1	2	0	7	1
**D**	16	3	6	3	22	6
**No neuro assess[1]**	6	0	7	1	13	1
**Total**	28	4	17	4	45	8

1. patients without neurological deficit measured during study but classified as cSCC

Table 10. Patient reported outcomes summary statistics in control and intervention arm and 12 months analysis results

	Control				Intervention				Mann-Whitney P-value	Change from baseline analysis		
	Baseline		12 months		Baseline		12 months					
	N	Median (IQR)	N	Median (IQR)	N	Median (IQR)	N	Median (IQR)		Mean difference (95% CI)	N (Control, intervention)	ANCOVA (p-value)
**EORTC QLQ C30**												
**Functional scales**												
**Physical functioning**	206	86.7 (73.3-93.3)	126	80.0 (60-86.7)	204	86.7 (66.7-93.3)	115	73.3 (53.3-86.7)	0.054	-4.6 (-9.4, 0.1)	239 (126, 113)	0.057
**Role functioning**	202	91.7 (66.7-100.0)	125	66.7 (50.0-100.0)	203	100 (66.7-100.0)	115	66.7 (33.3-100.0)	0.052	8.0 (-15.5,-0.4)	236 (123, 113)	0.039
**Emotional functioning**	205	91.7 (75.0-100.0)	125	91.7 (75.0-100.0)	204	91.7 (75.0-100.0)	115	91.7 (66.7-100.0)	0.83	-1.1 (-5.5, 3.3)	238 (125, 113)	0.62
**Cognitive functioning**	205	100.0 (83.3-100.0)	126	100.0 (66.7-100.0)	201	83.3 (83.3-100.0)	114	83.3 (66.7-100.0)	0.10	-0.8 (-5.1, 3.6)	236 (126, 110)	0.73
**Social functioning**	204	100.0 (66.7-100.0)	125	83.3 (66.7-100.0)	204	100.0 (66.7-100.0	113	83.3 (66.7-100.0)	0.58	-2.2 (-9.0, 4.7)	234 (123, 111)	0.53
**Global health QoL[1]**	206	75.0 (58.3-83.3)	126	75.0 (58.3-83.3)	204	75.0 (62.5-83.3)	115	66.7 (50.0-83.3)	0.052	-5.3 (-10.5, -0.3)	239 (126, 113)	0.049
**Pain[2]**	204	16.7 (0.0-33.3)	126	16.7 (16.7-33.3)	202	16.7 (0.0-33.3)	115	33.3 (16.7-50.0)	0.32	4.5 (-1.9, 10.9)	237 (125, 112)	0.16
**Brief pain index[2]**												
**Severity**	197	0.5 (0.0-2.0)	117	1.3 (0.0-3.8)	199	1.0 (0.0-2.8)	113	2.0 (0.0-4.3)	0.13	0.4 (-0.2, 0.9)	218 (111, 107)	0.21
**Interference**	196	0.0 (0.0-1.8)	118	1.1 (0.0-3.3)	198	0.3 (0.0-1.9)	113	1.9 (0.0-4.0)	0.15	0.4 (-0.2, 1.0)	216 (111, 105)	0.18
**HADS[2, 3]**												
**Anxiety**	184	3.5 (1.0-6.0)	157	3.0 (2.0-6.0)	191	3.0 (1.0-6.0)	142	3.0 (1.0-5.0)	0.18	-0.2 (-0.7, 0.4)	282 (145, 137)	0.57
**Depression**	184	3.0 (1.0-4.0)	157	3.0 (1.0-5.0)	191	3.0 (1.0-5.0)	142	3.0 (1.0-5.0)	0.18	0.1 (-0.4, 0.7)	282 (145, 137)	0.65
**EQ-5D-5L[1]**												
**Heath state today**	204	80.0 (70.0-90.0)	123	75.0 (60.0-85.0)	203	80.0 (65.0-90.0)	115	75.0 (50.0-85.0)	0.72	-1.5 (-5.7, 2.7)	233 (121, 112)	0.48

1. Higher score indicates better health
2. Higher score indicates worse health
3. HADS based on 3 months scores (collected at baseline and 3 months only)

Figure 4. EORTC scales by treatment allocation and over visits

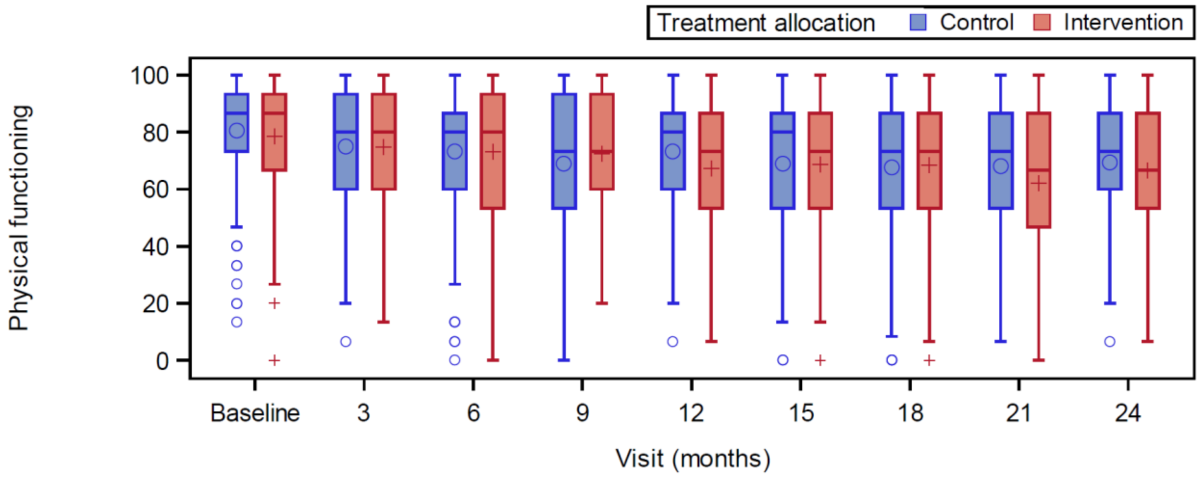

Statistics	Baseline	3	6	9	12	15	18	21	24
N (Control)	206	175	160	142	126	103	88	69	52
Median(C)	86.7	80.0	80.0	73.3	80.0	80.0	73.3	73.3	73.3
Q1(C)	73.3	60.0	60.0	53.3	60.0	53.3	53.3	53.3	60.0
Q3(C)	93.3	93.3	86.7	93.3	86.7	86.7	86.7	86.7	86.7
N (Intervention)	204	169	155	137	115	99	86	67	54
Median (I)	86.7	80.0	80.0	73.3	73.3	73.3	73.3	66.7	66.7
Q1(I)	66.7	60.0	53.3	60.0	53.3	53.3	53.3	46.7	53.3
Q3(I)	93.3	93.3	93.3	93.3	86.7	86.7	86.7	86.7	86.7
Mann-Whitney p-value	.	0.65	0.98	0.47	0.054	0.82	0.90	0.14	0.55

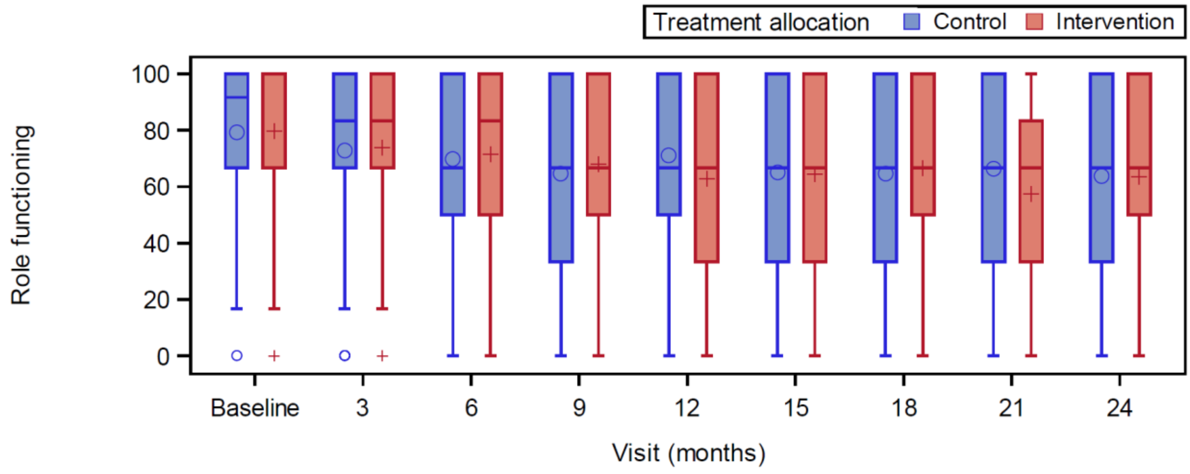

Statistics	Baseline	3	6	9	12	15	18	21	24
N (Control)	202	174	158	142	125	103	86	69	51
Median(C)	91.7	83.3	66.7	66.7	66.7	66.7	66.7	66.7	66.7
Q1(C)	66.7	66.7	50.0	33.3	50.0	33.3	33.3	33.3	33.3
Q3(C)	100.0	100.0	100.0	100.0	100.0	100.0	100.0	100.0	100.0
N (Intervention)	203	169	154	135	115	95	84	65	54
Median (I)	100.0	83.3	83.3	66.7	66.7	66.7	66.7	66.7	66.7
Q1(I)	66.7	66.7	50.0	50.0	33.3	33.3	50.0	33.3	50.0
Q3(I)	100.0	100.0	100.0	100.0	100.0	100.0	100.0	83.3	100.0
Mann-Whitney p-value		0.82	0.79	0.60	0.05	0.97	0.77	0.11	0.90

Figure 4. Continued EORTC scales by treatment allocation and over visits

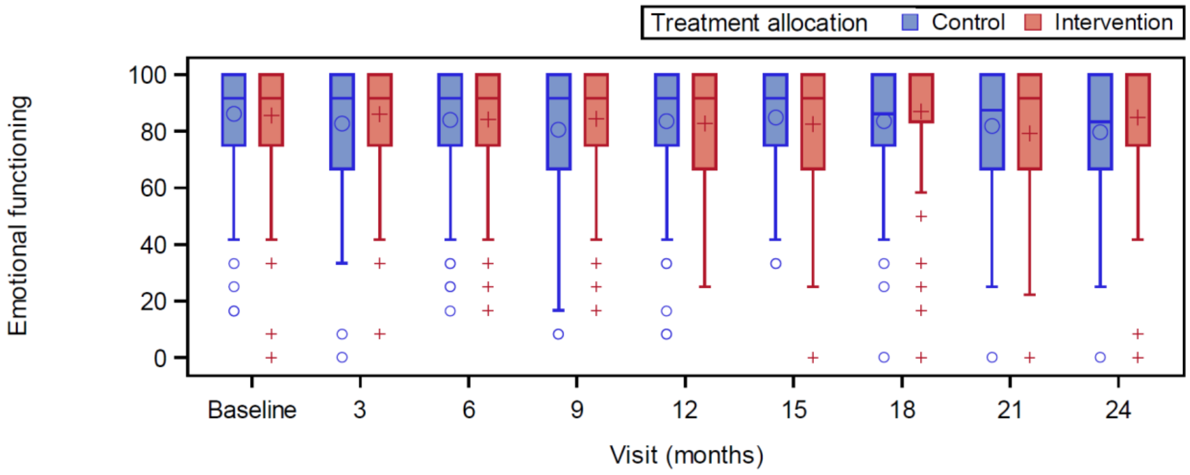

Statistics	Baseline	3	6	9	12	15	18	21	24
N (Control)	205	175	160	142	125	102	88	70	51
Median(C)	91.7	91.7	91.7	91.7	91.7	91.7	86.1	87.5	83.3
Q1(C)	75.0	66.7	75.0	66.7	75.0	75.0	75.0	66.7	66.7
Q3(C)	100.0	100.0	100.0	100.0	100.0	100.0	100.0	100.0	100.0
N (Intervention)	204	169	155	136	115	99	85	67	54
Median (I)	91.7	91.7	91.7	91.7	91.7	91.7	100.0	91.7	100.0
Q1(I)	75.0	75.0	75.0	75.0	66.7	66.7	83.3	66.7	75.0
Q3(I)	100.0	100.0	100.0	100.0	100.0	100.0	100.0	100.0	100.0
Mann-Whitney p-value	.	0.13	0.39	0.35	0.83	0.97	0.03	0.69	0.13

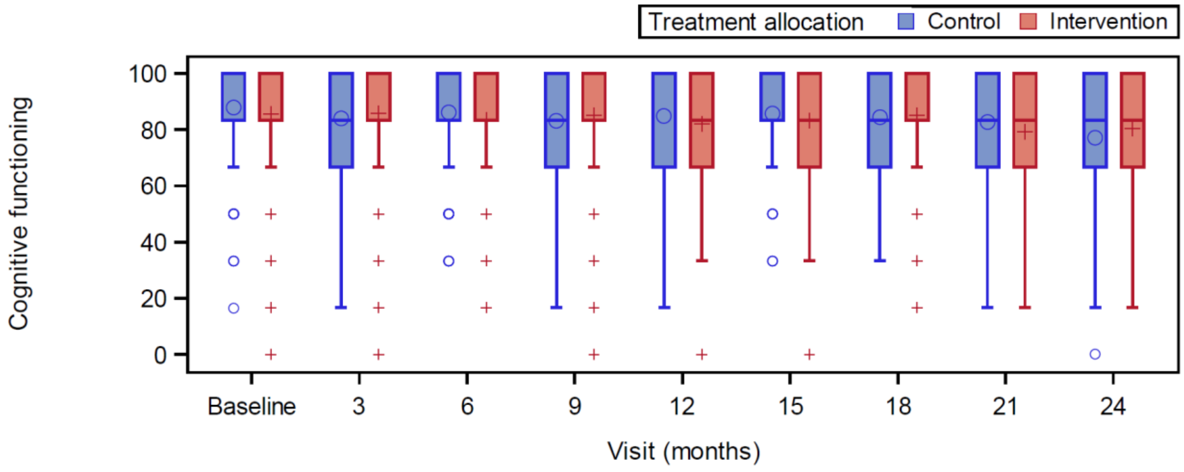

Statistics	Baseline	3	6	9	12	15	18	21	24
N (Control)	205	175	159	142	126	102	87	70	50
Median(C)	100.0	83.3	83.3	83.3	100.0	83.3	83.3	83.3	83.3
Q1(C)	83.3	66.7	83.3	66.7	66.7	83.3	66.7	66.7	66.7
Q3(C)	100.0	100.0	100.0	100.0	100.0	100.0	100.0	100.0	100.0
N (Intervention)	201	169	155	135	114	99	84	67	53
Median (I)	83.3	83.3	83.3	83.3	83.3	83.3	83.3	83.3	83.3
Q1(I)	83.3	83.3	83.3	83.3	66.7	66.7	83.3	66.7	66.7
Q3(I)	100.0	100.0	100.0	100.0	100.0	100.0	100.0	100.0	100.0
Mann-Whitney p-value	.	0.44	0.55	0.57	0.10	0.41	0.67	0.56	0.25

Figure 4. Continued EORTC scales by treatment allocation and over visits

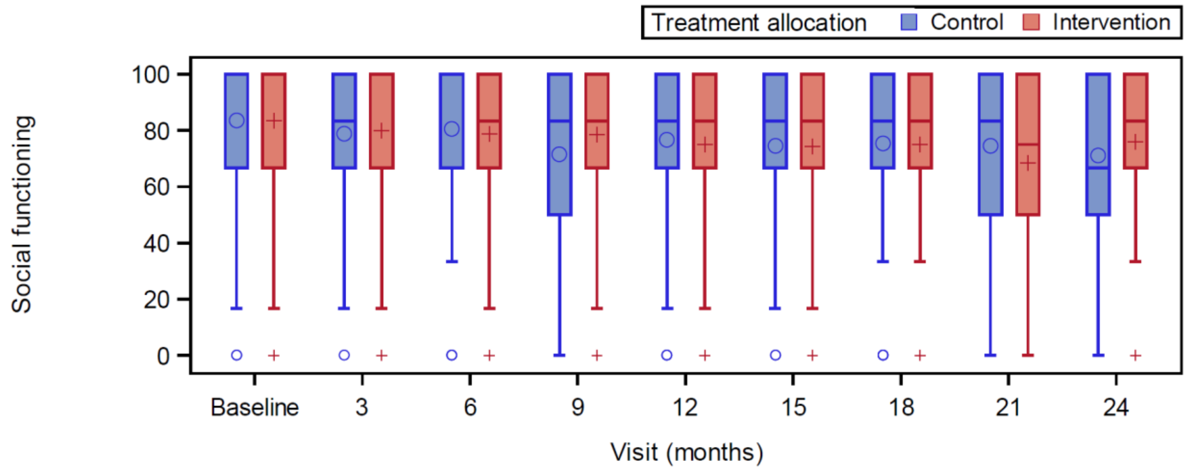

Statistics	Baseline	3	6	9	12	15	18	21	24
N (Control)	204	174	159	141	125	99	86	70	51
Median(C)	100.0	83.3	100.0	83.3	83.3	83.3	83.3	83.3	66.7
Q1(C)	66.7	66.7	66.7	50.0	66.7	66.7	66.7	50.0	50.0
Q3(C)	100.0	100.0	100.0	100.0	100.0	100.0	100.0	100.0	100.0
N (Intervention)	204	170	154	133	113	99	84	66	54
Median (I)	100.0	100.0	83.3	83.3	83.3	83.3	83.3	75.0	83.3
Q1(I)	66.7	66.7	66.7	66.7	66.7	66.7	66.7	50.0	66.7
Q3(I)	100.0	100.0	100.0	100.0	100.0	100.0	100.0	100.0	100.0
Mann-Whitney p-value	.	0.31	0.75	0.19	0.58	1.0	0.77	0.092	0.42

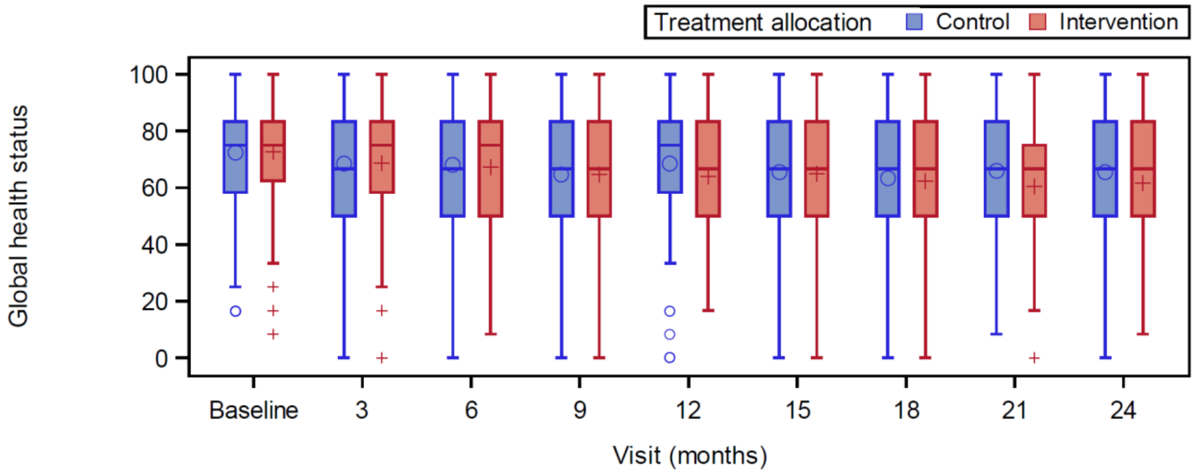

Statistics	Baseline	3	6	9	12	15	18	21	24
N (Control)	206	175	160	142	126	103	88	70	51
Median(C)	75.0	66.7	66.7	66.7	75.0	66.7	66.7	66.7	66.7
Q1(C)	58.3	50.0	50.0	50.0	58.3	50.0	50.0	50.0	50.0
Q3(C)	83.3	83.3	83.3	83.3	83.3	83.3	83.3	83.3	83.3
N (Intervention)	204	170	155	136	115	99	85	66	54
Median (I)	75.0	75.0	75.0	66.7	66.7	66.7	66.7	66.7	66.7
Q1(I)	62.5	58.3	50.0	50.0	50.0	50.0	50.0	50.0	50.0
Q3(I)	83.3	83.3	83.3	83.3	83.3	83.3	83.3	75.0	83.3
Mann-Whitney p-value	.	0.90	0.85	0.73	0.052	0.76	0.64	0.063	0.32

Figure 4. Continued EORTC scales by treatment allocation and over visits

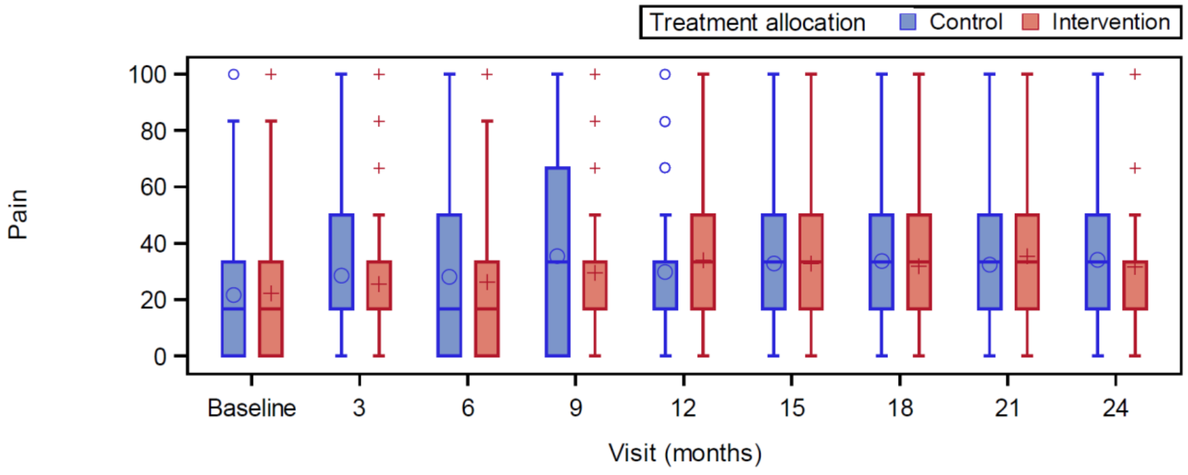

Statistics	Baseline	3	6	9	12	15	18	21	24
N (Control)	204	173	158	141	126	101	86	67	51
Median(C)	16.7	16.7	16.7	33.3	16.7	33.3	33.3	33.3	33.3
Q1(C)	0.0	16.7	0.0	0.0	16.7	16.7	16.7	16.7	16.7
Q3(C)	33.3	50.0	50.0	66.7	33.3	50.0	50.0	50.0	50.0
N (Intervention)	202	168	153	133	115	99	83	66	54
Median (I)	16.7	16.7	16.7	33.3	33.3	33.3	33.3	33.3	33.3
Q1(I)	0.0	16.7	0.0	16.7	16.7	16.7	16.7	16.7	16.7
Q3(I)	33.3	33.3	33.3	33.3	50.0	50.0	50.0	50.0	33.3
Mann-Whitney p-value	.	0.28	0.46	0.32	0.32	0.88	0.86	0.62	0.62

Figure 5. Brief Pain Index: Severity and interference by treatment allocation and over visits

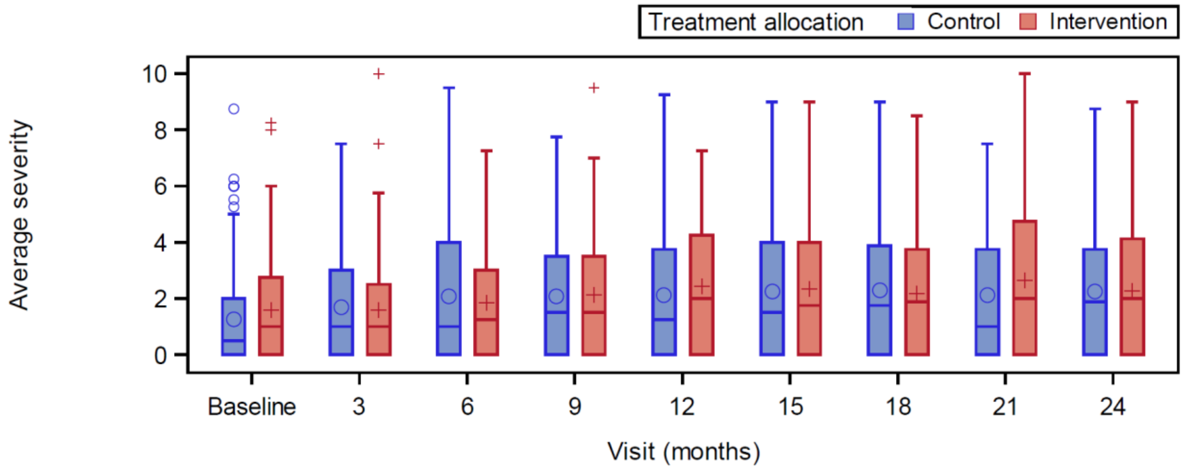

Statistics	Baseline	3	6	9	12	15	18	21	24
N (Control)	197	166	150	140	117	96	80	66	50
Median(C)	0.5	1.0	1.0	1.5	1.3	1.5	1.8	1.0	1.9
Q1(C)	0.0	0.0	0.0	0.0	0.0	0.0	0.0	0.0	0.0
Q3(C)	2.0	3.0	4.0	3.5	3.8	4.0	3.9	3.8	3.8
N (Intervention)	199	167	150	137	113	89	80	61	52
Median (I)	1.0	1.0	1.3	1.5	2.0	1.8	1.9	2.0	2.0
Q1(I)	0.0	0.0	0.0	0.0	0.0	0.0	0.0	0.0	0.0
Q3(I)	2.8	2.5	3.0	3.5	4.3	4.0	3.8	4.8	4.1
Mann-Whitney p-value	.	0.79	0.84	0.88	0.13	0.80	0.92	0.24	0.98

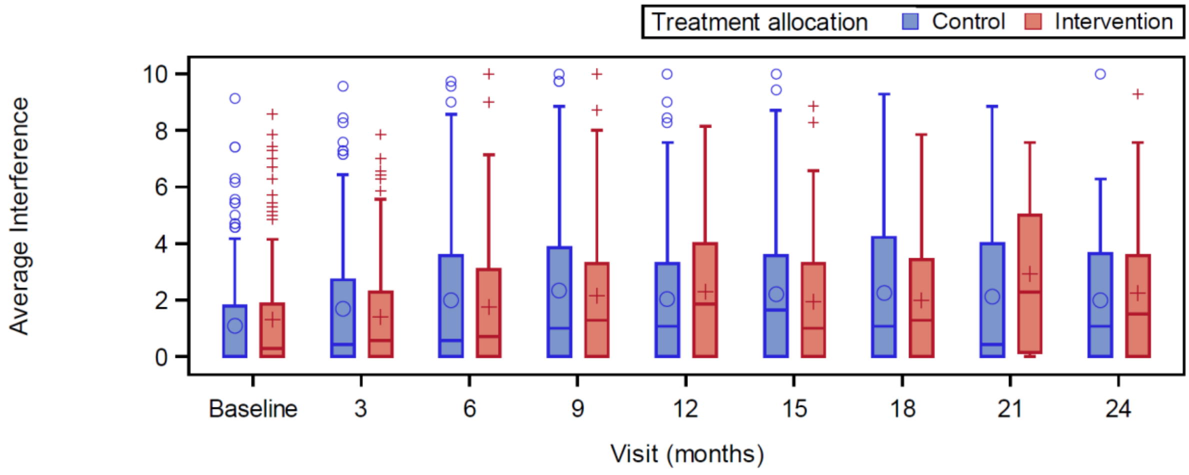

Statistics	Baseline	3	6	9	12	15	18	21	24
N (Control)	196	167	151	137	118	98	80	65	52
Median(C)	0.0	0.4	0.6	1.0	1.1	1.6	1.1	0.4	1.1
Q1(C)	0.0	0.0	0.0	0.0	0.0	0.0	0.0	0.0	0.0
Q3(C)	1.8	2.7	3.6	3.9	3.3	3.6	4.2	4.0	3.6
N (Intervention)	198	163	152	135	113	90	84	62	50
Median (I)	0.3	0.6	0.7	1.3	1.9	1.0	1.3	2.3	1.5
Q1(I)	0.0	0.0	0.0	0.0	0.0	0.0	0.0	0.1	0.0
Q3(I)	1.9	2.3	3.1	3.3	4.0	3.3	3.4	5.0	3.6
Mann-Whitney p-value	.	0.62	0.88	0.66	0.15	0.54	0.98	0.03	0.41

Figure 6. HADS anxiety and depression scores by treatment allocation and over visits

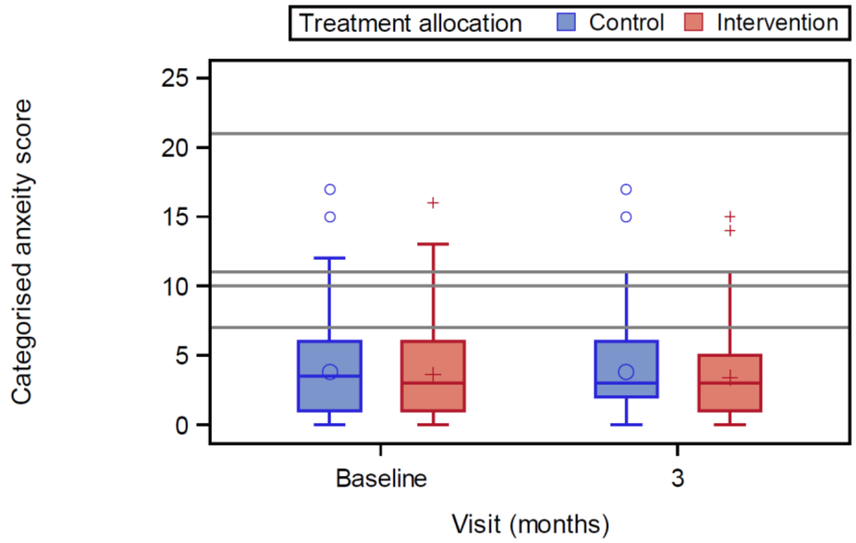

Mann-Whitney U test				
	Control		Intervention	
	Baseline	3 months	Baseline	3 months
Median	3.5	3.0	3.0	3.0
Q1-Q3	1.0-6.0	2.0.-6.0	1.0-6.0	1.0-5.0
Range	0-17.0	0-17.0	0.0-16.0	0.0-15.0
N	184	157	191	142
Mann-Whitney U test 3 months, p-value=				0.18

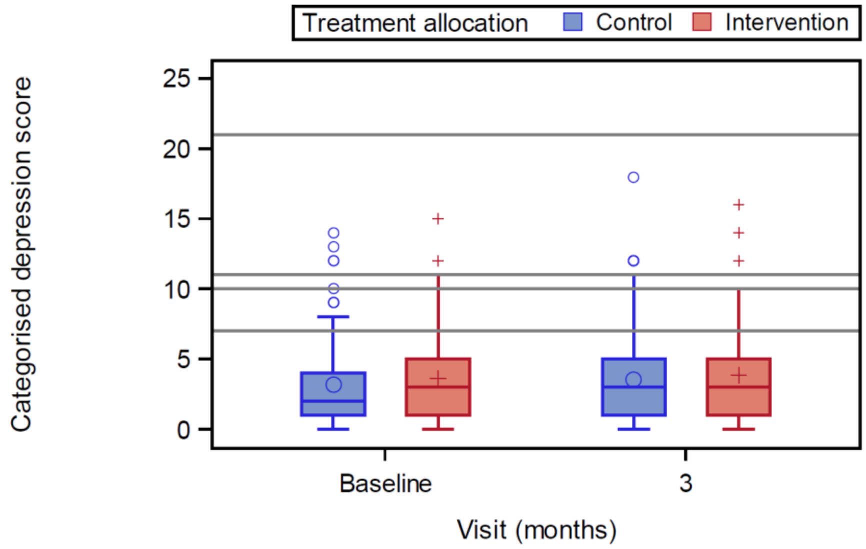

Mann-Whitney U test				
	Control		Intervention	
	Baseline	3 months	Baseline	3 months
Median	3.0	3.0	3.0.	3.0
Q1-Q3	1.0-4.0	1.0-5.0	1.0-5.0	1.0-5.0
Range	0.0-14.0	0.0-18.0	0.0-15.0	0.0-16.0
N	184	157	191	142
Mann-Whitney U test 3 months, p-value=				0.18

Normal (0-7), mild (8-10), moderate (11-14), severe (15-21).

Figure 7. EQ-5D-5L: Health State today by treatment allocation and over visits

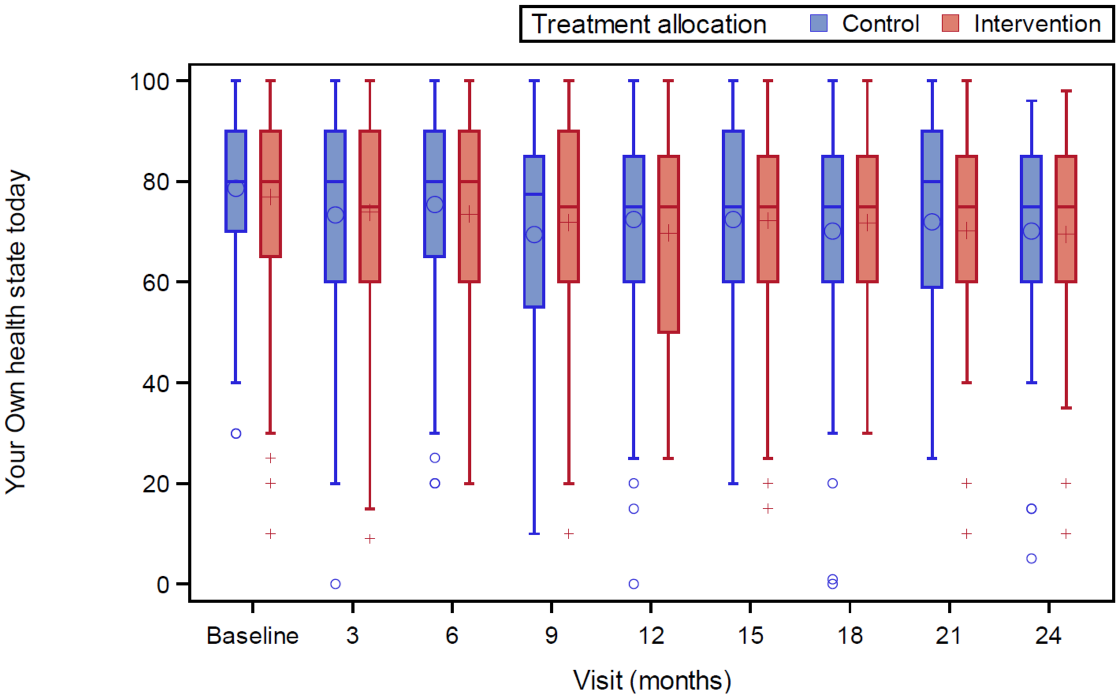

Statistics	Baseline	3	6	9	12	15	18	21	24
N (Control)	204	174	160	144	123	101	86	68	52
Median(C)	80.0	80.0	80.0	77.5	75.0	75.0	75.0	80.0	75.0
Q1(C)	70.0	60.0	65.0	55.0	60.0	60.0	60.0	59.0	60.0
Q3(C)	90.0	90.0	90.0	85.0	85.0	90.0	85.0	90.0	85.0
N (Intervention)	203	172	156	139	115	100	85	67	53
Median (I)	80.0	75.0	80.0	75.0	75.0	75.0	75.0	75.0	75.0
Q1(I)	65.0	60.0	60.0	60.0	50.0	60.0	60.0	60.0	60.0
Q3(I)	90.0	90.0	90.0	90.0	85.0	85.0	85.0	85.0	85.0
Mann-Whitney p-value	.	0.72	0.53	0.71	0.2	0.76	0.89	0.5	0.72

Table 11. Causes of death in control and intervention groups

Cause of death	Treatment allocation					
	Control		Intervention		Total	
	N	%	N	%	N	%
**Prostate cancer**	158	91	150	87	308	89
**Cardiovascular disease**	2	1	10	6	12	4
**Respiratory cause**	4	2	4	2	8	2
**Accident/trauma**	1	1	1	1	2	1
**GI perforation/haemorrhage**	1	1	0	0	1	<1
**Other malignancy**	0	0	1	1	1	<1
**Metabolic/endocrine causes**	0	0	1	1	1	<1
**Cerebro-vascular accident**	1	1	0	0	1	<1
**Unknown**	7	4	5	3	12	4
**Total**	174		172		346	

Table 12. Cox regression model for overall survival

Model	Covariates included in model	Comparisons	Hazard ratio		
			Estimate	95 % confidence Interval	P-value
Univariable N=420	Treatment only	Intervention vs Control	0.97	0.79-1.20	0.810
Multivariable N=419	Treatment	Intervention vs control	0.98	0.78-1.20	0.84
	ALP raised	Raised vs Normal	1.88	1.51-2.35	<.0001
	Previous treatment	2nd or later vs 1st line	1.13	0.89-1.44	0.31
	Previous spinal RT	Yes vs No	0.96	0.75-1.13	0.60
	CT within 6mths	Yes vs No	1.05	0.82-1.34	0.71
	Time from CRPC	Per year	1.00	0.95-1.05	0.97
	ECOG	EGOG 1&2 vs 0	1.55	1.24-1.93	0.0001
	Ln PSA	1 unit rise	1.28	1.18-1.49	<.0001

Table 13. Types of additional systemic treatment received by patients in control and intervention group within 12 months of randomisation

Systemic treatment	Treatment allocation			
	Control		Intervention	
	N	%	N	%
**Endocrine therapy**	102	49	91	43
**Chemotherapy**	55	26	31	15
**Radioisotopes**	22	11	12	6
**Bone-protection**	14	7	6	3
**Unknown**	1	1	0	0
**Total**	147		113	

Patients are only included once in each treatment type and overall total.
Excludes short term corticosteroids as part of treatment for cSCC

Table 14. Spinal radiotherapy in control and intervention groups: Number of patients treated and radiotherapy courses delivered within 12 and 24 months of randomisation

	12 months		24 months	
	Courses, N	Patients, N (%)	Courses, N	Patients, N (%)
**Control group (210)**				
**rSCC /cSCC**	32	28(13%)	43	37(18%)
**Other reason for spinal RT**	17	16(8%)	19	17(8%)
**Total**	49	41(21%)	62	48(26%)
**Intervention group (210)**				
**rSCC /cSCC**	78	66(31%)	91	76(36%)
**Other reason for spinal RT**	8	8(4%)	16	14(7%)
**Total**	86	71(35%)	107	85(43%)
**Intervention group rSCC+ (61): rSCC /cSCC**				
**rSCC /cSCC**	69 (51)[1]	57(93%)	71(51)[1]	57(93%)
**Other reason for spinal RT**	2	2 (3%)	6	4(7%)
**Total**	71	57(97%)	81	57(93%)
**Intervention group rSCC- (149): rSCC /cSCC**				
**rSCC /cSCC**	9	9(6%)	20	19(13%)
**Other reason for spinal RT**	6	6(4%)	10	10(7%)
**Total**	15	14(10%)	30	28(20%)

1. For rSCC on screening MRI

Table 15. MRI in control and intervention groups: Number of protocol and clinically indicated scans performed within 24 months of randomisation

Visit	Treatment allocation						
	Control			Intervention			
	Any MRI		Number of patients	Any MRI (accept Protocol)		Number of patients	Protocol MRI[4]
	N[1]	%[2]	N	N[1,3]	%[2]	N	N
**6 months**	49 (55)	25	196	26 (28)	14	193	35 (35)
**12 months**	18 (20)	11	159	13 (15)	8	162	25 (25)
**18 months**	15 (16)	12	127	18 (20)	15	121	16 (16)
**24 months**	7 (7)	8	88	10 (11)	11	92	9 (9)
**Total**	89 (98)			67 (74)			85 (85)

1. N represents number of patients having MRI during time period, numbers in brackets are actual number of MRIs.
2. Percentages are based on number of patients still in follow up at end of time point.
3. Number in intervention include both rSCC+ve and –ve patients
4. Scans performed according to protocol during follow up. All screening MRI were performed in the intervention group

Protocol

A Prospective Randomised Phase III Study of Observation Versus Screening MRI And Pre-Emptive Treatment in Castrate Resistant Prostate Cancer Patients With Spinal Metastasis

PROMPTS

**Chief Investigator:**	Professor David Dearnaley
**Sponsor:**	The Institute of Cancer Research
**Approval:**	Clinical Trials Advisory & Awards Committee (CTAAC)
**Funders:**	Cancer Research UK
**Coordinating Trials Unit:**	ICR Clinical Trials and Statistics Unit (ICR-CTSU)<br>The Institute of Cancer Research

PROTOCOL VERSION 7.0
29 November 2016

**ICR-CTSU Protocol Number:**	**ICR-CTSU/2012/10035**
**Main REC Reference Number:**	**12/LO/1109**
**ISRCTN:**	**ISRCTN74112318**
**Funder's Reference Number:**	**CRUK/11/053**

The PROMPTS trial has been scientifically approved by Cancer Research UK's Clinical Trials Advisory & Awards Committee (CTAAC)

The PROMPTS trial is part of the National Institute for Health Research Clinical Research Network Trial Portfolio

CANCER RESEARCH UK

This protocol is a controlled document and should not be copied, distributed or reproduced without the written permission of the ICR-CTSU

ADMINISTRATION

Clinical Co-ordination

Professor David Dearnaley (Chief Investigator)
Consultant Clinical Oncologist
ICR – Academic Radiotherapy
The Institute of Cancer Research
London

Tel: 0208 661 3271
E-mail: David.Dearnaley@icr.ac.uk

Trial Coordination

ICR-CTSU (a UKCRC registered and NCRI accredited clinical trials unit) is responsible for the day to day conduct of the trial.

ICR Clinical Trials & Statistics Unit (ICR-CTSU),
Division of Clinical Studies,
The Institute of Cancer Research,
Sir Richard Doll Building,
Cotswold Road,
Sutton, Surrey SM2 5NG

ICR-CTSU Scientific Lead: Emma Hall
Tel: 020 8722 4292
Emma.Hall@icr.ac.uk

Statistician: Helen Mossop
Tel: 020 8722 4008
Helen.Mossop@icr.ac.uk

Senior Trial Manager: Clare Cruickshank
Tel: 020 8722 4058
Clare.Cruickshank@icr.ac.uk

PROMPTS Trial Manager:
Shama Hassan
Tel: 020 8722 4183
prompts-icrctsu@icr.ac.uk

Any questions relating to this protocol should be addressed in the first instance to the PROMPTS Trial Manager within ICR-CTSU:

Email: prompts-icrctsu@icr.ac.uk
General enquiries: 020 8722 4183
Fax: 020 8770 7876

Protocol Development Group

David Dearnaley	Chief Investigator	ICR-Academic Radiotherapy, The Institute of Cancer Research, London.
Emma Hall	Deputy Director	ICR-CTSU, The Institute of Cancer Research, London.
Ann Henry	Co-investigator	St, James's University Hospital
Pamela Levack	Co-investigator	Ninewells Hospital, Dundee
Malcolm Mason	Co-investigator	Cardiff University
Alec Miners	Co-investigator	London School of Hygiene and Tropical Medicine
Philip Rich	Co-investigator	St. George's Healthcare NHS Trust
Aslam Sohaib	Collaborator	The Royal Marsden NHS Trust
Ramachandran Venkitaraman	Co-investigator	The Ipswich Hospital NHS Trust
Clare Cruickshank	Senior Trial Manager	ICR-CTSU, The Institute of Cancer Research, London.
Clare Griffin	Statistician	ICR-CTSU, The Institute of Cancer Research, London

The Trial Management Group (TMG) will be constituted from members of the Protocol Development Group and Principal Investigators from a subset of participating centres. A copy of the current membership of the TMG can be obtained from the PROMPTS Trial Manager at ICR-CTSU.

Protocol Authorised by:

Name & Role	Signature	Date
Professor David Dearnaley (Chief Investigator)	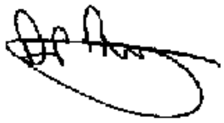	29/11/2016

This protocol describes the PROMPTS trial and provides information about procedures for entering patients. The protocol should not be used as a guide for the treatment of other patients. Every care was taken in the preparation of this protocol, but corrections or amendments may be necessary. These will be circulated to investigators in the trial, but centres entering patients for the first time are advised to contact ICR-CTSU to confirm they have the most recent version. Protocol amendments will be circulated to participating centres as they occur.

This trial will be conducted in accordance with the professional and regulatory standards required for non-commercial research in the NHS under the Research Governance Framework for Health and Social Care. It will be conducted in compliance with the protocol, the Data Protection Act (Z6364106) and other regulatory requirements as appropriate.

CONTENTS

List of Abbreviations.......... v
Practical definitions for the purpose of the protocol v
TRIAL SUMMARY vi
1. Introduction 1
1.1. Malignant Spinal Cord Compression 1
1.2. MRI spine to detect spinal cord compression 1
1.3. Frequency of screening MRI Spine 2
1.4. Treatments for early spinal cord compression 3
1.5. DEFINITION OF TERMS 4
1.6. Rationale for Study 5
2. Trial objectives 5
2.1. Primary Objective 5
2.2. Secondary Objectives 5
3. Trial design 5
3.1. Figure 1: STUDY FLOW CHART 6
4. Patient selection & eligibility 6
4.1. Source of Patients 6
4.2. Number of Patients 6
4.3. Inclusion Criteria 6
4.4. Exclusion Criteria 7
5. RANDOMISATION AND TREATMENT ALLOCATION 7
5.1. Randomisation procedure 7
6. Trial assessments 8
6.1. Baseline Assessments 8
6.2. On-study Assessments 8
Assessments for intervention group patients 8
Assessments at each neurologic event 8
6.3. Table 1 SCHEDULE OF ASSESSMENTS 10
7. Trial intervention 11
7.1. Group 1 (control): 11
7.2. Group 2 (INTERVENTION GROUP): 11
8. Management of patients in the study who develop rSCC or cSCC 11
8.1. MRI protocol 11
8.2. TREATMENT protocol 12
Dose and Fractionation 12
Planning technique 12
8.3. Assessments during each episode of SCC. 13
8.4. Systemic treatment 13
9. EVALUATION OF OUTCOME 13
9.1. DEFINITIONs of CLINICAL and RADIOLOGICAL SCC 13
9.2. Definition of findings on MRI spine. 14
Practical definitions for the purpose of the protocol 14
9.3. Definition of Functional Neurologic Deficit due to SCC. 14
10. CONCURRENT MEDICATIONS 15
11. SAFETY REPORTING 15
11.1. Definition Of An Adverse Event (AE) 15
11.2. Definition Of Related Adverse Event 15
11.3. Reporting Of Adverse Events 15
11.4. Definition Of Serious Adverse Events (SAEs) 15
11.5. Events Not Subject To Expedited Reporting: 16
11.6. SAE Causality 16
Definitions for SAE causality 16
11.7. Reporting Serious Adverse Events 17
11.8. Review Of Serious Adverse Events (SAEs) 17
11.9. Expedited Reporting Of Related Unexpected SAEs 17
11.10. Follow Up Of Serious Adverse Events 17

11.11. Annual Reporting Of Serious Adverse Events 18
12. Statistical considerations 18
12.1. Sample size calculation 18
12.2. ENDPOINT DEFINITIONS 18
12.3. Statistical ANALYSIS 19
13. TRIAL MANAGEMENT 21
13.1. Trial Management Group (TMG) 21
13.2. Trial Steering Committee (TSC) 21
13.3. Independent Data Monitoring Committee (IDMC) 21
14. RESEARCH GOVERNANCE 21
14.1. Sponsor Responsibilities 21
14.2. Responsibilities Of Chief Investigator 21
14.3. Responsibilities Of ICR-CTSU 22
14.4. Responsibilities Of Participating Centres 22
15. TRIAL ADMINISTRATION AND LOGISTICS 22
15.1. Protocol Compliance 22
15.2. Investigator Training 22
15.3. Data Acquisition 23
15.4. Central Data Monitoring 23
15.5. On Site Monitoring 23
15.6. Protocol Amendments 24
15.7. End Of Study 24
15.8. Archiving 24
16. PATIENT PROTECTION AND ETHICAL CONSIDERATIONS 24
16.1. Risk Assessment 24
16.2. Patient Confidentiality 24
16.3. Ethical Considerations 24
16.4. Patient Information 25
16.5. Data Sharing 25
16.6. Data Protection Act (DPA) 25
16.7. Liability/Indemnity/Insurance 25
17. FINANCIAL MATTERS 25
18. PUBLICATION POLICY 25
19. Associated studies 26
19.1. Quality of Life 26
19.2. Health Economics 26
References 26
Appendix A - ECOG Scale 28
Appendix B – Frankel Spinal Cord Injury Assessment Tool. 29
Appendix C – Bilsky Spinal Cord Compression Scale[53 (modified)] 31

List of Abbreviations

**AE**	Adverse event
**BPI**	Brief Pain Inventory
**CRAG**	Clinical Resource Audit Group
**CRF**	Case report form
**CRPC**	Castrate Resistant Prostate Cancer
**ECOG**	Eastern Cooperative Oncology Group
**EORTC**	European Organisation for Research and Treatment of Cancer
**EQ-5D**	EuroQol-5 dimensions
**FND**	Functional neurologic deficit
**GCP**	Good clinical practice
**ICR**	The Institute of Cancer Research
**ICR-CTSU**	The Institute of Cancer Research Clinical Trials and Statistics Unit
**LHRH**	Leutinising hormone releasing hormone
**MRI**	Magnetic resonance imaging
**NICE**	National Institute for Clinical Excellence
**PSA**	Prostate specific antigen
**QALYS**	Quality adjusted life years
**QL**	Quality of Life
**RT**	Radiotherapy
**SAE**	Serious adverse event
**SCC**	Spinal cord compression
	**cSCC** Clinical spinal cord compromise or compression (see below)
	**rSCC** Radiological spinal canal/cord compromise or compression (see below)
**WBC**	White blood cell count

Practical definitions for the purpose of the protocol

This section outlines the cSCC and rSCC definitions in terms of Bilsky scoring (Appendix C). The terms cSCC and rSCC will be used throughout the protocol.

cSCC – Clinical spinal cord compromise or compression – patients are symptomatic

Clinical spinal cord compromise –Bilsky score 1a-c (Bilsky 1a-b would be exceptional)

Clinical spinal cord compression – Bilsky score 2 or 3

rSCC – Radiological spinal canal/cord compromise or compression – patients are asymptomatic

Radiological spinal canal compromise – Bilsky score 1a or 1b

Radiological spinal cord compromise – Bilsky score 1c

Radiological spinal cord compression – Bilsky score 2 or 3

TRIAL SUMMARY

TITLE

A **P**rospective **R**andomised Phase III Study of **O**bservation Versus Screening **M**RI And Pre-Emptive **T**reatment in Castrate Resistant Prostate Cancer Patients With **S**pinal Metastasis

STUDY OBJECTIVES:

Primary: Does detection of radiological spinal cord/canal compression (rSCC) by screening MRI of the spine and pre-emptive treatment reduce the incidence of clinical spinal cord/canal compromise or compression (cSCC) in asymptomatic castrate resistant prostate cancer (CRPC) patients with spinal metastasis?

Secondary:

- What is the utility of screening magnetic resonance imaging (MRI) in detecting rSCC in patients with asymptomatic spinal metastases?
- How does early intervention and prophylactic treatment of rSCC affect the development of cSCC?
- What is the effect of screening MRI and prophylactic treatment on;
    - preservation of neurological function;
    - rates of subsequent spinal radiotherapy and surgery;
    - subsequent mobility, pain and health related quality of life;
    - survival;
    - cost effectiveness?

TRIAL DESIGN: Prospective, randomised, two-group, non-blinded, phase III, interventional study

PATIENT TYPE: The target population is patients with CRPC with proven spinal metastasis, and with no neurologic symptoms.

SAMPLE SIZE 414 patients

TRIAL TREATMENT: Patients will be randomised in a 1:1 ratio to one of the following two treatment groups:

• Control group: patients followed up as per standard practice i.e., in accordance with National Institute of Clinical Excellence (NICE) guidelines, MRI spine performed if patient develops clinical neurological deficit or significant spinal pain with treatment given if there is clinical (c)SCC on MRI;

• Intervention group: Baseline screening MRI and pre-emptive treatment to sites of radiological (r) SCC; following detection of rSCC and pre-emptive treatment patients will receive an MRI scan every 6 months. rSCC is defined according to the Bilksy scoring system (see page v of this protocol).

ENDPOINTS:

Primary: Incidence of cSCC at one year and time to development of confirmed cSCC.

Secondary:

- Rate of detection of rSCC (Bilsky 1a -3) on the baseline screening MRI (in the intervention group only).
- Incidence of and time to functional neurological deficit due to cSCC.
- Incidence of and time to irreversible functional neurological deficit due to cSCC.
- Incidence of SCC (Bilsky 1-3) in both the control and intervention groups during follow-up.
- Pain, quality of life, overall survival, cost effectiveness.

1. INTRODUCTION

1.1. MALIGNANT SPINAL CORD COMPRESSION

Spinal cord compression (SCC), the most clinically significant complication due to spinal skeletal metastasis, is reported to occur in 3 - 10 % of cancer patients resulting in significant debility and impact on quality of life[1-3]. Patients with breast, lung and prostate cancer account for about 60% of metastatic SCC cases but it can be caused by any malignancy[4]. The risk of SCC is also proportionally related to the duration of disease and therefore, as cancer survival times increase, so too does the incidence of SCC. SCC occurs when there is pathological vertebral body collapse or direct tumour growth causing compression of the spinal cord or cauda equina. The development of SCC in patients with metastatic cancer is a clinical disaster, resulting in neurological deficit causing paraplegia which may be irreversible[5].

SCC and its complications have a profound influence on the functional, social, emotional and physical quality of life of the patient with a resulting increased burden on the health care system. SCC is reported to be associated with a doubling of the time spent in hospital in the last year of life[6]. Complications of motor deficit from SCC such as pneumonia and thrombotic events are known to adversely impact on survival, and it could be postulated that the prevention of functional neurologic deficit by early diagnosis and treatment, might reduce the adverse influence of SCC on survival. In addition to the poor quality of life, the high cost of emergency hospital admissions, palliative care and rehabilitation may be more demanding on resources than the prevention of SCC with early imaging and prophylactic treatment [7-9].

Incidence of SCC and its clinical outcome has an important effect on overall survival in patients with metastatic cancer, though the outcome is also influenced by the aggressiveness of the underlying primary malignancy. In patients with SCC, primary tumours like breast and prostate have a favourable outcome ranging from 12-18 months, compared to tumours like lung where the median survival would be approximately 6 months[10-12].

Early diagnosis of SCC is essential, as pre-treatment neurologic status is the major determinant influencing outcome[2,5,11,13]. In the study by Husband et al of 301 patients with SCC, lack of symptom recognition by the patient and diagnostic delay by the physician resulted in preventable loss of neurologic function in approximately 70 % of patients, the median delay being 14 days [14]. In a prospective observational study of 319 patients with SCC by Levack et al, 82% of whom were non-ambulant, weakness and sensory abnormalities were reported late, despite 94% of patients reporting pain for approximately 3 months. The delay in detection of SCC was due to delay in referral and investigation of a median of 66 days, suggesting that patients with cancer who describe severe back pain or spinal nerve root pain need urgent assessment by MRI spine on the basis of their symptoms, as signs may occur too late[4].

Studies have suggested that approximately 80% of patients who were ambulant pre-treatment, would remain ambulant after treatment, while only 15 – 30 % of patients who were non-ambulant would be expected to regain ambulant status following treatment, the rate of recovery very much dependent on the level of neurologic deficit[15-20]. The results of several studies including the Clinical Resource Audit Group (CRAG audit) study, suggests that the patients who were ambulant prior to treatment for SCC and patients who remained ambulant after treatment, had a statistically significant longer survival, with systemic relapse being the commonest cause of death[4,16,21].

1.2. MRI SPINE TO DETECT SPINAL CORD COMPRESSION

Clinical signs are unreliable indicators of the presence or the level of suspected SCC and MRI of the spine is considered a mandatory investigation for detecting SCC and for planning management[2,3,7-9]

Abnormal neurologic examination, back pain, metastatic disease at diagnosis and extensive skeletal metastasis were found to be independent clinical predictors of SCC in 134 cancer patients evaluated with MRI spine by Lu et al[23]. In this study 100 patients had a normal neurologic

examination, of whom 30 had thecal sac compression on MRI spine, with back pain and metastatic disease at presentation being the significant predictors. In the study by Talcott in patients with various malignancies where CT scans were utilized to detect SCC, inability to walk, increased deep tendon reflexes, compression fractures on radiographs of spine, bone metastases present, bone metastases diagnosed more than 1 year earlier, and age less than 60 years were risk factors for developing SCC[24]. In the study by Venkitaraman et al in metastatic prostate cancer patients, back pain was an independent predictor of future adverse neurological outcome[25].

The NICE clinical guideline 75 suggests that cancer patients with symptoms or signs suggestive of spinal metastasis and neurologic symptoms or signs suggestive of SCC including radicular pain, any limb weakness, difficulty in walking sensory loss or bowel or bladder dysfunction or any neurological signs of spinal cord or cauda equina compression should be considered as an oncologic emergency and should have an urgent MRI spine to detect SCC[1].

Once a diagnosis of SCC has been made on MRI, the treatment goals include pain relief, restoration of neurological status, prevention of further neurological damage and stabilisation of the spine[26]. MRI spine is also essential for planning surgery or radiotherapy for SCC because of the better delineation of extent of disease[26].

In prostate cancer, investigations have shown that it is possible to detect early radiological signs of impending SCC (radiological spinal canal/cord compromise or compression (rSCC)) in asymptomatic patients with or without bone pain. The definitions used for SCC on MRI scan have varied in different studies. Bayley et al, had used a definition for rSCC of 'impingement of the subarachnoid space by metastatic tumour involving the vertebrae or bone fragments, or frank compression of cord or cauda equina'[8]. Similarly, Venkitaraman et al, had used the definition of 'involvement or compression of either the spinal cord or the cauda equina by an epidural or an intramedullary mass lesion metastatic disease causing impingement, indentation or loss of definition of the thecal sac'[22]. Lu et al, had used a definition of 'thecal sac compression' in their study[23].

Detection of rSCC before clinical manifestations such as neurologic deficit or intractable pain by MRI spine may provide an important lead time for early treatment and thus may minimize the likelihood of irreversible functional neurologic deterioration[26]. Bayley et al, detected rSCC in 22 of 68 patients (32%) with metastatic prostate cancer with no functional neurologic deficit (FND), the extent of disease on bone scan and the duration of continuous hormonal therapy being independent predictive factors[8]. In the study by Venkitaraman et al, 41 of 150 patients (27.33 %) with no FND were detected to have rSCC by MRI spine with the presence of back pain and extensive bone metastasis being the most important predictors[22]. Godersky et al, detected rSCC in 5 out of 22 patients (23 %) with back pain and without neurologic deficit[27]. As there is clinical evidence that patients with neurologic back pain do benefit from investigations to rule out SCC, further research to evaluate the benefits of screening MRI and pre-emptive treatment may need to target the subgroup of asymptomatic patients with spinal metastasis. This is one of the key research recommendations of the NICE guideline CG75 committee[1].

1.3. FREQUENCY OF SCREENING MRI SPINE

No prospective information is available regarding the proportion of patients with rSCC who would go on to develop neurologic symptoms, as it would be unethical not to treat them. The risk of SCC may be expected to increase with longer survival. In the study by Bayley et al, the risk of developing cSCC within 1 year of a negative screening MRI was 3.2% and the risk of developing cSCC within 2 years of a negative screening MRI was 13.7%[8]. If serial screening MRI spine are planned to detect SCC in 90 % of patients prior to development of neurologic signs, the optimum frequency could range from approximately every three months to every twelve months, considering the wide variation in patient criteria and results reported in various studies and also the metastatic potential and aggressiveness of the underlying primary malignancy. As the expected median survival for patients with spinal metastasis could range from 12-36 months depending on the

primary malignancy, a prospective screening study would involve an average of approximately 2-5 MRI scans, depending on the characteristics of the study group[54].

In PROMPTS a single screening MRI scan will be performed and we shall follow patients to determine what the appropriate repeat screening frequency might be. In patients who have rSCC found on screening, 6 monthly repeat scans are mandated as previous studies have demonstrated a high rate of progression[6,25,54].

1.4. TREATMENTS FOR EARLY SPINAL CORD COMPRESSION

It has been suggested that high-risk patients should undergo MRI screening with the aim of diagnosing and treating incipient SCC before development of FND[1,8]. Detection of rSCC by MRI spine before the development of cSCC and early institution of treatment might preserve neurologic function in the majority of patients. Complications of motor deficit from SCC such as pneumonia and thrombotic events are known to adversely impact on survival, and it could be postulated that the prevention of functional neurologic deficit by early detection and treatment of SCC would reduce the adverse influence of the occurrence of such an event on survival.

The treatment options for SCC include high dose corticosteroids, surgery and radiotherapy. High dose corticosteroids, especially Dexamethasone, has been shown to improve ambulation when given in combination with radiotherapy in patients who had SCC [2,28,29]. The role of maintenance steroids and their role in patients who already have a good motor function is controversial[30,31].

Radiotherapy is proven to be an effective treatment for cSCC from metastatic disease, especially in ambulatory patients without bony instability[5,11,13,32,33]. Almost all patients with SCC who are ambulant prior to treatment would retain motor function and are expected to be ambulant after radiotherapy[2]. Radiotherapy causes tumour decompression, may reduce venous congestion and prevent arterial infarction, which have been postulated to be responsible for neurologic injury in SCC[3,32,34]. Tumours with favorable histology like breast, myeloma and prostate have been reported to be associated with a longer median response to radiotherapy[12]. Kaplan et al have shown a reduction in spinal metastasis after pre-emptive radiation in prostate cancer[35]. Whether the same would hold true for SCC from spinal metastasis requires investigation. Helweg-Larsen and colleagues irradiated symptomatic synchronous compressions with a two vertebral body margin. At a median follow-up of 3.5 years, none of the 14 patients who had lesions within the irradiated volume relapsed in the same area as the previous lesion[36]. In a retrospective study by Soerdjbalie-Maikoe et al, none of the patients who received local radiotherapy for spinal metastasis developed SCC[37]. In the prospective study by Maranzano et al, 20 patients with no signs of neurologic spinal compression received 30 Gy in 10 fractions with no steroids to sites of subclinical SCC. All patients (100%) responded to radiotherapy because the 16 patients able to walk without support at diagnosis did not deteriorate and the other 4, who needed support, became ambulatory without motor impairment[30]. The findings from the retrospective study by Venkitaraman et al, also suggest that radiotherapy may prevent neurologic deficit in case of rSCC[25]. This hypothesis is also supported by the tumour response and resolution of cord compression evident on post radiotherapy MRI scans.

The dose of radiotherapy has varied in different studies. Rades et al, in three different studies comparing different radiotherapy regimes did not find a significant difference in neurologic outcome between doses ranging from 8Gy x 1, 4Gy x 5, 3Gy x 10, 2.5Gy x 15 and 2Gy x 20[38-40]. The infield recurrences at two years though were lower for longer courses of treatment. However, in another prospective study by the same group short course radiotherapy was found to be similar to long course radiotherapy (≥ 30 Gy) for functional outcome and overall survival, but resulted in inferior progression free survival and local control[41]. Similarly Maranzano et al, did not detect any difference in two split course regimes of 5 Gy x3 followed by 3 Gy x5 or 8 Gy x2[42]. In patients with prostate cancer who had SCC, overall response to radiotherapy has been reported to be 86% (33% improvement of motor function, 53% no further progression), with 33% of the non-ambulatory patients regaining the ability to walk. In this study the 2 year local control of SCC was 84% , with better results after long course radiotherapy[5]. Results of the meta-analysis of patients in randomized trials of single versus multiple fractions of radiotherapy for painful bone metastasis,

suggest no significant difference in the incidence of SCC for either regimes, though there was a trend for lower SCCs in patients who received fractionated radiotherapy[43]. The local practice in the UK has been to offer 20 Gy in 5 daily fractions over a week for patients with SCC.

Surgical decompression and stabilisation has been found to result in better neurologic outcome than radiotherapy in patients with bony compression or unstable spine. In the meta-analysis by Klimo et al the percentage of patients remaining ambulant after radiotherapy or surgery for SCC were found to be 64 % and 85 % respectively, with surgical patients twice as likely to regain ambulatory function[44]. In retrospective studies decompressive laminectomy followed by radiotherapy has been reported to have a better functional response than patients treated with either surgery or radiotherapy alone [20,45]. In the prospective randomised study by Patchell et al direct decompressive surgery and postoperative radiotherapy was found to be superior to radiotherapy alone for patients with metastatic SCC, with the percentage of patients ambulant being 84 % and 54 % respectively in the two groups[46]. In a study of 81 patients, emergency surgical spinal decompression (61.5 %) led to better outcomes compared to elective surgery (25 %), despite initial delays in referral and even if the patient were incontinent and immobile[47]. Surgery in patients with vertebral metastasis without neural deficit has been reported to result in substantial functional improvement, but with no improvement in survival[48].

Systemic treatments like chemotherapy and hormonal treatment for individual primary tumours have been shown to reduce disease progression and may reduce complications like metastatic SCC. Patients with bone metastasis disease receiving bisphosphonates have been shown to have significantly reduced incidence of skeletal related complications, and may have a reduction in the incidence of SCC[37]. There is a suggestion that bisphosphonate use in patients with SCC may improve functional outcome and even overall survival[41].

An economic evaluation as part of the NICE guidelines has shown the cost-effectiveness of the main treatment options available for SCC i.e. surgery and radiotherapy compared to no treatment[1].

In spite of all these treatments, the prognosis of CRPC patients with malignant SCC is bleak and further research is warranted aiming to prevent the onset of this complication. CRPC patients with spinal metastasis would be an ideal patient group for studies of early imaging to detect spinal cord compromise and prophylactic treatment to prevent neurologic deficit, and such research would be a priority area for the NHS[1].

1.5. DEFINITION OF TERMS

The validated **Bilsky** epidural SCC scoring system[53] evaluates metastatic disease causing impingement, indentation or loss of definition of the thecal sac or frank compression of spinal cord or cauda equina using a 6 point scale (0,1a-c,2,3) (Appendix C).

Clinical (or overt) SCC is defined in the NICE metastatic SCC guidelines as. "spinal cord or cauda equina compression by direct pressure and/or induction of vertebral collapse or instability by metastatic spread or direct extension of malignancy that threatens or causes neurological disability". For the purposes of the PROMPTS protocol patients with cSCC will be symptomatic with imaging confirming cSCC according to the NICE definition. Imaging will be scored according to the Bilsky system (appendix C). Patients with cSCC will usually have Bilsky scores of 2 or 3.

Radiologic (or occult) SCC (rSCC) will be defined by MRI in asymptomatic patients: MRI findings will usually give a Bilsky 1a- 1c score but exceptionally Bilsky 2-3 scores may be found in asymptomatic patients.

1.6. RATIONALE FOR STUDY

To determine whether the early detection of rSCC by screening MRI spine and pre-emptive treatment with radiotherapy facilitates preservation of neurologic function in CRPC patients with spinal metastasis.

2. TRIAL OBJECTIVES

2.1. PRIMARY OBJECTIVE

Does detection of rSCC by screening MRI of the spine and pre-emptive treatment reduce the incidence of cSCC in asymptomatic CRPC patients with spinal metastasis?

2.2. SECONDARY OBJECTIVES

- What is the utility of screening MRI in detecting rSCC in patients with asymptomatic spinal metastases?
- How does early intervention and prophylactic treatment of rSCC affect the development of cSCC? What is the effect of screening MRI and prophylactic treatment on:
    - preservation of neurological function;
    - rates of subsequent spinal radiotherapy and surgery;
    - subsequent mobility, pain and health related quality of life;
    - survival;
    - cost effectiveness?

3. TRIAL DESIGN

A multicentre prospective, randomised, two group, non-blinded, phase III interventional study in CRPC patients with spinal metastasis:

3.1. FIGURE 1: STUDY FLOW CHART

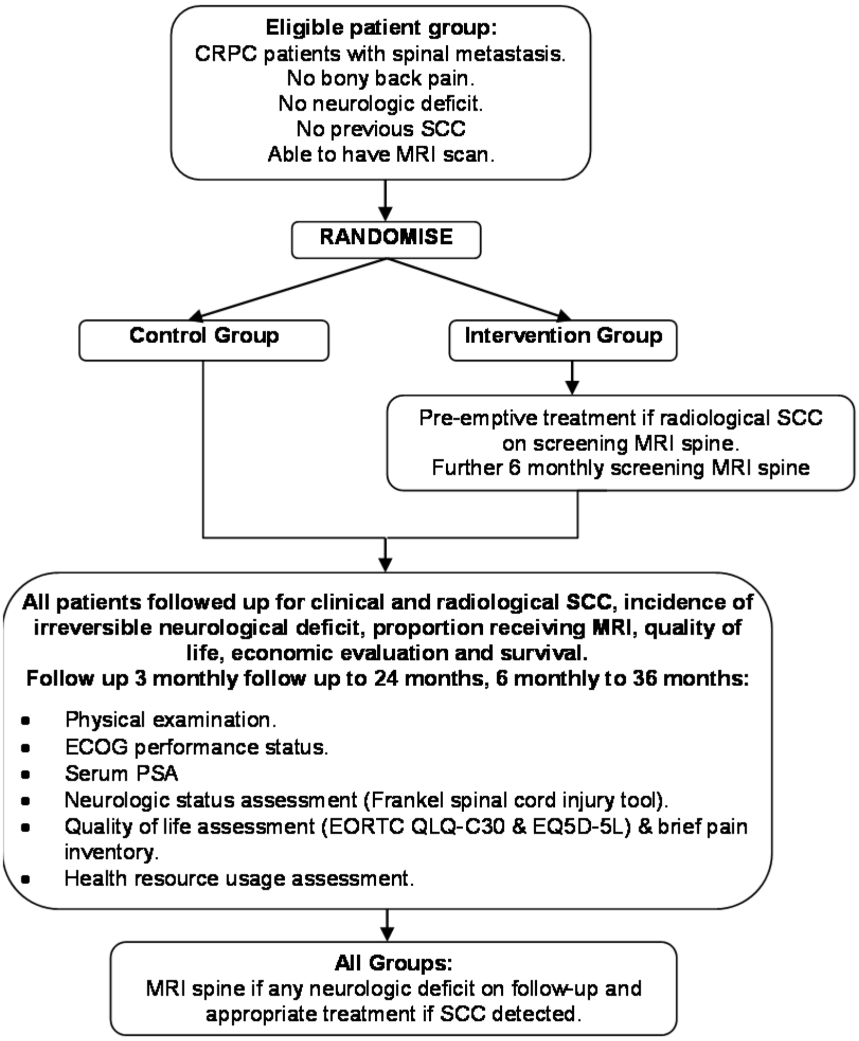

4. PATIENT SELECTION & ELIGIBILITY

4.1. SOURCE OF PATIENTS

The target population is patients with CRPC with proven spinal metastasis, and with no neurologic symptoms.

4.2. NUMBER OF PATIENTS

424 patients will be randomised in a 1:1 ratio to the control and intervention groups.

4.3. INCLUSION CRITERIA

- Either histologically/cytologically confirmed adenocarcinoma of the prostate **or** clinical diagnosis of prostate cancer with osteoblastic bone metastases and PSA≥ 100ng/ml at **any** time between diagnosis and randomisation;
- Castrate resistant disease;*
- PSA>5ng/ml within 21 days prior to randomisation
- One or more spinal* metastasis on imaging (by technetium bone scan with confirmatory x-ray as appropriate clinically, or by CT,PET-CT or MRI scan) undertaken at any time during the patient's illness.
- Life expectancy of 6 months or more;

- ECOG performance status 0-2;
- Written, informed consent.

rising PSA (>5 ng /ml **and >50% rise from nadir) after LHRHa therapy or orchidectomy with or without anti-androgen.*
** metastases in cervical, thoracic or lumbar spine*

4.4. EXCLUSION CRITERIA

- Back pain related to metastatic cancer, requiring regular (daily) analgesics which requires an immediate MRI as per NICE guidelines
- Previous malignancy that, in the opinion of the local investigator, makes it difficult to confirm that spinal metastases are secondary to prostate cancer or otherwise make the patient unsuitable for inclusion in the study.
- Current or previous SCC or neurologic deficit;
- Spinal MRI within last 12 months;
- Planned MRI of spine or thorax <u>AND</u> abdomen
- Previous external beam radiotherapy to the vertebra or spinal surgery with the primary aim to prevent or treat SCC;
- Serious or uncontrolled co-existent non-malignant diseases;
- Any contra indications for MRI;
- Inability to comply with neurologic and Quality of Life (QoL) assessments.

+ *(previous palliative radiotherapy to painful spinal metastases in now asymptomatic patients is permissible).*

5. RANDOMISATION AND TREATMENT ALLOCATION

5.1. RANDOMISATION PROCEDURE

Central randomisation will be performed by the Institute of Cancer Research Clinical Trials and Statistics Unit (ICR-CTSU).

Treatment allocation will be 1:1 and will use a minimization algorithm incorporating a random element. The following balancing factors will be used : centre, alkaline phosphatase (normal vs. raised), number of previous systemic treatments, excluding neo/adjuvant hormone therapy unless failed during treatment (i.e. whether the patient has had a first line treatment failure vs. second line or later),previous spinal radiotherapy and/or surgical procedure for metastatic disease and previous CT or PET CT scan of both thorax and abdomen within the last 6 months.

Once written informed consent has been obtained, an eligibility and randomisation checklist must be completed prior to randomisation. The clinician / research nurse should contact ICR-CTSU to confirm eligibility and obtain a unique trial number and treatment allocation.

The following information will be required at randomisation:

- Name of Hospital, consultant and person randomising patient;
- Confirmation that patient is eligible for the trial by completion of the checklist;
- Confirmation that patient has given written informed consent;
- Patient's full name, hospital number, date of birth, postcode and NHS/CHI number;
- Confirmation of alkaline phosphatase (normal vs raised), number of previous treatments (first failure vs second or later), details of any previous spinal radiotherapy and/or surgical procedure for metastatic disease (yes vs no) and detail of any CT or PET CT scan of both thorax AND abdomen within the last 6 months (yes vs no).
- PSA within 21 days prior to randomisation.

The caller will be given the patient's unique randomisation number (Trial ID). The Trial ID together with the patient's initials, date of birth and hospital number should be used on all Case Report

Forms (CRFs) and correspondence relating to the patient. To randomise a patient telephone:

ICR Clinical Trials and Statistics Unit (ICR-CTSU) The Institute of Cancer Research
020 8643 7150
09.00-17.00 Monday to Friday

6. TRIAL ASSESSMENTS

6.1. BASELINE ASSESSMENTS

The following should be done not more than 21 days prior to randomisation:

- Medical history.
- Physical examination (including neurologic assessment using Frankel spinal cord injury assessment tool (see appendix B) and evaluation of pain).
- ECOG performance status
- Haematology tests: Haemoglobin, white blood cell (WBC) count and platelet count.
- Clinical biochemistry tests: serum creatinine, alkaline phosphatase and serum albumin.
- Pre-trial clinical signs and symptoms using Common Toxicity Criteria for Adverse Event Reporting (CTCAEv4.0) grading.
- Serum prostate specific antigen (PSA).

The following should be done not more than 7 days prior to randomisation:

- Quality of life and neurologic assessment forms:
  - Brief Pain Inventory
  - EORTC QLQ-C30, EQ-5D-5L and HADS.

6.2. ON-STUDY ASSESSMENTS

Follow-up will take the following format:

Year 1: 3, 6, 9, 12 months
Year 2: 15, 18, 21, 24 months
Year 3: 30, 36 months
Year 4 onwards: annual Long Term Follow Up (LTFU)

At each of these time-points (except the annual LTFU) the following assessments will take place:

- Physical examination including neurologic assessment using Frankel spinal cord injury assessment tool (see appendix B).
- ECOG performance status
- Serum PSA
- EORTC QLQ-C30, EQ-5D-5L, Brief Pain Inventory, HADS to be completed by the patient at their clinic visit (HADS at 3 months only).

Assessments for intervention group patients

- Screening MRI (the screening MRI should be performed within 8 weeks following randomisation).
- MRI (every 6 months) for patients where rSCC has been seen on the screening scan.
- Health Economics questionnaire for patients with rSCC (once only approximately three months after treatment)

Assessments at each neurologic event

For any patient who has suffered a neurological event* the following assessments will take place:

- Physical examination including neurologic assessment using Frankel spinal cord injury assessment tool (see appendix B) (pre and post treatment)
- ECOG performance status (pre and post treatment)
- Pre and post treatment serum PSA
- Brief Pain Inventory (pre-treatment only) and EQ-5D-5L (pre and posttreatment) questionnaire to be completed by the patient at their clinic visit.
- Pre and post treatment toxicity assessments using CTCAEv4.0 grading (both radiotherapy and surgery) and Clavian Scale (surgery only).
- Health Economics questionnaire (approximately three months after treatment)

*(*detection of rSCC or cSCC)*

Once a patient has suffered a neurological event follow up remains the same as for patients who have not, as per protocol.

Discontinuation from follow-up

If a patient withdraws from further follow-up a trial deviation form should be submitted to ICR-CTSU stating whether the patient has withdrawn consent for information to be sent to the ICR-CTSU or whether they simply no longer wish to attend trial follow up visits. In the very rare event that a patient requests that their data is removed from the study entirely, the implications of this should be discussed with the patient first to ensure that this is their intent and, if confirmed, ICR-CTSU should be notified in writing. If this request is received after results have been published the course of action will be agreed between the Sponsor and independent Trial Steering Committee/Independent Data Monitoring and Steering Committee.

6.3. TABLE 1 SCHEDULE OF ASSESSMENTS

				Follow up month / year											
	**Screening** (within 21 days prior to randomisation)	**Baseline** (within 7 days prior to randomisation)	**After randomisation**	3	6	9	12	15	18	21	24	30	36	4 year onwards	At each neurologic event
Inclusion/Exclusion criteria	X														
Informed consent	X														
Demography	X														
Medical history	X														
ECOG	X			X	X	X	X	X	X	X	X	X	X		X
Physical examination[1]	X			X	X	X	X	X	X	X	X	X	X		X
Haematology & Biochemistry [2]	X[2]														
PSA	X			X	X	X	X	X	X	X	X	X	X		X
Quality of Life[3]		X		X	X	X	X	X	X	X	X	X	X		X
Control group															MRI[4]
Intervention group			MRI[4]		MRI[5]		MRI[5]		MRI[5]		MRI[5]	MRI[5]	MRI[5]		MRI[4]
Toxicity assessment[6]	X														X
Annual Long term follow up forms (LTFU)														X	

1. Including neurologic examination according Frankel spinal cord injury assessment tool (see appendix B).
2. This should include: haemoglobin, white blood cell (WBC) count, platelet count, serum creatinine, alkaline phosphatase and serum albumin.
3. Quality of Life assessment with EORTC QLQ C30, EQ-5D-5L, Brief Pain Inventory and HADS*****.
4. MRI denotes MRI of the spine
5. MRI (only for patients who have evidence of rSCC at screening (baseline) scan. *(If a patient cannot attend a scheduled scan appointment, the replacement appointment should be made as close to the original appointment as possible. The next scan after this, should keep at its original time.)*
6. Post treatment toxicity assessments using CTC AE grading (both radiotherapy and surgery) and Clavian Scale (surgery only).

***** HADS will only be completed at baseline and 3 months follow up. EORTC QLQ C30 not required at each neurologic event.

7. TRIAL INTERVENTION

7.1. GROUP 1 (CONTROL):

Control group: Patients will be followed up at three monthly intervals (from randomisation) for 2 years and then at 30 and 36 months with clinical examination patients. MRI spine performed if patient develops clinical neurological deficit or significant spinal pain with treatment given if there is cSCC on MRI, annual LTFU forms will be completed from year 4 onwards;

7.2. GROUP 2 (INTERVENTION GROUP):

Intervention group: If baseline screening is negative, follow up is as for the control group. If baseline screening MRI is positive pre-emptive treatment will be given to sites of rSCC. Following pre-emptive treatment patients will receive an MRI scan every 6 months. Patients will be followed up at three monthly intervals (from randomisation) for 2 years and then at 30 and 36 months, annual LTFU forms will be completed from year 4 onwards;

8. MANAGEMENT OF PATIENTS IN THE STUDY WHO DEVELOP rSCC OR cSCC

All patients who develop rSCC (Bilsky ≥1a) or cSCC (usually with Bilsky 2 or 3) should have their management reviewed in the specialist SCC MDT (if available) and the recommendations of the MDT should be followed. Alternatively the patient pathway should follow expert local practice. Most commonly, we expect radiotherapy to be the treatment of choice for both rSCC and cSCC. The recommended treatment for rSCC for patients in the study is with external beam radiotherapy which should be instituted within one week of confirmation of rSCC. Patients who develop cSCC or established neurological deficit should be treated according to the MDT recommendations with radiotherapy, spinal decompression or vertebro/kyphoplasty. For patients with rSCC or cSCC treated by radiotherapy, the recommended dose is 20Gy in 4Gy fractions daily over one week delivered with external beam photons and prescribed at an adequate depth.

Physicians may consider concurrent high dose steroids, preferably Dexamethasone 4 to 8 mg, 2 to 3 times a day during radiotherapy, under cover of proton pump inhibitors according to local protocols.

All patients in the study would receive systemic treatment for metastatic disease according to the local protocols for their primary malignancy, including hormonal treatment, chemotherapy, biological therapy, bisphosphonates, radionuclides or palliative radiotherapy to non-spinal sites. These treatments may be within other clinical trials. The use of other investigational agents is allowed during the study period. Palliative radiotherapy to the spine for pain relief is permitted but patients should be adequately assessed for SCC according to NICE guidelines.

8.1. MRI PROTOCOL

MRI of the spine should be performed on a MRI system with minimum field strength of ≥ 1 Tesla and with a spinal coil. The whole spine should be imaged from the base of skull to the coccyx with sagittal T1 and T2 weighted images. Sagittal images may be supplemented with selected axial images through any suspicious areas at the discretion of the radiologist.

For patients reporting to the physician with symptoms or signs suggestive of neurologic deficit, MRI spine should ideally be performed within 24 hours in accordance with NICE and local guidelines.

All MRIs leading to a diagnosis of rSCC or SCC and a 10% random sample of negative baseline MRIs will be subject to central review. An instruction manual depicting examples of rSCC will be distributed to participating centres.

Additionally, a validated epidural SCC scoring system[53] (Bilsky Spinal Cord Compression Scale (Appendix C)) will be used in all centres. This is well suited for defining rSCC using MRI. This scale showed excellent reproducibility when assessed for inter and intra observer reliability.

Non-trial imaging including isotope scans (CT and MRI) and their impact on detection of SCC will be monitored . The CRF's will be designed to capture non-trial imaging.

8.2. TREATMENT PROTOCOL

Most commonly, we expect radiotherapy to be the treatment of choice for both rSCC and cSCC but the recommendation of the specialist SCC MDT (if available) or expert local practice should be followed. Details of treatment received for any SCC detected on any MRI will be collected on the relevant CRF.

8.2.1 High dose corticosteroids

Following the diagnosis of rSCC or cSCC, high dose corticosteroids may be prescribed (commonly dexamethasone 8-24mgs total dose per day in divided doses with gastric protection) at the investigators discretion. If used, the maximum dose and duration of high dose corticosteroid usage will be recorded. The duration will be from the start of high dose treatment to the time when corticosteroid dose is <3mgs/day of dexamethasone or equivalent.

8.2.2 Radiotherapy

Radiotherapy should be delivered as soon as reasonable after detection of rSCC on screening MRI. For Bilsky 1a-1c graded rSCC treatment should be within one week (maximum 2 weeks) following confirmation of rSCC after review at relevant MDT or other local process.

Patients with Bilsky 2 - 3 rSCC or with clinical symptoms of SCC should be treated within 48 hours of diagnosis in line with NICE Guidance.

<u>Dose and Fractionation</u>
The recommended radiotherapy dose is 20 Gy given daily in 5 fractions prescribed at an adequate depth and at least to the mid-point of the spinal cord /cauda equina for patients who have asymptomatic rSCC.
<u>Planning technique</u>
Radiotherapy may be planned by conventional simulation or by CT based virtual simulation. The findings of the MRI scans should be taken into account while deciding the PTV. Field size will be at least 1 vertebral level above / below site of rSCC and include the pedicles of the vertebrae laterally and should cover the whole of the soft tissue disease with an adequate margin. If at the time of radiotherapy planning there is any doubt of the accuracy of localisation this should be increased to +/- 2 vertebra. If there is additional bony involvement of adjacent vertebra clinical judgement should be used to increase field length. The treatment technique will most commonly be with a single under couched direct posterior field treating with the patient in the supine position. Other techniques may be used as clinically indicated (for example, lateral fields for cervical spine metastases). The prescription point (or depth) should give a dose of at least 20Gy in five fractions to the mid-point of the spinal cord or cauda equina but may be modified to adequately treat soft tissue or bony involvement.

Similar guidelines would be used for clinically established SCC treated with radiotherapy.

8.2.3 Surgery

Additionally the role of spinal surgery should be assessed with the specialist cancer network SCC MDT team (if available) or expert local practice. Any surgical treatment should follow the recommendations of the specialist MDT (if available) or expert local practice and all such treatment details will be recorded. If surgery is performed, most patients will then receive post-operative radiotherapy as above.

Surgery may be considered in the following situations:

1. Patients with spinal instability or pathological spine fractures or evidence of bone in the spinal canal.
2. Patients who have progressed neurologically on radiotherapy.
3. Patients who have relapsed at previously radiated sites.
4. Instances where the treating doctor feels the patient may be benefited by surgical decompression or stabilisation.

The aim of surgery is to provide immediate decompression of the spinal cord. Surgery should be tailored for each patient depending on the level of the spine involved and the patient's circumstances.

Post-operative radiotherapy to sites where decompression or stabilisation surgery has been done should be considered. The radiotherapy dose and plan would be similar to that stated previously.

8.3. ASSESSMENTS DURING EACH EPISODE OF SCC.

The radiologic level of SCC (Bilsky 1a-3), days of inpatient admissions, details of radiotherapy treatment, pre and post treatment neurologic scores, pre and post treatment level of analgesia scores and quality of life scores, neurologic improvement after treatment and concurrent systemic treatments will be collected during each episode of cord compression or treatment for rSCC or cSCC (Bilsky 1a-3).

The incidence of repeat episodes of SCC in treated patients, and the details of any follow up MRI spine will be collected.

8.4. SYSTEMIC TREATMENT

Patients should be managed according to standard local practice and treatments should be similar for patients in both groups of the study. Alternative treatment strategies may be prescribed at the discretion of the responsible physician in accordance with standard local practice. Entry to other clinical trials which do not mandate MRI spine and the use of investigational agents are permitted. Systemic treatments will be recorded on the CRF.

9. EVALUATION OF OUTCOME

9.1. DEFINITIONS OF CLINICAL AND RADIOLOGICAL SCC

Clinical SCC (cSCC) is defined in the NICE metastatic SCC guidelines as “spinal cord or cauda equina compression by direct pressure and/or induction of vertebral collapse or instability by metastatic spread or direct extension of malignancy that threatens or causes neurological disability”. For the purposes of the PROMPTS protocol patients with clinical SCC will be symptomatic with imaging confirming SCC according to the NICE definition. Imaging will be scored according to the Bilsky system (appendix C). Patients with clinical SCC will usually have Bilsky scores of 2 or 3.

Radiologic SCC (rSCC) will be defined by MRI in asymptomatic patients: MRI findings will usually give a Bilsky 1a- 1c score but exceptionally Bilsky 2-3 scores may be found in asymptomatic patients.

Please also refer to page v of the protocol ‘Practical definitions for the purposes of the protocol’.

9.2. DEFINITION OF FINDINGS ON MRI SPINE.

MRI findings in the spine will be classified as either:

i. SCC (radiological or clinical) defined as metastatic disease causing impingement, indentation or loss of definition of the thecal sac or frank compression of spinal cord or cauda equina (Bilsky score 1a – 3)
ii. no SCC.

A validated epidural SCC scoring system[53] (the modified Bilsky Spinal Cord Compression Scale below and in appendix C) will be used by radiologists in participating centres. This scale is well suited for defining rSCC using MRI and showed excellent reproducibility when assessed for inter and intra observer reliability.

0	Metastatic bone disease without epidural impingement
1a	Epidural impingement without deformation of the thecal sac
1b	Deformation of the thecal sac
1c	Deformation of the thecal sac with spinal cord abutment, but without cord compression
2	Spinal cord compression but with cerebrospinal fluid (CSF) visible around the cord
3	Spinal cord compression, no CSF visible around the cord
9	No bone metastasis (additional score for PROMPTS trial)

Practical definitions for the purpose of the protocol

cSCC – Clinical spinal cord compromise or compression – patients are symptomatic

Clinical spinal cord compromise – Bilsky score 1a-c (Bilsky 1a-b would be exceptional)

Clinical spinal cord compression – Bilsky score 2 or 3

rSCC – Radiological spinal canal/cord compromise or compression – patients are asymptomatic

Radiological spinal canal compromise – Bilsky score 1a or 1b

Radiological spinal cord compromise – Bilsky score 1c

Radiological spinal cord compression – Bilsky score 2 or 3

9.3. DEFINITION OF FUNCTIONAL NEUROLOGIC DEFICIT DUE TO SCC.

Functional neurologic deficit is defined as detection of one or more of the following on clinical examination:

1. Objective motor power loss due to involvement of the spinal cord or nerve roots.
2. Objective sensory loss due to involvement of the spinal cord or nerve roots.
3. Urinary incontinence or retention due to involvement of the spinal cord or nerve roots.
4. Bowel incontinence due to involvement of the spinal cord or nerve roots.

Functional neurologic deficit will be assessed and scored according to the Frankel System (see appendix B).

Neurologic deficit due to base of skull involvement or peripheral nerve involvement should be ruled out clinically and would not be considered as an endpoint for the study.

10. CONCURRENT MEDICATIONS

All medication considered necessary for the patients' welfare and which is not expected to interfere with the evaluation of the study intervention may be given at the discretion of the investigator. Only treatment related to the patient's prostate cancer should be recorded on the appropriate pages of the CRF.

11. SAFETY REPORTING

11.1. DEFINITION OF AN ADVERSE EVENT (AE)

An 'adverse event' is any untoward medical occurrence in a patient administered a research procedure; where the events do not necessarily have a causal relationship with the procedure. For the purpose of this trial, any detrimental change in the patient's condition subsequent to the start of the trial (i.e. randomisation) and during any treatment given for rSCC or cSCC, which is not unequivocally due to progression of disease (prostate cancer), should be considered as an adverse event.

Whenever one or more signs and/or symptoms correspond to a disease or well-defined syndrome only the main disease/syndrome should be reported. For each sign/symptom the highest grade observed since the last visit should be reported.

We expect adverse events to be rare in the context of this trial, with the intervention being MRI scanning and therefore, there is no expected list of event occurrences relating to MRI in this protocol. Events such as disease progression, disease relapse or admissions due to these causes, and death as a result of disease relapse are not considered to be SAE's and should be reported on the appropriate CRF. Events related to co-morbid conditions should not be reported or considered as SAEs.

11.2. DEFINITION OF RELATED ADVERSE EVENT

An adverse event assessed by the Principal Investigator (PI) or Chief Investigator (CI) as having a reasonable causal relationship to the administration of the research procedure (MR scan) i.e. is possibly, probably, or definitely related to the research procedure. Any such events are expected to have occurred within 24 hours of the MR scan.

11.3. REPORTING OF ADVERSE EVENTS

Adverse events will be reported during the trial at the time points detailed in section 6 of the protocol. Adverse events should be recorded in the appropriate section of the CRF.

11.4. DEFINITION OF SERIOUS ADVERSE EVENTS (SAES)

Serious Adverse Events will be collected for a 24 hour period only after the study related screening MRI scan. A serious adverse event is one which falls in one of the following categories:

1. **Results in death**; the patient's death is suspected as being a direct outcome of the adverse event.
2. **Is life-threatening;** refers to an event in which the subject was at risk of death at the time of the event; it does not refer to an event that would result which hypothetically might have caused death if it were more severe.
3. **Requires hospitalisation, or prolongation of existing inpatient hospitalisation:** admission to hospital overnight or prolongation of a stay in hospital was necessary as a result of the AE.

Outpatient treatment in an accident and emergency department is not itself an SAE, although the reasons for it may be. Hospital admissions/surgical procedures planned for a pre-existing condition before a patient is randomised to the study are not considered SAEs, unless the illness/disease deteriorates in an unexpected way during the study.

4. **Results in persistent or significant disability or incapacity:** The AE results in a significant or persistent change, impairment, damage or disruption in the patient's body function/structure, physical activities or quality of life.
5. **Consists of a congenital anomaly or birth defect.**

Whenever one or more signs and/or symptoms correspond to a disease or well-defined syndrome, only the main disease/syndrome should be reported. For each sign/symptom the highest grade observed should be reported.

11.5. EVENTS NOT SUBJECT TO EXPEDITED REPORTING:

The following are anticipated treatment, disease or co-morbidity related adverse events which are not subject to expedited reporting. All such events should be reported in the appropriate sections of the CRF.

a) Any radiotherapy treatment related events will be collected on a post radiotherapy complications form.

b) Any surgical treatment related events will be collected on a post-surgery complications form.

c) Disease related events:

- Progressive disease
- Symptoms related to progressive disease
- Death due to disease

11.6. SAE CAUSALITY

The Principal Investigator is responsible for the assessment of causality of serious adverse events as defined in the table below:

Definitions for SAE causality

Relationship	Description
**Unrelated**	There is no evidence of any causal relationship with the trial procedure
**Unlikely**	There is little evidence to suggest there is a causal relationship (e.g. the event did not occur within a reasonable time after the trial procedure. There is another reasonable explanation for the event (e.g. the patient's clinical condition, other concomitant treatment)
**Possible**	There is some evidence to suggest a causal relationship (e.g. because the event occurs within a reasonable time after the trial procedure. However, the influence of other factors may have contributed to the event (e.g. the patient's clinical condition, other concomitant treatments)
**Probable**	There is evidence to suggest a causal relationship, and the influence of other factors is unlikely
**Definitely**	There is clear evidence to suggest a causal relationship, and other possible contributing factors can be ruled out

**Not assessable**	There is insufficient or incomplete evidence to make a clinical judgement of the causal relationship.

11.7. REPORTING SERIOUS ADVERSE EVENTS

SAEs subject to expedited reporting from date of randomisation and up to 24 hours post screening MRI scan must be reported.

All SAEs should be reported within 24 hours of the investigator becoming aware of the event, by completing the PROMPTS SAE form and faxing it to:

PROMPTS Trial Manager

The Safety Desk

Clinical Trials and Statistics Unit (ICR-CTSU)

FAX: 0208 722 4369

(Monday – Friday 09.00 – 17.00)

The SAE form must be completed, signed and dated by the Principal Investigator or nominated person identified on the site delegation log, although initial notification should not be delayed for signature, but should be followed by a report signed and dated by the Principal Investigator or nominated representative as soon as possible. A hard copy must also be sent by post to the trials office using the address on the SAE form. ICR-CTSU office will send a fax to the Site to acknowledge receipt of the SAE.

Any relevant follow up information, including final resolution of the event, should be completed on the relevant part of the original SAE form and faxed to the ICR-CTSU within 15 days of the local investigator becoming aware of this information. The Chief Investigator (or a nominated representative) will review all SAEs to assess relatedness and expectedness. The Site SAE log should be completed and the SAE form filed in the Site Investigator File.

11.8. REVIEW OF SERIOUS ADVERSE EVENTS (SAES)

Reported SAEs will be assessed by the Chief Investigator (or designated representative) for causality and expectedness.

NB. The Chief Investigator cannot down grade the Principal Investigator’s assessment of causality. SAEs assessed as having a causal relationship to the study procedure and as being unexpected (related unexpected SAEs) will undergo expedited reporting to the main REC by ICR-CTSU.

Centres should respond as soon as possible to requests from the Chief Investigator or designated representative (via ICR-CTSU) for further information that may be required for final assessment of the SAE.

11.9. EXPEDITED REPORTING OF RELATED UNEXPECTED SAES

If an SAE is defined as related and unexpected by the Chief Investigator, ICR-CTSU will report the SAE to the main REC within 15 days from the date the Chief Investigator or designated Co-Investigator became aware of the event. Any subsequent reporting will be carried out as appropriate.

11.10. FOLLOW UP OF SERIOUS ADVERSE EVENTS

Centres should continue to follow up SAEs until the event is resolved e.g. recovered, recovered with sequelae, or died. Information on outcome of the SAE should be completed on the relevant

part of the original SAE and faxed to ICR-CTSU as soon as the Principal Investigator becomes aware.

11.11. ANNUAL REPORTING OF SERIOUS ADVERSE EVENTS

An annual report of related unexpected SAEs will be provided to the Main REC, by ICR-CTSU, in the annual progress report at the end of the reporting year. This will be defined as the anniversary of the date when the study received a favourable opinion from the Main REC.

12. STATISTICAL CONSIDERATIONS

The study is aimed at comparing how the experimental intervention (screening MRI and pre-emptive radiotherapy) compares to what we consider to be standard practice which is observation with MRI spine if there is clinically evident neurologic deficit.

For screening MRI to be deemed a better schedule than observation, the proportion of patients who develop neurologic deficit should be significantly less.

12.1. SAMPLE SIZE CALCULATION

414 patients will be randomised in a 1:1 ratio to the control and intervention groups.

The sample size is based on a superiority design using the log-rank comparison of the proportion of patients with cSCC at one year. The incidence of cSCC at one year in the control arm is estimated to be 15.6%. This is based on the following assumptions:

- the baseline prevalence of rSCC is 12.9%. This figure is the average rSCC rate detected by MRI in metastatic CRPC patients with no back pain and no analgesics (i.e. the eligible population for PROMPTS) reported in the non-randomised studies of Bayley[8] (5/29 patients) and Venkitaraman[22] (3/33 patients).
- all patients with rSCC will develop cSCC by one year if untreated
- 3.2% of rSCC "negative" patients will develop cSCC by one year[8] (also untreated).

With 414 patients (71 events) there is 85% power to detect a 50% relative reduction in one year incidence of cSCC (from 15.6% control to 7.8% intervention). This corresponds to a hazard ratio (HR) of 0.48 (with a 5% two-sided alpha).

The sample size assumes uniform accrual over a 4 year period and a minimum of one year of follow-up for all patients. Death is treated as a censoring event assuming a median overall survival of 19 months[49]. No adjustment for non-compliance has been made as eligible patients are not anticipated to withdraw from trial intervention.

It should be noted that the sample size calculations are sensitive to the assumptions made about the prevalence rSCC and the effectiveness of the intervention. The figure of 12.9% comes from two studies in which the subgroup of CRPC patients without back pain and not on analgesics is small and it may be that in the multi-centre phase III trial setting the estimates of SCC rates in both arms are lower than predicted from these studies. With 414 patients, there will be adequate statistical power if observed rSCC rates are lower than those estimated above. As an example, there would be 80% power to detect a 50% relative reduction from 11.5% to 5.7% in 1 year incidence of cSCC.

12.2. ENDPOINT DEFINITIONS

Primary endpoint
Incidence of and time to development of confirmed cSCC. One year incidence is of primary interest. Time will be measured from randomisation.

Confirmed cSCC is defined as per NICE metastatic SCC guidelines i.e. “spinal cord or cauda equina compression by direct pressure and/or induction of vertebral collapse or instability by metastatic spread or direct extension of malignancy that threatens or causes neurological disability”. See section (9.1).

Episodes of SCC suspected clinically will be confirmed by MRI (usually Bilsky score 2 or 3); or rarely by other clinically appropriate imaging when MRI is contraindicated.

Secondary Endpoints

- Rate of detection of rSCC (Bilsky 1a-3) on the baseline screening MRI (in the intervention group only);
- Incidence of and time to functional neurological deficit due to clinical SCC - Neurological deficit will be assessed using the Frankel spinal cord injury assessment tool. This indicates the grade of neurological and ambulatory compromise on a 5 point scale and has been used in other studies of metastatic spinal disease (appendix B). Functional neurological deficit will be defined as Frankel Score A-D.
- Incidence of and time to irreversible functional neurological deficit (irreversible defined as no improvement after 3 and 6 months following first incidence).
- Incidence of SCC (Bilsky 1-3) in both the control and intervention groups during follow-up.
- Pain - this will be measured using the Brief Pain Inventory (short form).
- Quality of life – this will be measured using the standard EORTC QLQ C30, EQ5D-5L and HADS questionnaires
- Overall survival – will include deaths from any cause
- Cost effectiveness.

12.3. STATISTICAL ANALYSIS

Primary analysis set

Analyses of outcome data will be on the basis of intention to treat and therefore include all patients who deviate from trial protocol for the following reasons: ineligibility for trial intervention, unwillingness to continue with follow-up visits, withdrawal of consent after randomisation, deviation from allocated intervention and loss to follow-up.

Analysis methods

The primary analysis will be based a log-rank intention to treat comparison of cSCC incidence in the intervention and control group. Time to cSCC, time to neurological deficit, and overall survival will be analysed using Kaplan-Meier estimates and the log-rank test, with event rates at 1 and 2 years reported for each group. A Cox model will be used to adjust for important prognostic factors including alkaline phosphatase (normal vs. raised), number of previous treatments (first line failure vs. 2nd or later), use of previous spinal radiotherapy and/or surgical procedure for metastatic disease (yes/no), number of spinal metastases on bone scan, time since development of CRPC, time since start of continuous hormone treatment, performance status and PSA or PSA doubling time. Alternatives such as a piecewise-constant HR will be considered if the proportional hazards assumption is not appropriate. The use of cumulative incidence curves, Gray’s test and a Fine & Gray model for time to cSCC treating death as a competing risk will also be explored.

The proportion of patients having MR and total number of MR scans performed will be reported for both groups. The observed baseline rate of rSCC will be presented for the intervention group with a 95% confidence interval; subsequent rates of rSCC detected by the 6-month screening schedule in patients with rSCC identified and treated at baseline will be reported separately. A multiple logistic regression model will be used to identify clinical predictors of rSCC in the baseline MRI.

The primary analysis will be event driven and will be conducted once all patients have been followed up for at least one year. A formal interim analysis will be conducted after 54 patients have been recruited to the intervention group (estimated to be approximately 6 months after start of

recruitment). The timing of subsequent analyses for the Independent Data Monitoring Committee (IDMC) review will be at the discretion of the IDMC but meetings are planned at least annually.

There are no pre-planned subgroup analyses.

Quality of life (QL) assessment will be conducted by questionnaire (EORTC QLQ C30, HADS and EQ-5D-5L) and will be assessed at baseline and 3, 6, 12, 15, 18, 21, 24, 30 and 36 months.

The main scales of interest are functional QL, global health QL and pain. According to the EORTC reference manual[50], for the physical functioning subscale a difference of 8 points is considered clinically relevant and standard deviation for metastatic prostate cancer is 21.9 points. Using a two-sided 5% significance level there is 90% power to detect an 8-point difference in this subscale with 159 patients per group (this requires only 59% participation in QL study). The primary endpoint will be physical functioning from the EORTC QLQ C30.

Analysis of QL will include between group comparisons at individual time points. Methods to model changes over time, such as generalised estimating equations, will be explored. Scales of interest will be analysed using total scale score (e.g. ANCOVA of change from baseline); dichotomisation of scales or individual items of relevance will also be considered where clinically relevant, analysed by chi-square-based or Fisher's exact test as appropriate. To account for multiple testing, only p-values 0.01 will be considered statistically significant on endpoints other than the primary QL endpoint.

Pain will be measured using the Brief Pain Inventory (BPI) Short form assessed at the same timepoints as QL. The BPI will be analysed according to standard methods[51] Pain severity will be presented as mean scores for each of the individual scales of "worst", "least", "average" and "now" at each time point. A mean pain severity score (a composite of all 4 pain severity scores) will also be presented comparisons made between randomised groups at each time point. Pain interference will be presented as mean scores for the 7 individual daily activities (general activity, mood, walking ability, normal work, relations, sleep and enjoyment of life) at each time point. A mean pain interference score (a composite of the 7 daily activities) will also be presented and compared between randomised groups at each time point. If at least 4/7 individual pain severity scores have been given then a mean score will be calculated. To account for multiple testing, only p-values below 0.01 will be considered statistically significant.

Stopping Rules and Interim Analysis

An early stopping rule based on detecting a minimum level of rSCC is proposed.

For there to be an opportunity to prevent cSCC with pre-emptive treatment, rSCC needs to be detected. If the "pick-up" rate is very low, the opportunity to prevent SCC is small and the intervention is unlikely to be cost-effective (at a population level). The rSCC pick up rate will be continuously monitored throughout the course of the trial by the Independent Data Monitoring Committee and the Trial Steering Committee It will ultimately be for the TSC to agree the lowest acceptable rate of detecting rSCC which would warrant continuing with the study. As an example, it would be considered worthwhile screening patients with MRI if the rSCC pick-up rate is 1 in 10 (10%) but not if it is as low as 1 in 40 (2.5%). Under these assumptions, an interim analysis would be carried out to assess the detection rate of rSCC after 54 patients had been recruited to the intervention group. If less than 4 cases of rSCC had been identified (ruling out a lower limit of the confidence interval of 2.5% with 80% power and a one-sided alpha of 5% if the true rate is 10%), then the TSC would be asked to advise on continuing recruitment to the study having considered all internal and external evidence.

No early stopping rules for toxicity are proposed.

Recruitment milestones will be set in discussion with the TSC. It is proposed that recruitment rates are closely monitored and subject to formal review by the TSC at 12 and 18 months after five centres have opened and have recruited at least one patient with subsequent recruitment conditional on demonstrating that the target sample size is achievable within agreed timelines.
An IDMC will review recruitment rates, safety and emerging efficacy data in confidence at least annually.

13. TRIAL MANAGEMENT

13.1. TRIAL MANAGEMENT GROUP (TMG)

A Trial Management Group (TMG) will be set up and will include the Chief Investigator, Co-investigators and identified collaborators, the Trial Statistician and the Trial Manager. Principal Investigators and key study personnel will be invited to join the TMG as appropriate to ensure representation from a range of centres and professional groups. Notwithstanding the legal obligations of the Co-Sponsors and Chief Investigator, the TMG have operational responsibility for the conduct of the trial. Where possible, membership will include a lay/consumer representative. The Committee's terms of reference, roles and responsibilities will be defined in a charter issued by ICR-CTSU.

13.2. TRIAL STEERING COMMITTEE (TSC)

This study will fall under the governance of the ICR-CTSU Prostate Radiotherapy Trial Steering Committee. This group will include an independent Chairman (not involved directly in the trial other than as a member of the TSC), not less than two other independent members, the Chief Investigator and the ICR-CTSU Scientific Lead. It is the role of the TSC to monitor progress of the trial and to ensure there is adherence to the protocol and the principles of Good Clinical Practice. The Committee's terms of reference, roles and responsibilities will be defined in a charter issued by ICR-CTSU.

13.3. INDEPENDENT DATA MONITORING COMMITTEE (IDMC)

An IDMC will be instigated to monitor the progress of the trial. Membership of the IDMC will be proposed by the TMG and approved by the TSC. The Committee's terms of reference, roles and responsibilities will be defined in a charter issued by ICR-CTSU. The IDMC should meet in confidence at regular intervals, and at least annually. A report of the findings and recommendations will be produced following each meeting. This report will be submitted to the TMG and TSC, and if required, the main REC.

The IDMC reserve the right to release any data on outcome or side-effects through the TSC to the TMG (and if appropriate to participants) if it determines at any stage that the combined evidence from this and other studies justifies it.

14. RESEARCH GOVERNANCE

The Institute of Cancer Research (ICR) is the sponsor of this trial in accordance with the Research Governance Framework for Health and Social Care and the principals of Good Clinical Practice (GCP).

14.1. SPONSOR RESPONSIBILITIES

The Institute of Cancer Research has sponsorship responsibility for obtaining authorisation and appropriate ethics committee opinion.

14.2. RESPONSIBILITIES OF CHIEF INVESTIGATOR

The Chief Investigator is responsible for:

- Selection of investigators

- Prompt decision as to which serious adverse events are related and unexpected; and
- Prompt reporting of that decision to the ICR-CTSU, for onward reporting to the main REC.

14.3. RESPONSIBILITIES OF ICR-CTSU

ICR-CTSU has overall responsibility for facilitating and co-ordinating the conduct of the trial and is also responsible for collating data obtained, and undertaking and reporting interim and final analyses.

The responsibilities of ICR-CTSU for the day-to-day management of the trial will include the following:

- Ensuring an appropriate ethics opinion has been sought, and any amendments have been approved.
- Giving notice of amendments to protocol, making representations about amendments to the main REC.
- Giving notice that the trial has ended.
- Randomising patients.
- Collating QL questionnaires returned by post.
- Raising and resolving queries with local investigators.
- Logging clinical and QL data received; raising queries.
- Keeping records of all serious adverse events (SAEs) reported by investigators.
- Notifying the main REC and Investigators of related unexpected Serious Adverse Events.

14.4. RESPONSIBILITIES OF PARTICIPATING CENTRES

Centres wishing to recruit to this study will be asked to provide evidence that they can deliver protocol treatment. This will include:

- Putting and keeping in place arrangements to adhere to the principles of GCP.
- Keeping a copy of all ‘essential documents’ (as defined under the principles of GCP) and ensuring appropriate archiving of documentation once the trial has ended.
- Providing evidence that local practice conforms to the standards set in the NICE Improving Outcomes Guidance;

Responsibilities are defined in an agreement between an individual participating centre and The Institute of Cancer Research.

15. TRIAL ADMINISTRATION AND LOGISTICS

15.1. PROTOCOL COMPLIANCE

The PROMPTS trial is being conducted in accordance with the professional and regulatory standards required for non-commercial research in the NHS under the Research Governance Framework for Health and Social Care and the principles of GCP. Before activating the trial, participating centres are required to sign an agreement accepting responsibility for all trial activity which takes place within their centre. Sites may only commence recruitment once centre agreements have been signed by both parties, trial documentation is in place and a site initiation (visit or teleconference) has taken place. Site initiation visits will be conducted at sites where the Principal Investigator has requested one or where ICR-CTSU deems it is appropriate.

15.2. INVESTIGATOR TRAINING

Prior to commencing trial recruitment, training and advice will be provided by members of the Trial Management Group via a trial launch meeting, training workshops, and QA feedback to identified key individuals in each participating centre. Training will include discussion on the background to

the study and discussion on the issues of clinical equipoise. Participating centres will be asked to maintain a screening log to monitor randomisation acceptance rates, and additional support/training will be offered when lower than anticipated rates are encountered.

15.3. DATA ACQUISITION

The clinical data should be recorded on the PROMPTS case report forms (CRFs) and the relevant pages forwarded to ICR-CTSU in a timely manner. The Trial Management Group reserves the right to amend or add to the CRFs as appropriate. Such changes do not constitute a protocol amendment, and revised or additional forms should be used by centres in accordance with the guidelines provided by ICR-CTSU. Where appropriate, data may need to be collected retrospectively if an additional question has been added to the CRF.

By participating in the PROMPTS trial, the Principal Investigators at each centre are confirming agreement with his/her local NHS Trust to ensure that:

- Sufficient data is recorded for all participating patients to enable accurate linkage between hospital records and CRFs;
- Source data and all trial related documentation are accurate, complete, maintained and accessible for monitoring and audit visits;
- Original consent forms are dated and signed by both patient and investigator and are kept together in a central log together with a copy of the specific patient information sheet(s) given at the time of consent;
- All essential documents are retained for five years after the trial ends to comply with current legislation.
- Staff will comply with the protocol and Trial Guidance Notes for PROMPTS.

On receipt at ICR-CTSU, CRFs will be recorded as received and any missing forms will be reported to the originating site. Illegible forms may be returned to site for clarification.

15.4. CENTRAL DATA MONITORING

ICR-CTSU will review incoming CRFs for compliance with the protocol, and for inconsistent or missing data. Should any missing data or data anomalies be found, queries will be sent to the relevant centre for resolution. Following initial review, the CRF data items will be entered into the clinical study database held at ICR-CTSU.

Data will be further reviewed for data anomalies / missing data, by central statistical monitoring. Any systematic inconsistencies identified may trigger monitoring visits to centres.

15.5. ON SITE MONITORING

If a monitoring visit is required, ICR-CTSU will contact the centre to discuss dates of proposed visit. Once a date has been confirmed, the centre should ensure that the relevant patient notes are available for monitoring.

If any problems are detected in the course of the monitoring visit, ICR-CTSU will work with the Principal Investigator to resolve issues and, if necessary, to determine the centre's future participation in the study.

ICR-CTSU staff conducting on-site monitoring will review essential documentation and carry out source data verification to confirm compliance with the site agreement and trial protocol to ensure the protection of patients' rights as detailed in the Declaration of Helsinki 1964 as amended October 1996.

15.6. PROTOCOL AMENDMENTS

Proposed major protocol amendments will be submitted to the TMG by the Chief Investigator. The TMG will agree protocol amendments prior to submission to the Main REC. Once approved the Principal Investigator at each centre will be informed of the change and sent all the associated documentation. It is the Principal Investigator's responsibility to submit amendments to their R&D department for approval. Confirmation that this has been done must be provided to ICR-CTSU.

15.7. END OF STUDY

For the purposes of ethics approval, the study end date is deemed to be the date of the last data capture.

15.8. ARCHIVING

Essential documents are documents that individually and collectively permit evaluation of the conduct of the trial and substantiate the quality of the data collected. Essential documents will be maintained at ICR-CTSU in a way that will facilitate the management of the trial, audit and inspection. They should be retained for a sufficient period (at least 15 years) for possible audit. Documents should be securely stored and access restricted to authorised personnel.

16. PATIENT PROTECTION AND ETHICAL CONSIDERATIONS

16.1. RISK ASSESSMENT

This study has been formally assessed for clinical risk using a generic risk assessment.

16.2. PATIENT CONFIDENTIALITY

Patients will be asked to consent to their full name being collected at randomisation in addition to their date of birth, hospital number, postcode and NHS number (CHI in Scotland) to allow tracing through their GP and national records to assist with long term follow up. The personal data recorded on all documents will be regarded as confidential, and any information which would allow individual patients to be identified will not be released into the public domain.

The Principal Investigator must maintain in strict confidence trial documents, which are to be held in the local centre (e.g. patients' written consent forms). The Principal Investigator must ensure the patient's confidentiality is maintained.

ICR-CTSU will maintain the confidentiality of all patients and will not reproduce or disclose any information by which patients could be identified. Representatives of ICR-CTSU and the regulatory authorities will be required to have access to patients notes for quality assurance purposes but patients should be reassured that their confidentiality will be respected at all times. In the case of special problems it is also necessary to have access to the complete study records provided that patient confidentiality is protected.

16.3. ETHICAL CONSIDERATIONS

It is the responsibility of the Chief Investigator to obtain a favourable ethical opinion (main REC approval). It is the responsibility of the Principal Investigator at each participating centre to obtain site-specific approval of the trial protocol and any subsequent amendments. All correspondence with the local REC should be filed by the Principal Investigator in the Site Investigator File.

Patients will be approached about participation in PROMPTS by a member of their clinical care or research team. They will receive a verbal explanation of the trial, together with a Patient Information Sheet which they will take home with them. It is the responsibility of the Principal Investigator to give each patient, prior to inclusion in the trial, full and adequate verbal and written information regarding the objective and procedures of the trial and the possible risks involved.

Sufficient time (a minimum of 24 hours) should be allowed for the patient to decide on trial entry. Patients must be informed about their right to withdraw from the trial at any time. Written patient information must be given to each patient before enrolment. They will be given at least 24 hours to make a decision about whether they would like to participate, during which time they will be able to discuss their options with friends, family or their GP. They will have the opportunity to raise any questions about PROMPTS with their clinical care or research team and these will be addressed prior to their decision about whether to participate. The written patient information is an approved patient information sheet according to national guidelines. This also outlines the QL study. Patients will be encouraged to participate in this associated study but if they subsequently decline, this will not exclude them from the main trial.

It is the responsibility of the Principal Investigator or designated representative, to obtain signed informed consent from all patients prior to inclusion in the trial.

16.4. PATIENT INFORMATION

The importance of providing a high level of information to patients is recognised. Local leaflets on radiotherapy should be provided by each centre, but these must be approved by the appropriate local research committees before distribution.

16.5. DATA SHARING

Data arising from this research will be managed and made available to maximise public benefit. Data sharing will be in a timely and responsible manner. Appropriate regulatory permissions relating to the ethical use of data must be in place before the data can be shared. Requests to use trial data must be submitted in writing to the ICR-CTSU for approval by the TMG and TSC.

16.6. DATA PROTECTION ACT (DPA)

ICR-CTSU will comply with all aspects of the DPA 1998. Any requests from patients for access to data about them held at ICR-CTSU should be directed to the Trial Manager in the first instance who will refer the request to the Data Protection Officer at The Institute of Cancer Research.

16.7. LIABILITY/INDEMNITY/INSURANCE

Indemnity for participating hospitals is provided by the usual NHS indemnity arrangements.

17. FINANCIAL MATTERS

The trial is investigator designed and led and has been approved by the Clinical Trials Advisory & Awards Committee (CTAAC) of Cancer Research UK, and meets the criteria for R&D support as outlined in the Statement of Partnership on Non-Commercial R&D in the NHS in England.

The trial has received funding from Cancer Research UK. If further funding is received from any other source this will be made apparent in the patient information sheet and to the approving Main REC and CTAAC.

National RTQA and NCRN (or regional equivalent) network resources should be made available for PROMPTS, as the trial is part of the NIHR portfolio by virtue of its approval by CTAAC.

18. PUBLICATION POLICY

The main trial results will be published in a peer-reviewed journal, on behalf of all collaborators. The manuscript will be prepared by a writing group, appointed from amongst the Trial Management Group, and participating clinicians. All participating centres and clinicians will be acknowledged in this publication together with staff from the ICR-CTSU. All presentations and publications relating to the trial must be authorised by the Trial Management Group, on whose behalf publications should usually be made. Authorship of any secondary publications will reflect the intellectual and

time input into these, and will not necessarily be the same as on the primary publication. No investigator may present or attempt to publish data relating to the PROMPTS trial without prior permission from the Trial Management Group.

19. ASSOCIATED STUDIES

19.1. QUALITY OF LIFE

Quality of life (QL) assessment will be conducted by questionnaire (EORTC QLQ C30, EQ-5D-5L and HADS) and will be assessed at baseline and 3, 6, 12, 15, 18, 21, 24, 30 and 36 months. Participation in the QL study is not optional. Due to the relatively poor health of participants and the potential for rapid decline, questionnaires will be handed out in clinic (so as to avoid the situation where a questionnaire is sent centrally from the CTU to a patient with very short life expectancy or recently deceased). Pain will be measured using the Brief Pain Inventory (BPI) Short form assessed at the same timepoints as QL.

19.2. HEALTH ECONOMICS

An economic evaluation will be integrated into the design of the trial, and supplemented with decision modelling as the benefits of intervention are likely extend beyond the duration of the trial. The specific aim of the evaluation will be to compare the cost-effectiveness of screening MRI & pre-emptive treatment against standard practice i.e. MRI spine performed if patients develop clinical neurological deficit or significant spinal pain and subsequent treatment if there is overt SCC. The type of economic evaluation will be a cost-utility analysis, estimated using quality-adjusted life-years (QALYs). The analysis will be performed from a NHS and personal social services cost perspective. Resource use data to be collected alongside the RCT will include those relating to the screen, treatment and all aspects of health care in and outside of the treating centre. Resources directly attributable to the trial protocol will be excluded from the analysis. Health resources will be valued using nationally available NHS cost data. Regression methods will be used to account for missing trial data and censoring, and costs and QALYs occurring after 1 year will be discounted at 3.5% per annum. Results will be presented as mean costs, mean QALYs along with 95% confidence intervals, and the probability that the intervention is cost-effective at different levels of willingness to pay for a QALY gained. Utilities will be calculated using the EQ-5D-5L. Sensitivity analysis will test whether the results are robust to methodological assumptions.
The PROMPTS Resource Use Questionnaire will be given to all patients who have developed spinal cord compression (radiological and clinical). This questionnaire will look at the changes that have been made or are planned to the house in which the patient lives. The questionnaire will be optional and if the patient does not wish to take part in the sub-study then they can still take part in the main PROMPTS trial.

References

1. NICE guideline: Metastatic spinal cord compression: Diagnosis and management of patients at risk of or with metastatic spinal cord compression. National Institute of Health and Clinical Excellence CG75, 2008

2. Loblaw DA, Perry J, Chambers A, et al: Systematic review of the diagnosis and management of malignant extradural spinal cord compression: the Cancer Care Ontario Practice Guidelines Initiative's Neuro-Oncology Disease Site Group. J Clin Oncol 23:2028-37., 2005

3. Maranzano E, Trippa F, Chirico L, et al: Management of metastatic spinal cord compression. Tumori 89:469-75, 2003

4. Levack P, Graham J, Collie D, et al: Don't wait for a sensory level--listen to the symptoms: a prospective audit of the delays in diagnosis of malignant cord compression. Clin Oncol (R Coll Radiol) 14:472-80, 2002

5. Rades D, Stalpers LJ, Veninga T, et al: Evaluation of functional outcome and local control after radiotherapy for metastatic spinal cord compression in patients with prostate cancer. J Urol 175:552-6, 2006

6. Loblaw DA, Laperriere NJ, Mackillop WJ: A population-based study of malignant spinal cord compression in Ontario. Clin Oncol (R Coll Radiol) 15:211-7, 2003

7. Tazi H, Manunta A, Rodriguez A, et al: Spinal cord compression in metastatic prostate cancer. Eur Urol 44:527-32., 2003

8. Bayley A, Milosevic M, Blend R, et al: A prospective study of factors predicting clinically occult spinal cord compression in patients with metastatic prostate carcinoma. Cancer 92:303-10., 2001

9. Cook AM, Lau TN, Tomlinson MJ, et al: Magnetic resonance imaging of the whole spine in suspected malignant spinal cord compression: impact on management. Clin Oncol 10:39-43, 1998

10. Rades D, Dunst J, Schild SE: The first score predicting overall survival in patients with metastatic spinal cord compression. Cancer 112:157-61, 2008

11. Rades D, Fehlauer F, Schulte R, et al: Prognostic factors for local control and survival after radiotherapy of metastatic spinal cord compression. J Clin Oncol 24:3388-93, 2006

12. Maranzano E, Latini P: Effectiveness of radiation therapy without surgery in metastatic spinal cord compression: final results from a prospective trial. Int J Radiat Oncol Biol Phys 32:959-67, 1995

13. Osborn JL, Getzenberg RH, Trump DL: Spinal cord compression in prostate cancer. J Neurooncol 23:135-47, 1995

14. Husband DJ: Malignant spinal cord compression: prospective study of delays in referral and treatment. Bmj 317:18-21, 1998

15. Turner S, Marosszeky B, Timms I, et al: Malignant spinal cord compression: a prospective evaluation. Int J Radiat Oncol Biol Phys 26:141-6, 1993

16. Helweg-Larsen S, Sorensen PS, Kreiner S: Prognostic factors in metastatic spinal cord compression: a prospective study using multivariate analysis of variables influencing survival and gait function in 153 patients. Int J Radiat Oncol Biol Phys 46:1163-9, 2000

17. Helweg-Larsen S: Clinical outcome in metastatic spinal cord compression. A prospective study of 153 patients. Acta Neurol Scand 94:269-75, 1996

18. Solberg A, Bremnes RM: Metastatic spinal cord compression: diagnostic delay, treatment, and outcome. Anticancer Res 19:677-84, 1999

19. Leviov M, Dale J, Stein M, et al: The management of metastatic spinal cord compression: a radiotherapeutic success ceiling. Int J Radiat Oncol Biol Phys 27:231-4, 1993

20. Bach F, Larsen BH, Rohde K, et al: Metastatic spinal cord compression. Occurrence, symptoms, clinical presentations and prognosis in 398 patients with spinal cord compression. Acta Neurochir (Wien) 107:37-43, 1990

21. Rades D, Veninga T, Stalpers LJ, et al: Improved Posttreatment Functional Outcome is Associated with Better Survival in Patients Irradiated for Metastatic Spinal Cord Compression. Int J Radiat Oncol Biol Phys 67:1506-9, 2007

22. Venkitaraman R, Sohaib SA, Barbachano Y, et al: Detection of occult spinal cord compression with magnetic resonance imaging of the spine. Clin Oncol (R Coll Radiol) 19:528-31, 2007

23. Lu C, Gonzalez RG, Jolesz FA, et al: Suspected spinal cord compression in cancer patients: a multidisciplinary risk assessment. J Support Oncol 3:305-12., 2005

24. Talcott JA, Stomper PC, Drislane FW, et al: Assessing suspected spinal cord compression: a multidisciplinary outcomes analysis of 342 episodes. Support Care Cancer 7:31-8., 1999

25. Venkitaraman R, Barbachano Y, Dearnaley DP, et al: Outcome of early detection and radiotherapy for occult spinal cord compression. Radiother Oncol 85:469-72, 2007

26. Husband DJ, Grant KA, Romaniuk CS: MRI in the diagnosis and treatment of suspected malignant spinal cord compression. Br J Radiol 74:15-23., 2001

27. Godersky JC, Smoker WR, Knutzon R: Use of magnetic resonance imaging in the evaluation of metastatic spinal disease. Neurosurgery 21:676-80., 1987

28. Sorensen S, Helweg-Larsen S, Mouridsen H, et al: Effect of high-dose dexamethasone in carcinomatous metastatic spinal cord compression treated with radiotherapy: a randomised trial. Eur J Cancer 30A:22-7, 1994

29. Vecht CJ, Haaxma-Reiche H, van Putten WL, et al: Initial bolus of conventional versus high-dose dexamethasone in metastatic spinal cord compression. Neurology 39:1255-7, 1989

30. Maranzano E, Latini P, Beneventi S, et al: Radiotherapy without steroids in selected metastatic spinal cord compression patients. A phase II trial. Am J Clin Oncol 19:179-83, 1996

31. Heimdal K, Hirschberg H, Slettebo H, et al: High incidence of serious side effects of high-dose dexamethasone treatment in patients with epidural spinal cord compression. J Neurooncol 12:141-4, 1992

32. Loblaw DA, Laperriere NJ: Emergency treatment of malignant extradural spinal cord compression: an evidence-based guideline. J Clin Oncol 16:1613-24, 1998

33. Rades D, Veninga T, Stalpers LJ, et al: Prognostic factors predicting functional outcomes, recurrence-free survival, and overall survival after radiotherapy for metastatic spinal cord compression in breast cancer patients. Int J Radiat Oncol Biol Phys 64:182-8, 2006

34. Pigott KH, Baddeley H, Maher EJ: Pattern of disease in spinal cord compression on MRI scan and implications for treatment. Clin Oncol (R Coll Radiol) 6:7-10, 1994

35. Kaplan ID, Valdagni R, Cox RS, et al: Reduction of spinal metastases after preemptive irradiation in prostatic cancer. Int J Radiat Oncol Biol Phys 18:1019-25., 1990

36. Helweg-Larsen S, Hansen SW, Sorensen PS: Second occurrence of symptomatic metastatic spinal cord compression and findings of multiple spinal epidural metastases. Int J Radiat Oncol Biol Phys 33:595-8, 1995

37. Soerdjbalie-Maikoe V, Pelger RC, Lycklama a Nijeholt GA, et al: Strontium-89 (Metastron) and the bisphosphonate olpadronate reduce the incidence of spinal cord compression in patients with hormone-refractory prostate cancer metastatic to the skeleton. Eur J Nucl Med Mol Imaging 29:494-8, 2002

38. Rades D, Stalpers LJ, Hulshof MC, et al: Comparison of 1 x 8 Gy and 10 x 3 Gy for functional outcome in patients with metastatic spinal cord compression. Int J Radiat Oncol Biol Phys 62:514-8, 2005

39. Rades D, Fehlauer F, Stalpers LJ, et al: A prospective evaluation of two radiotherapy schedules with 10 versus 20 fractions for the treatment of metastatic spinal cord compression: final results of a multicenter study. Cancer 101:2687-92, 2004

40. Rades D, Karstens JH, Alberti W: Role of radiotherapy in the treatment of motor dysfunction due to metastatic spinal cord compression: comparison of three different fractionation schedules. Int J Radiat Oncol Biol Phys 54:1160-4, 2002

41. Rades D, Lange M, Veninga T, et al: Preliminary results of spinal cord compression recurrence evaluation (score-1) study comparing short-course versus long-course radiotherapy for local control of malignant epidural spinal cord compression. Int J Radiat Oncol Biol Phys 73:228-34, 2009

42. Maranzano E, Latini P, Beneventi S, et al: Comparison of two different radiotherapy schedules for spinal cord compression in prostate cancer. Tumori 84:472-7, 1998

43. Chow E, Harris K, Fan G, et al: Palliative radiotherapy trials for bone metastases: a systematic review. J Clin Oncol 25:1423-36, 2007

44. Klimo P, Jr., Thompson CJ, Kestle JR, et al: A meta-analysis of surgery versus conventional radiotherapy for the treatment of metastatic spinal epidural disease. Neuro Oncol 7:64-76, 2005

45. Milross CG, Davies MA, Fisher R, et al: The efficacy of treatment for malignant epidural spinal cord compression. Australas Radiol 41:137-42, 1997

46. Patchell RA, Tibbs PA, Regine WF, et al: Direct decompressive surgical resection in the treatment of spinal cord compression caused by metastatic cancer: a randomised trial. Lancet 366:643-8, 2005

47. Harris JK, Sutcliffe JC, Robinson NE: The role of emergency surgery in malignant spinal extradural compression: assessment of functional outcome. Br J Neurosurg 10:27-33, 1996

48. Chataigner H, Onimus M: Surgery in spinal metastasis without spinal cord compression: indications and strategy related to the risk of recurrence. Eur Spine J 9:523-7, 2000

49 Tannock I.F. et al. Docetaxel plus prednisone or mitoxantrone plus prednisone for advance prostate cancer. NEJM 351(15): 1502-12, 2004

50. Scott et al. EORTC QLQ-C30 Reference Values. EORTC Quality of Life Group, July 2008.

51.http://www.mdanderson.org/education-and-research/departments-programs-and-labs/departments-and-divisions/symptom-research/symptom-assessment-tools/BPI_UserGuide.pdf)

52. http://info.cancerresearchuk.org/cancerstats/incidence/commoncancers/

53. Bilsky et al: Reliability analysis of the epidural spinal cord compression scale. J Neurosurg Spine 13:324–328, 2010

54. Venkitaraman R et al. Frequency of screening magnetic resonance imaging to detect occult spinal cord compromise and to prevent neurological deficit in metastatic castration-resistant prostate cancer. Clin Oncol (R Coll Radiol). Mar;22(2):147-52.) 2010

Appendix A - ECOG Scale

0 Fully active, able to carry out all pre-disease performance without restriction.

1 Restricted in physically strenuous activity, but ambulatory and able to carry out work of a light or sedentary nature, (e.g. light housework, office work).

2 Ambulatory and capable of all self-care, but unable to carry out any work activities. Up and about more than 50% of waking hours.

3 Capable of only limited self-care, confined to bed or chair more than 50% of waking hours.

4 Completely disabled. Cannot carry out any self-care. Totally confined to bed or chair.

5 Dead.

Appendix B – Frankel Spinal Cord Injury Assessment Tool

Grade A	Complete neurological injury - no motor or sensory function clinically detected below the level of the injury.

Grade B	Preserved sensation only - no motor function clinically detected below the level of the injury; sensory function remains below the level of the injurybut may include only partial function (sacral sparing qualifies as preserved sensation).
Grade C	Preserved motor non-functional - some motor function observed below the level of the injury, but is of no practical use to the patient.
Grade D	Preserved motor function - useful motor function below the level of the injury; patient can move lower limbs and walk with or without aid, but does not have a normal gait or strength in all motor groups.
Grade E	Normal motor - no clinically detected abnormality in motor or sensory function with normal sphincter function; abnormal reflexes and subjective sensory abnormalities may be present.

Appendix C – Bilsky Spinal Cord Compression Scale[53] (modified)

0	Metastatic bone disease without epidural impingement
1a	Epidural impingement without deformation of the thecal sac
1b	Deformation of the thecal sac
1c	Deformation of the thecal sac with spinal cord abutment, but without cord compression
2	Spinal cord compression but with cerebrospinal fluid (CSF) visible around the cord
3	Spinal cord compression, no CSF visible around the cord
9	No bone metastasis (additional score for PROMPTS trial)
